# Supplementary figures and images for: A phosphate-binding pocket in cyclin B3 is essential for XErp1/Emi2 degradation in meiosis I (part 1 of 2)
Source: EMBO Rep. 2025 Jan 2;26(3):768–90. doi: 10.1038/s44319-024-00347-8 (PMC11811201; doi:10.1038/s44319-024-00347-8)

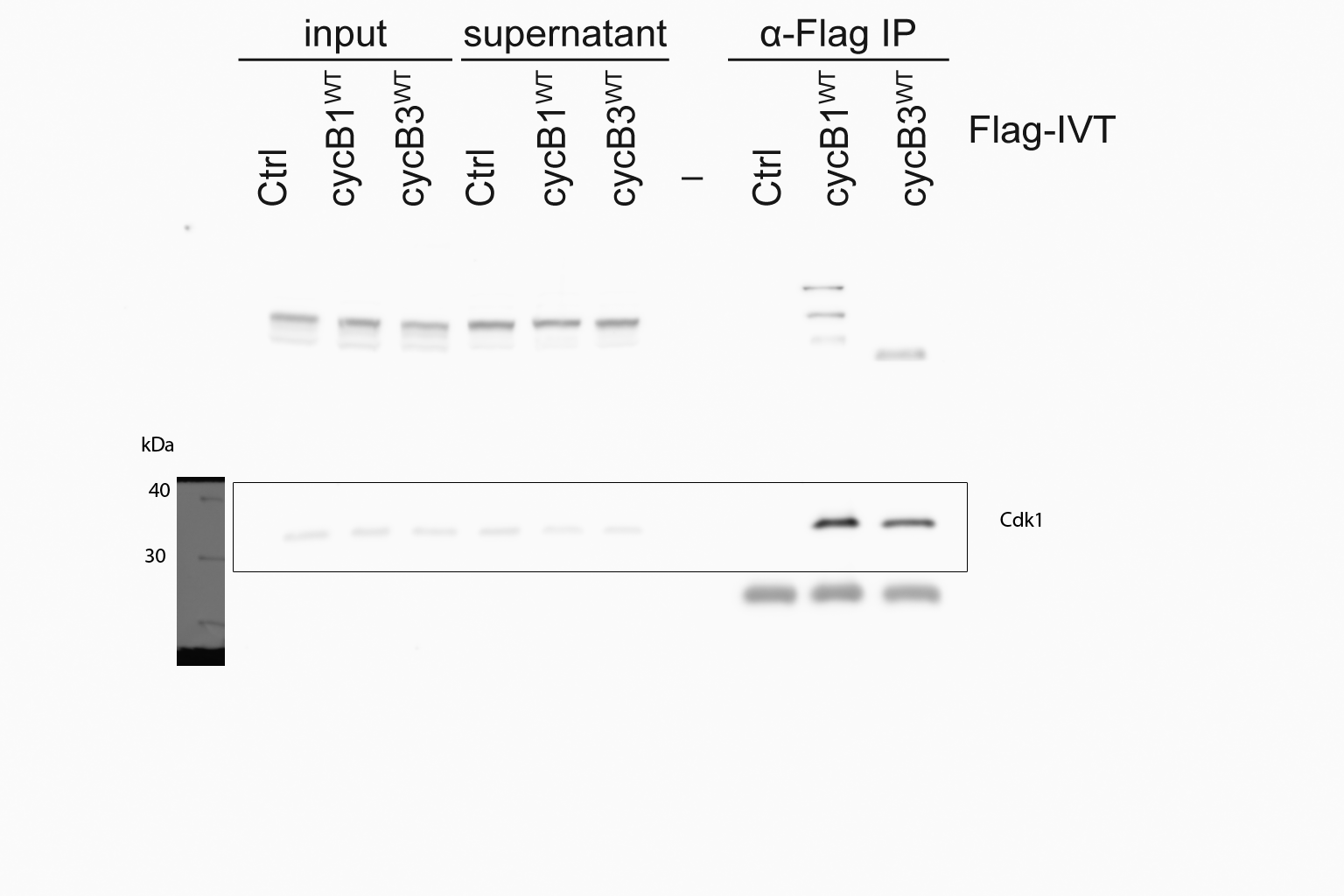

Supplement: Supplementary file 2 — Source data Fig. 1 [file 44319_2024_347_MOESM2_ESM.zip › Figure 1/1C/Western Cdk1.tif]

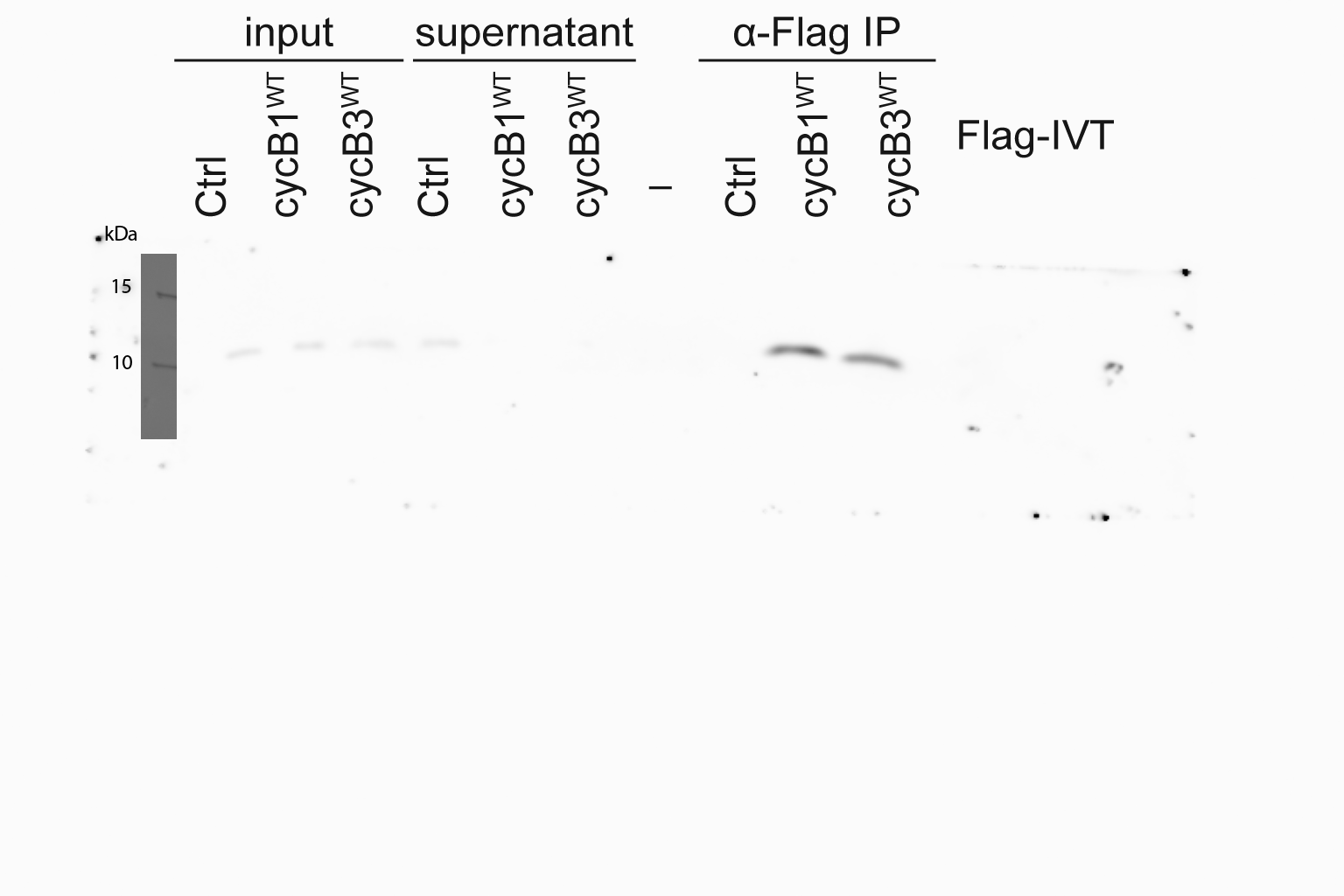

Supplement: Supplementary file 2 — Source data Fig. 1 [file 44319_2024_347_MOESM2_ESM.zip › Figure 1/1C/Western Cks2.tif]

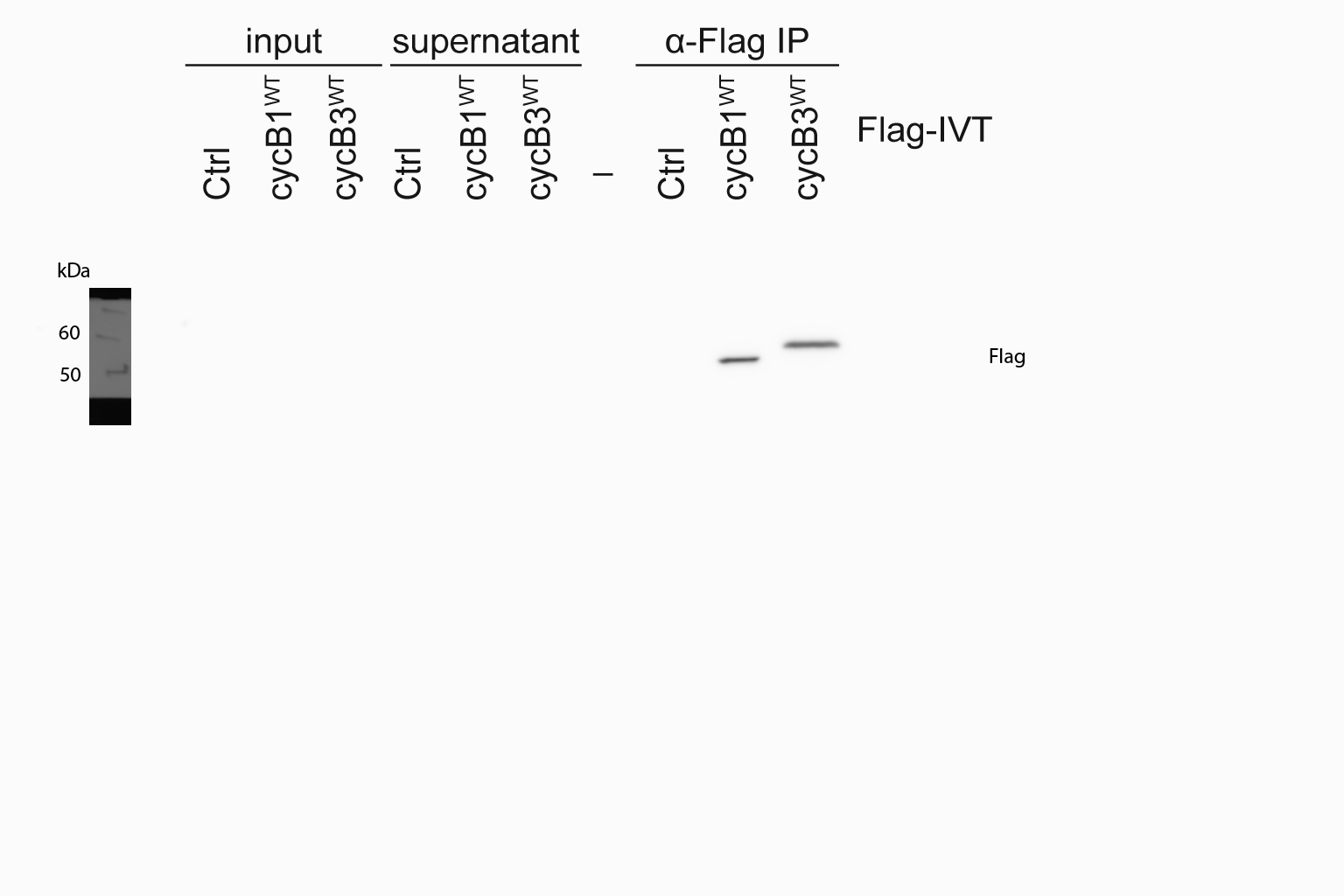

Supplement: Supplementary file 2 — Source data Fig. 1 [file 44319_2024_347_MOESM2_ESM.zip › Figure 1/1C/Western Flag.tif]

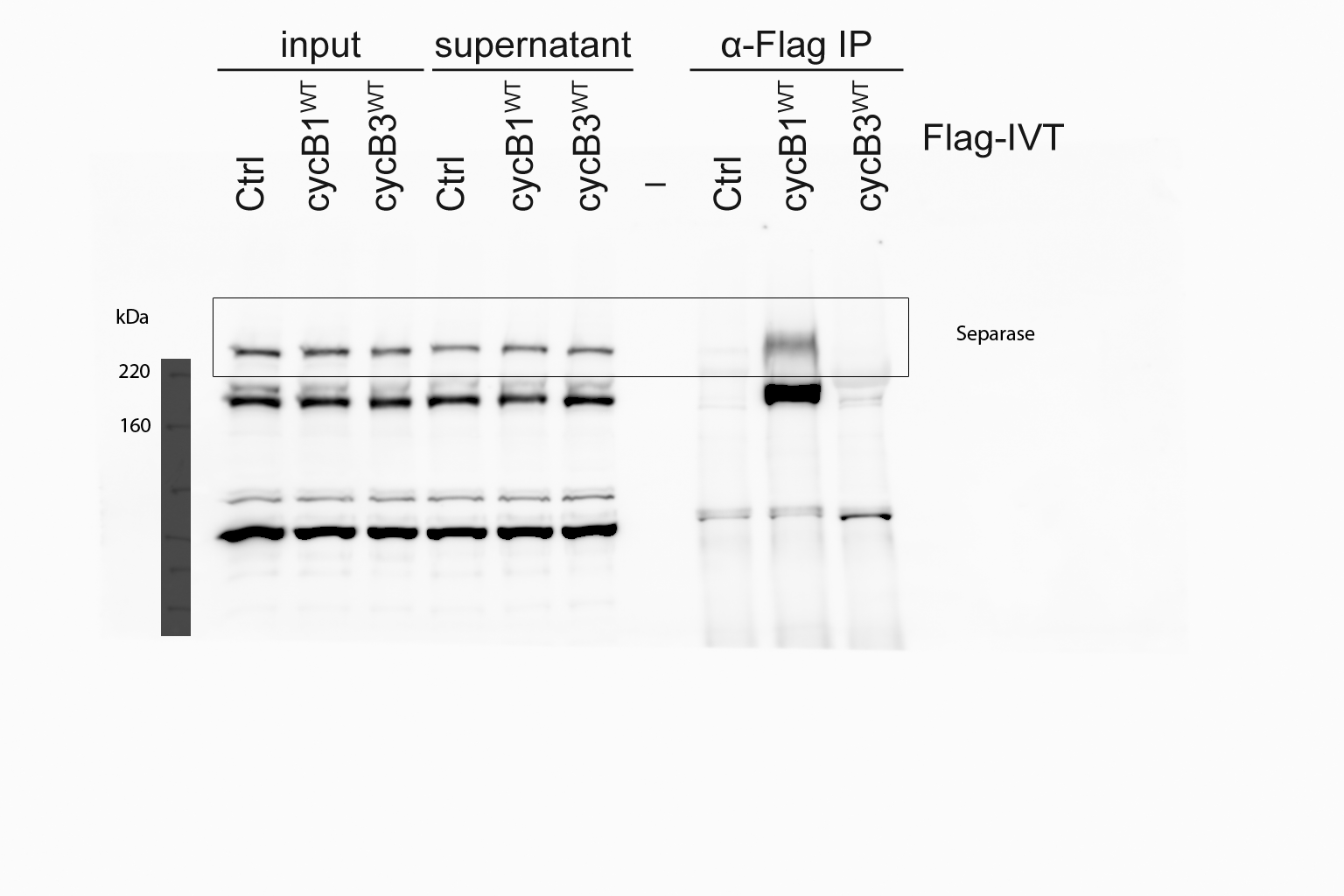

Supplement: Supplementary file 2 — Source data Fig. 1 [file 44319_2024_347_MOESM2_ESM.zip › Figure 1/1C/Western Separase.tif]

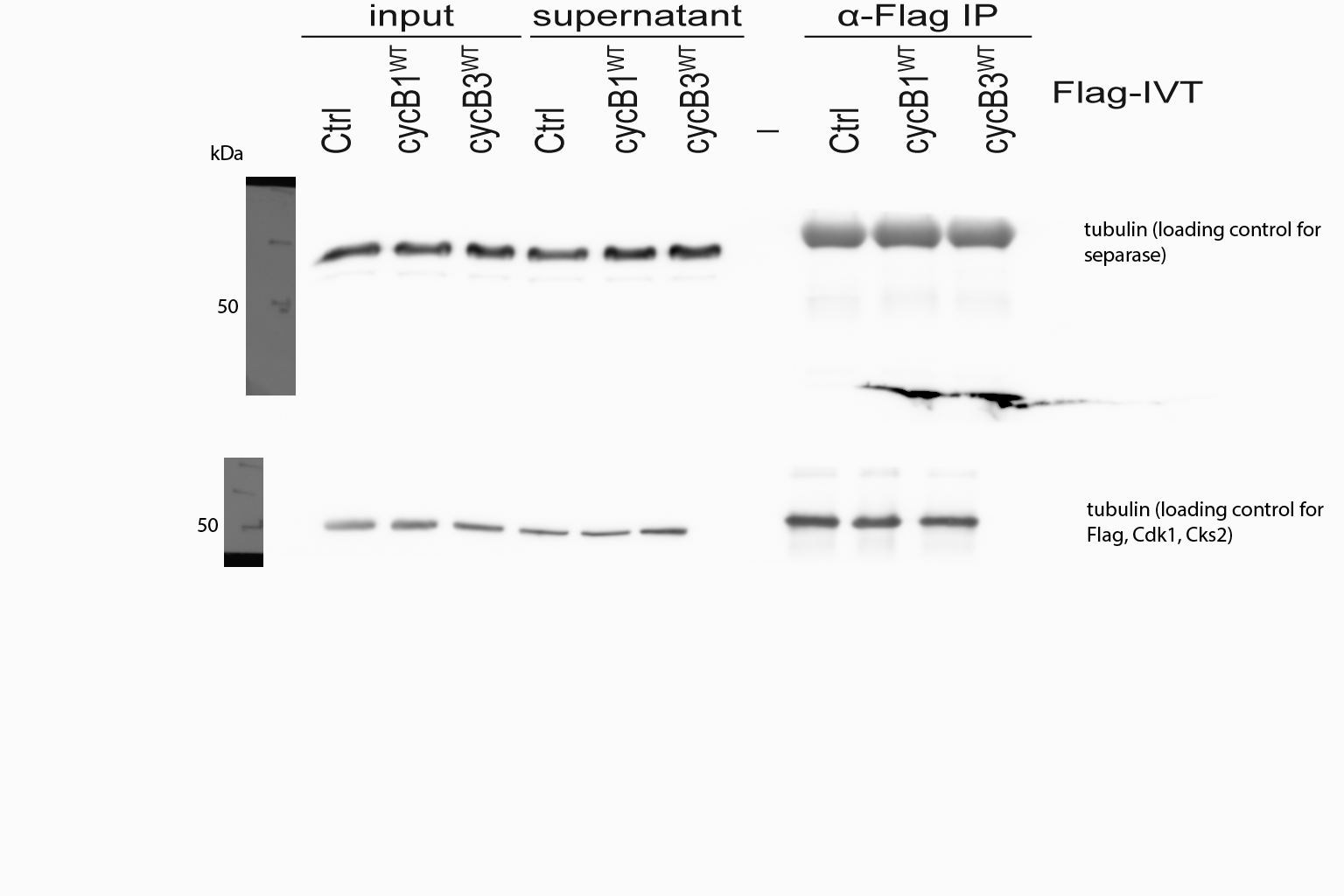

Supplement: Supplementary file 2 — Source data Fig. 1 [file 44319_2024_347_MOESM2_ESM.zip › Figure 1/1C/Western tubulin.tif]

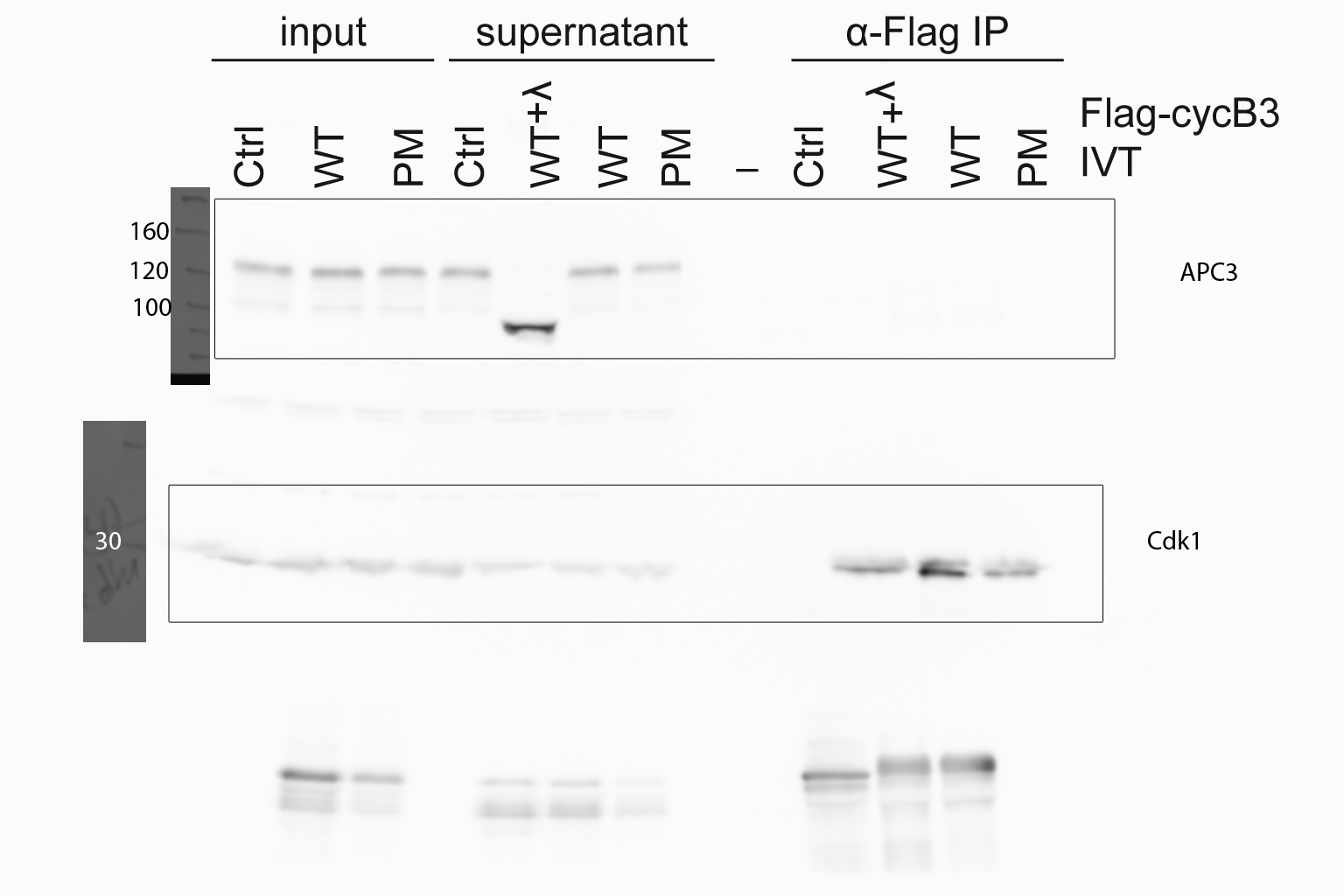

Supplement: Supplementary file 2 — Source data Fig. 1 [file 44319_2024_347_MOESM2_ESM.zip › Figure 1/1D/Western APC3 and Cdk1.tif]

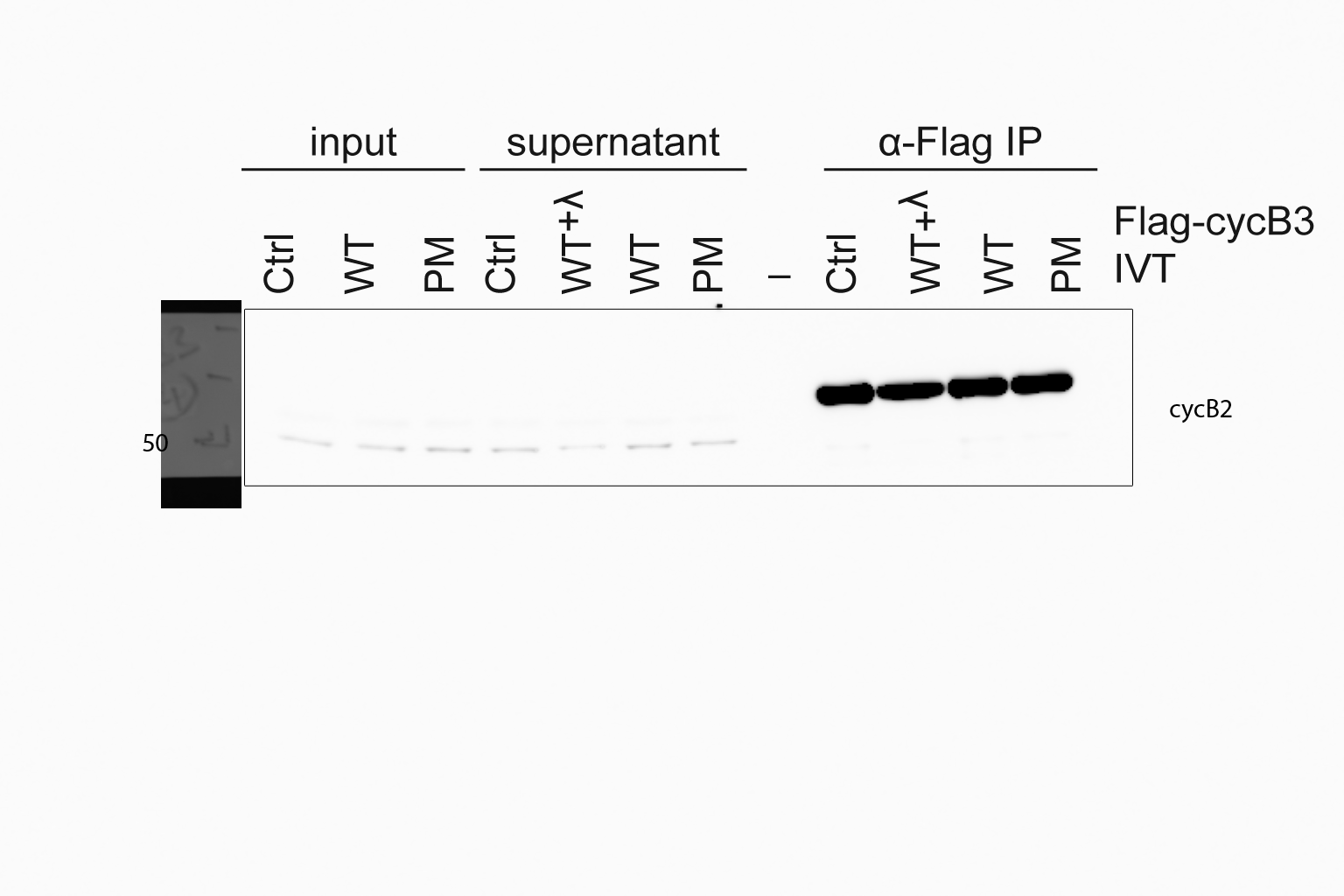

Supplement: Supplementary file 2 — Source data Fig. 1 [file 44319_2024_347_MOESM2_ESM.zip › Figure 1/1D/Western cycB2.tif]

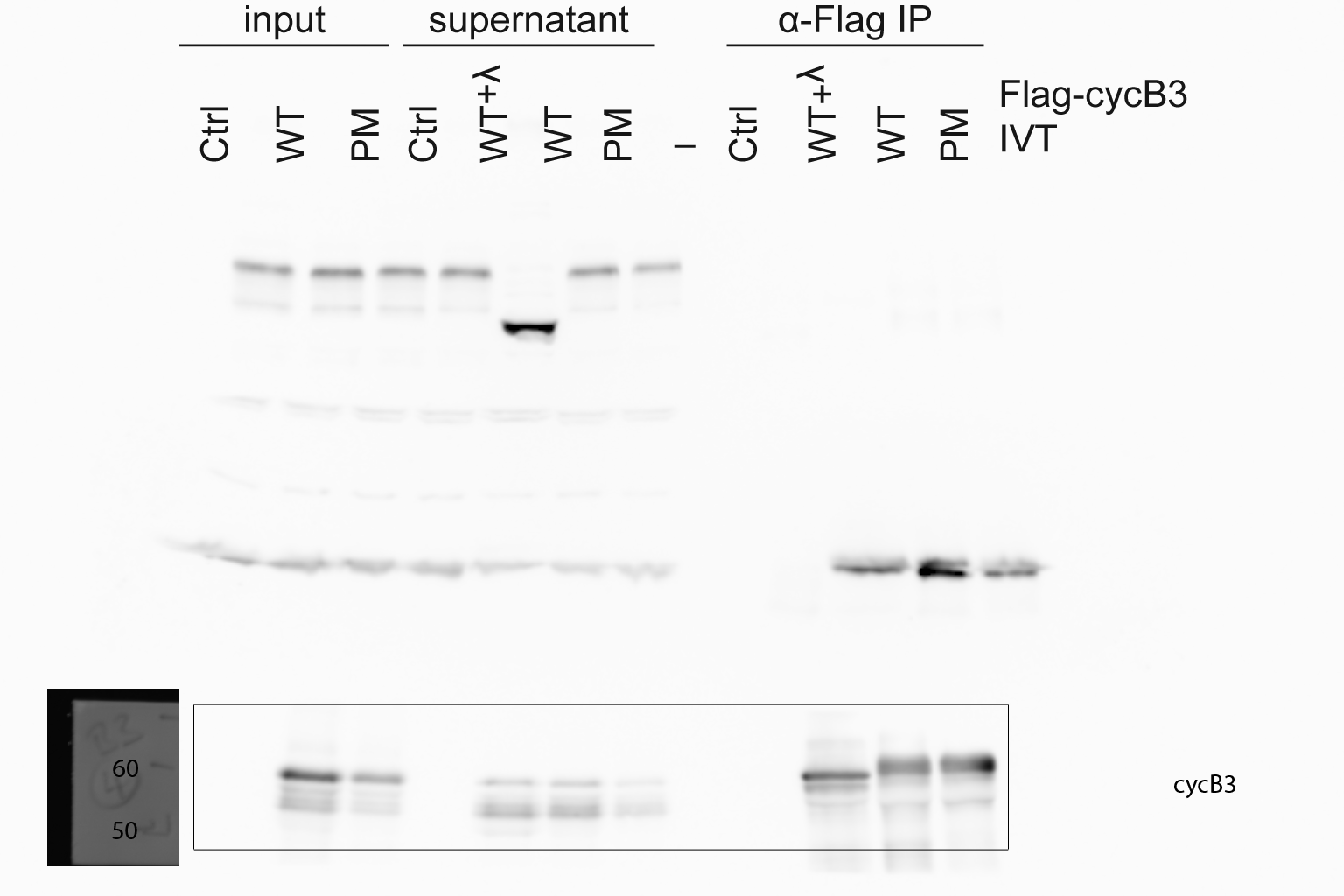

Supplement: Supplementary file 2 — Source data Fig. 1 [file 44319_2024_347_MOESM2_ESM.zip › Figure 1/1D/Western cycB3.tif]

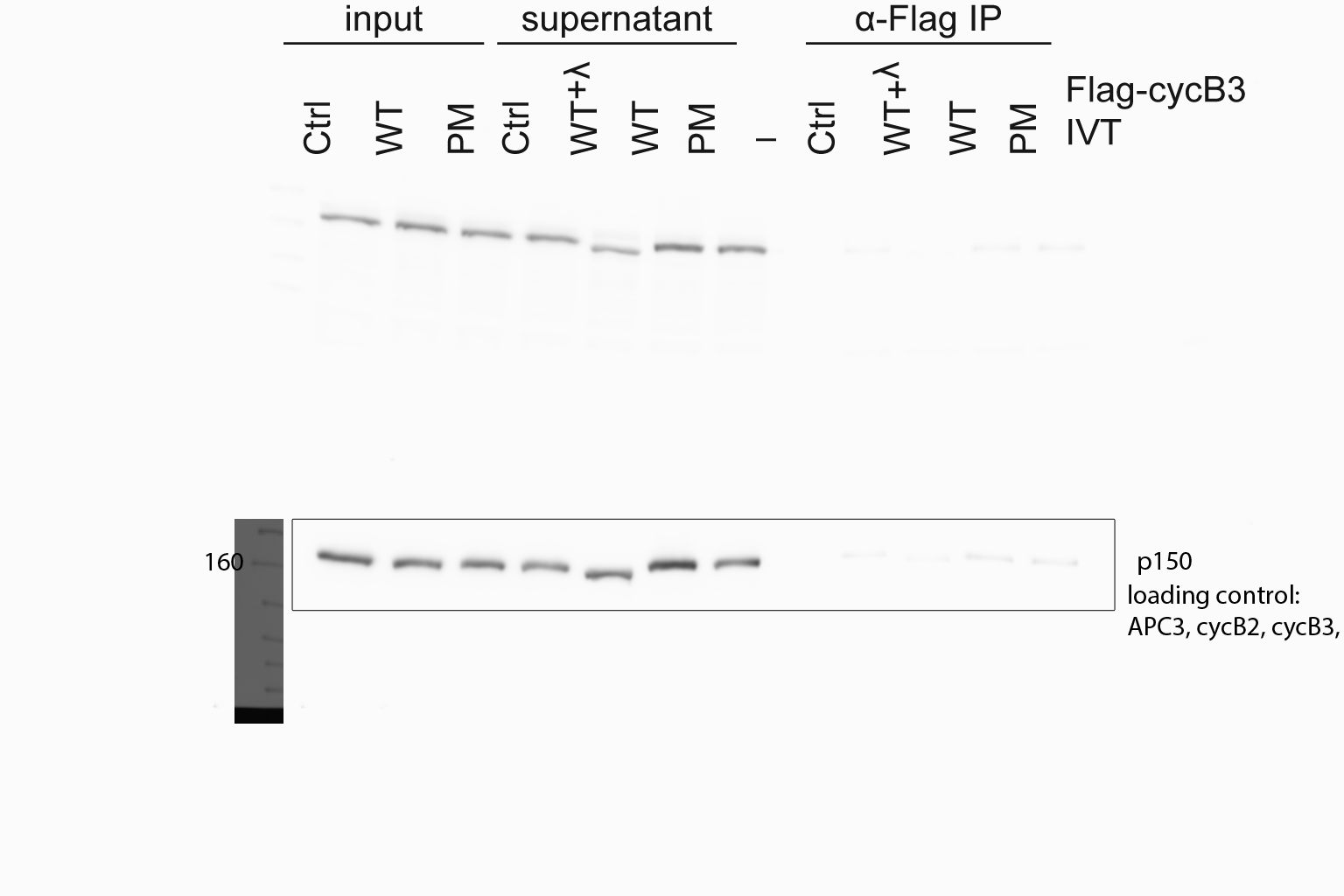

Supplement: Supplementary file 2 — Source data Fig. 1 [file 44319_2024_347_MOESM2_ESM.zip › Figure 1/1D/Western p150.tif]

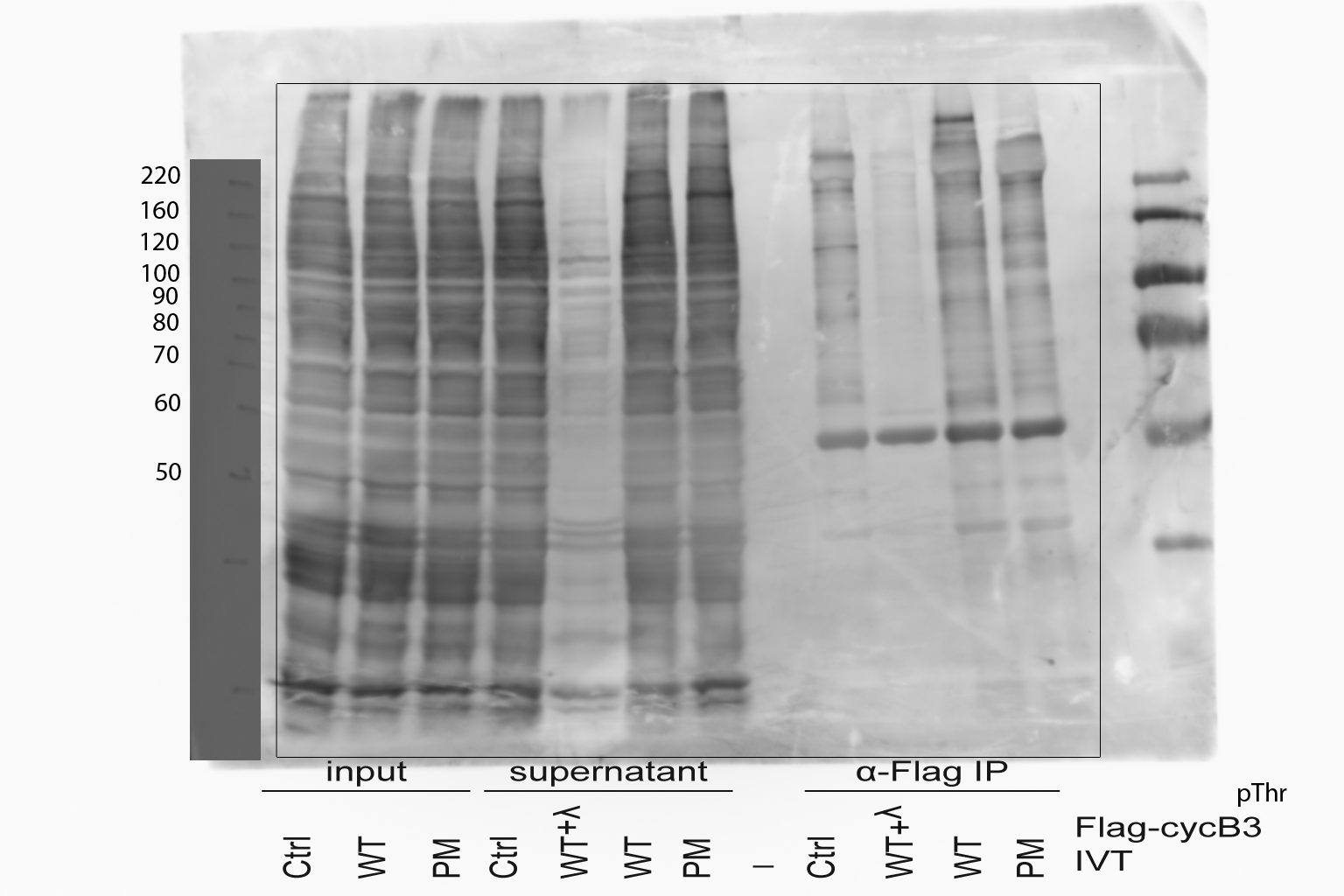

Supplement: Supplementary file 2 — Source data Fig. 1 [file 44319_2024_347_MOESM2_ESM.zip › Figure 1/1D/Western pThr.tif]

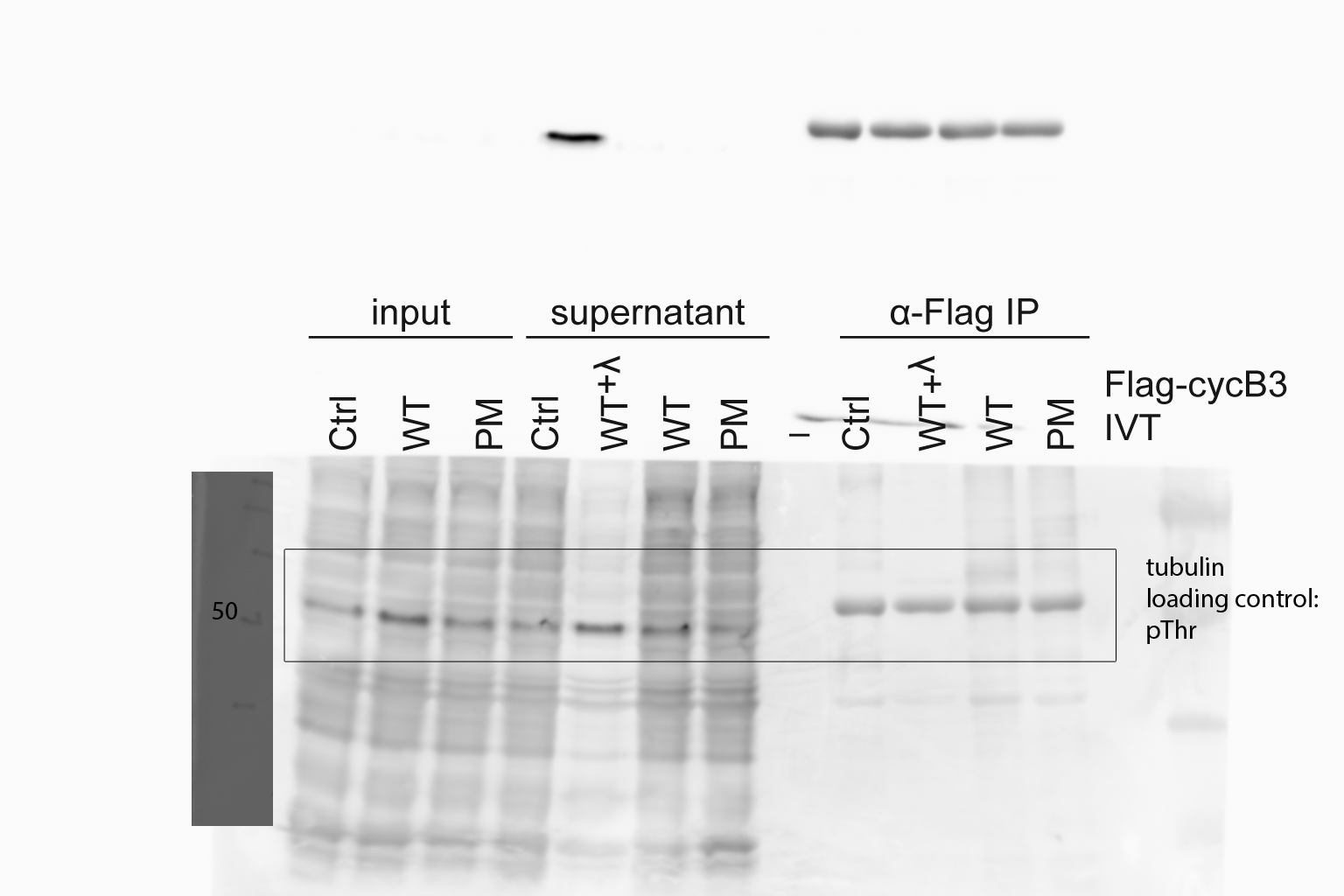

Supplement: Supplementary file 2 — Source data Fig. 1 [file 44319_2024_347_MOESM2_ESM.zip › Figure 1/1D/Western tubulin.tif]

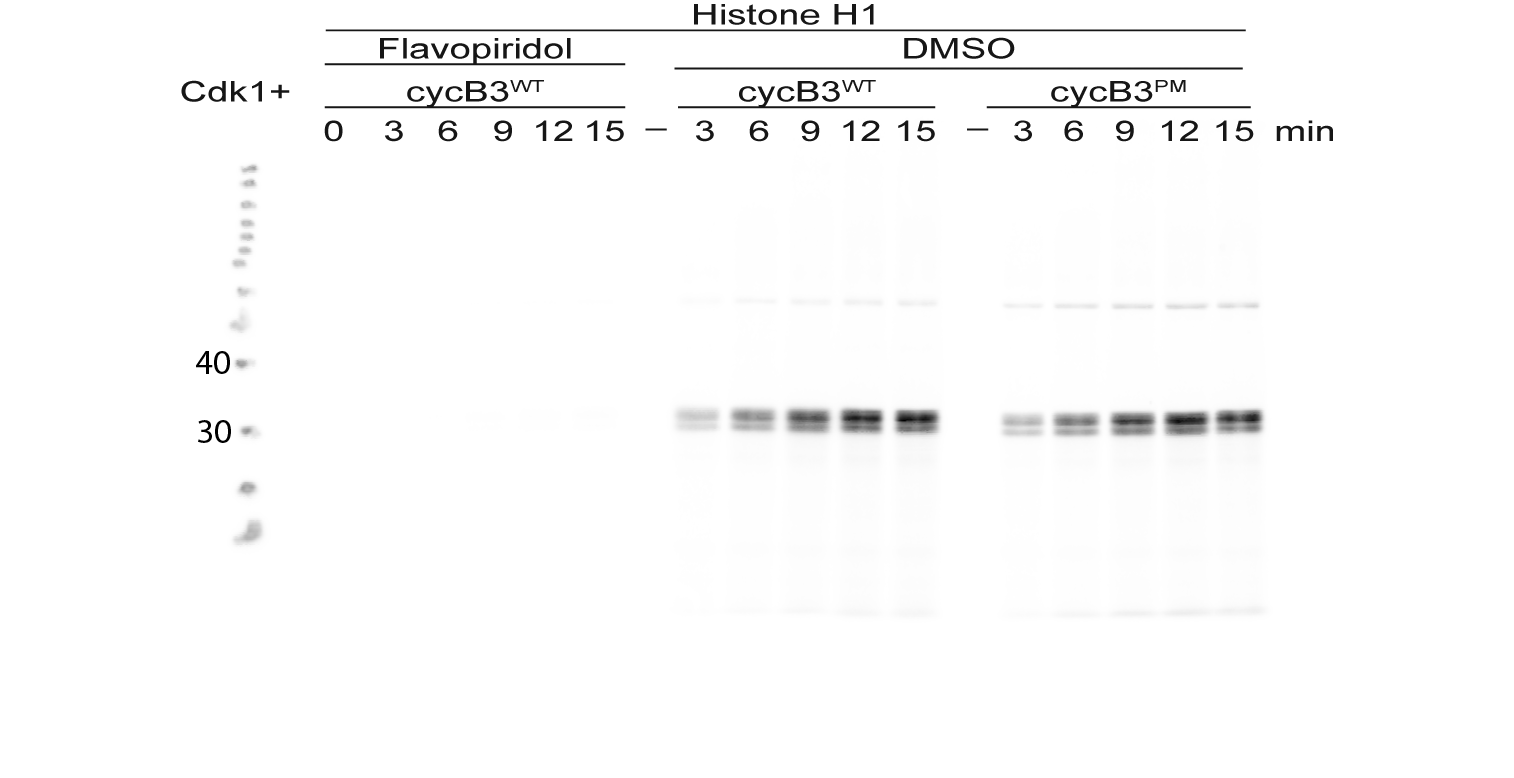

Supplement: Supplementary file 2 — Source data Fig. 1 [file 44319_2024_347_MOESM2_ESM.zip › Figure 1/1E/Autoradiogram P33.tif]

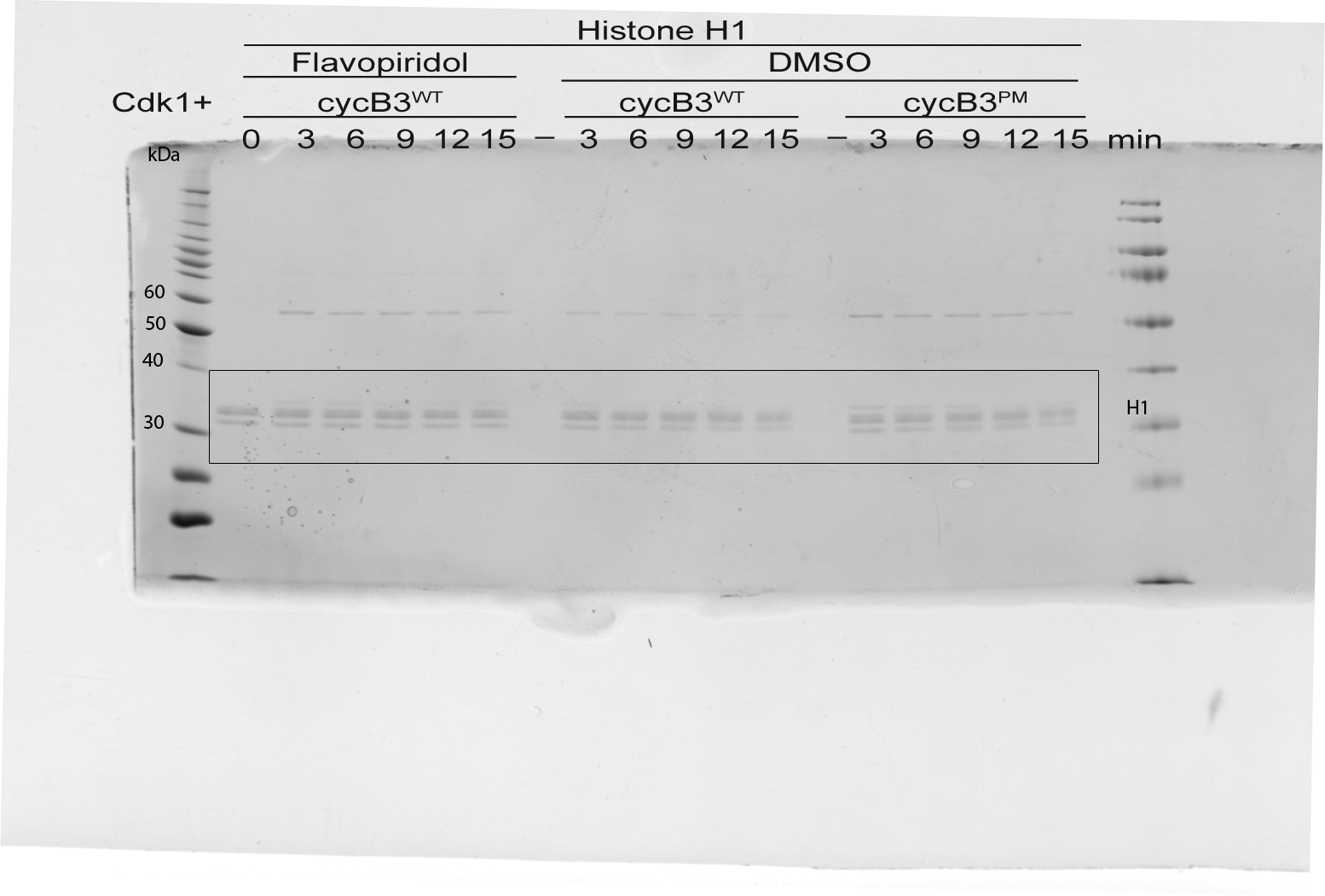

Supplement: Supplementary file 2 — Source data Fig. 1 [file 44319_2024_347_MOESM2_ESM.zip › Figure 1/1E/Coomassie H1.tif]

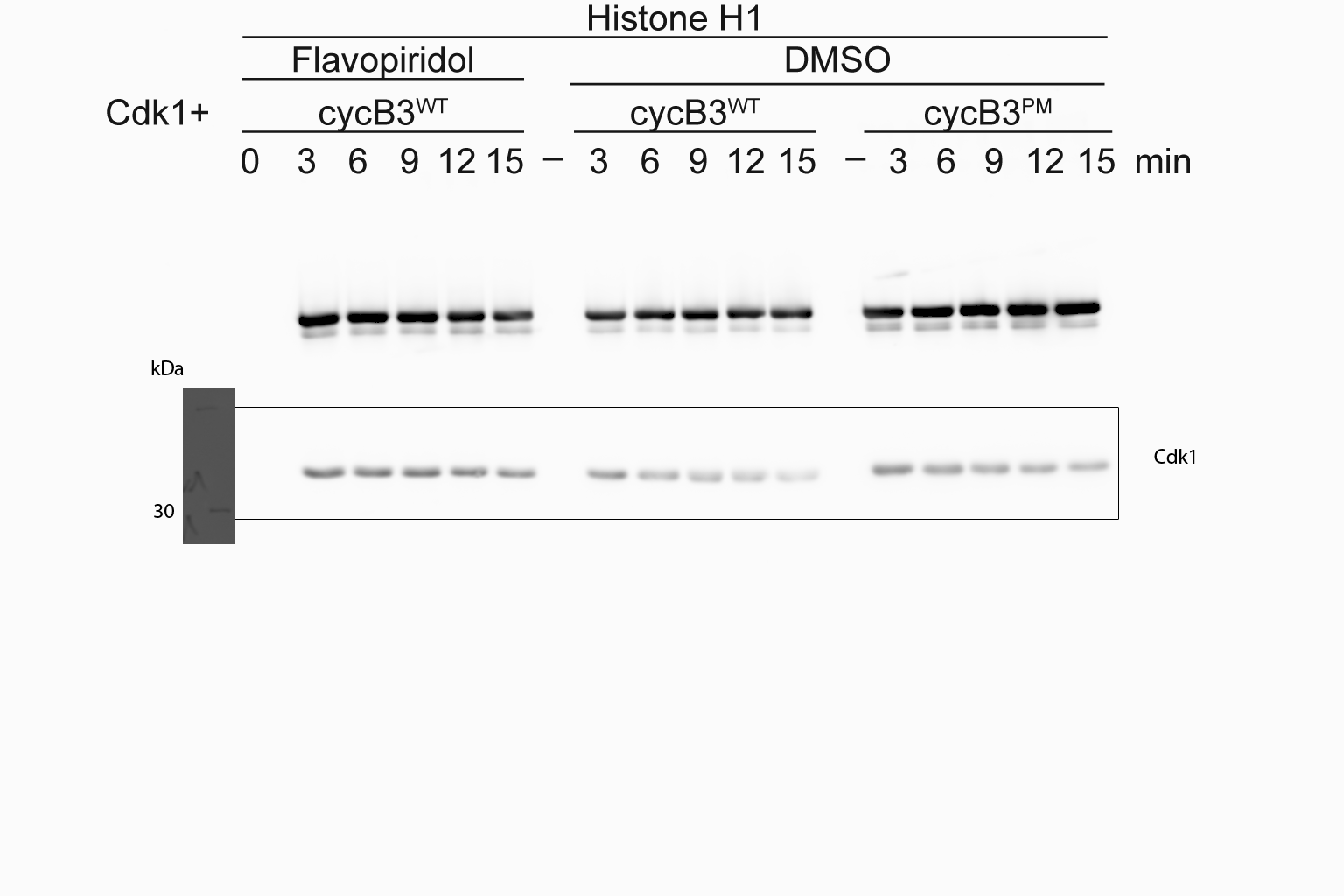

Supplement: Supplementary file 2 — Source data Fig. 1 [file 44319_2024_347_MOESM2_ESM.zip › Figure 1/1E/Western Cdk1.tif]

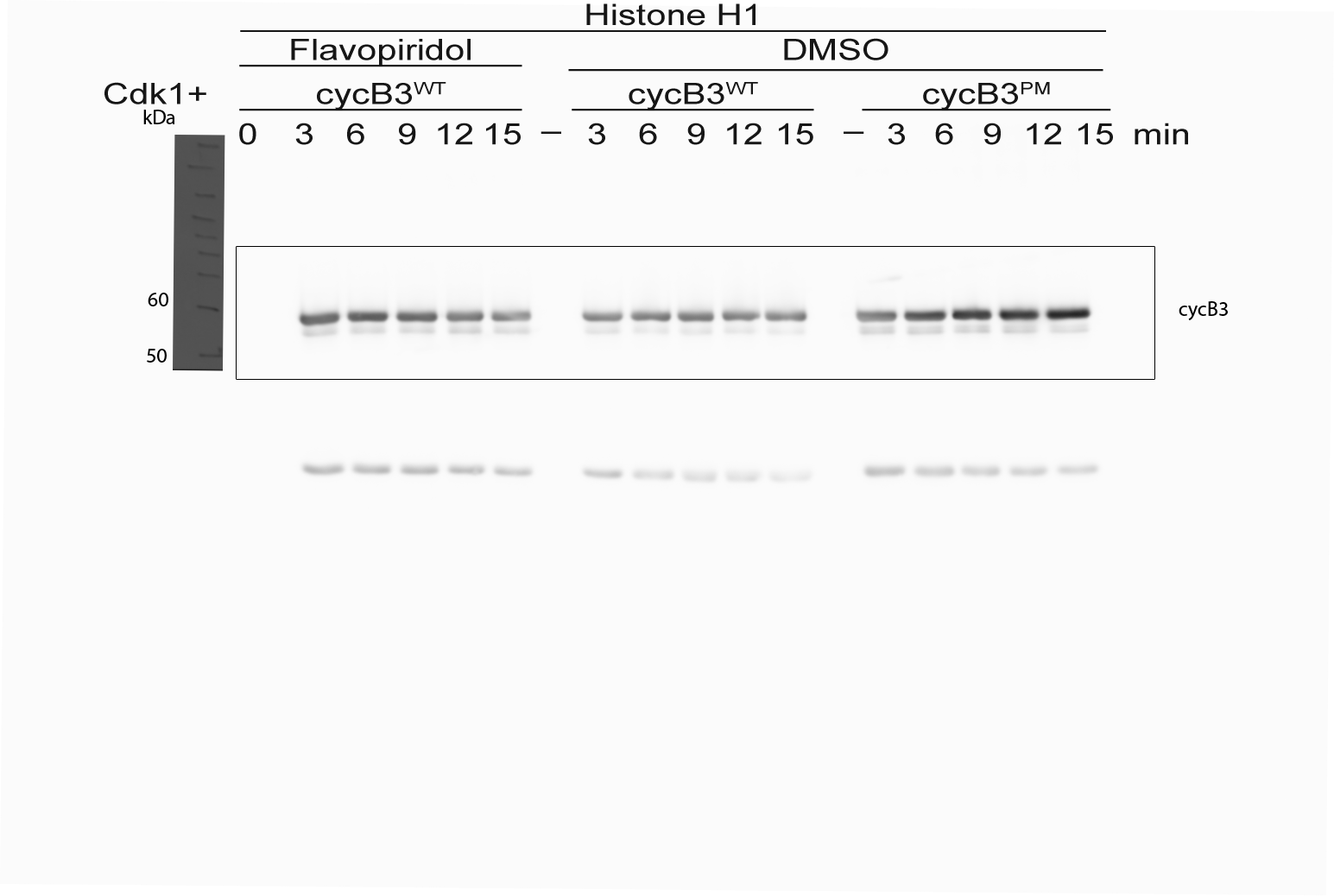

Supplement: Supplementary file 2 — Source data Fig. 1 [file 44319_2024_347_MOESM2_ESM.zip › Figure 1/1E/Western cycB3.tif]

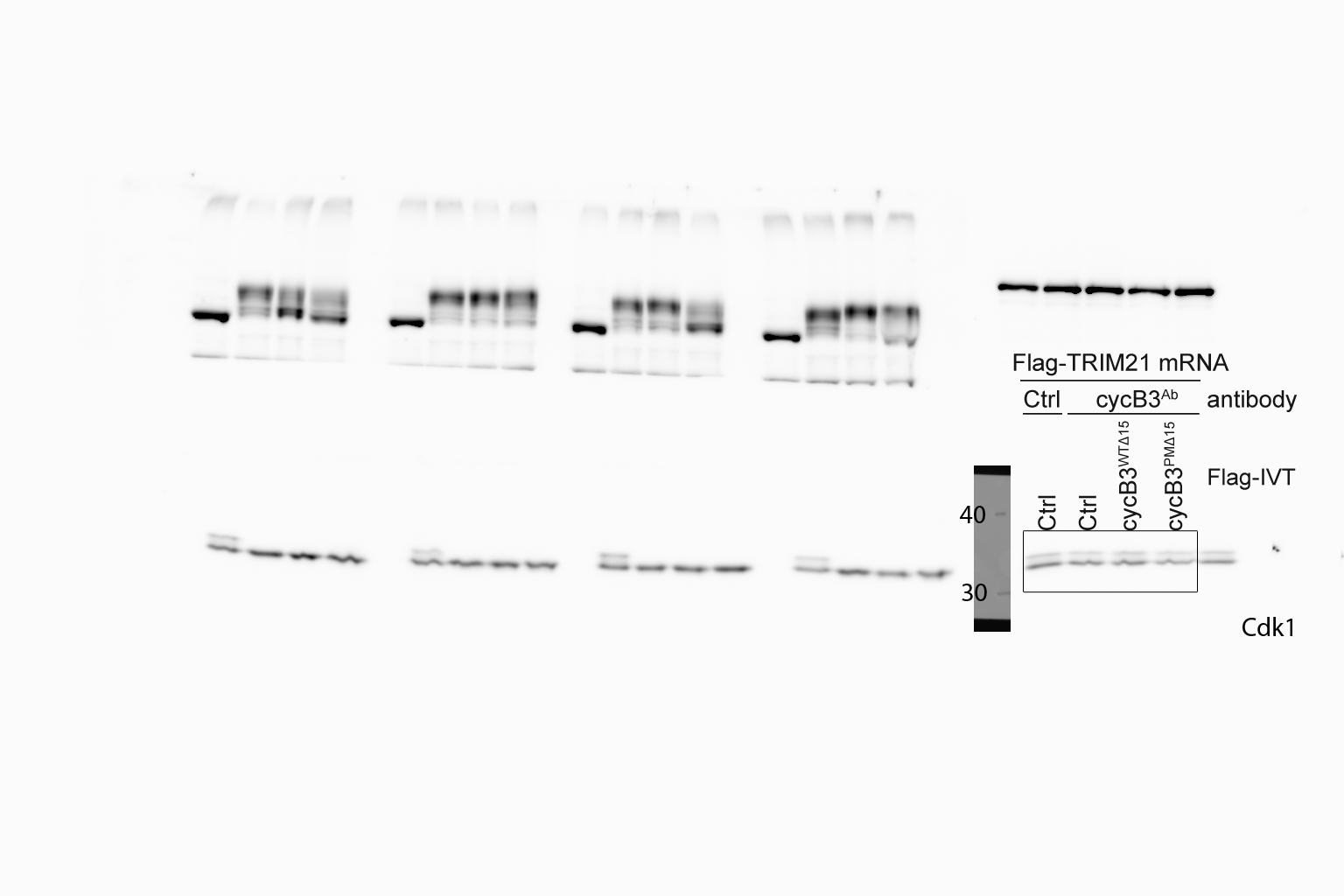

Supplement: Supplementary file 3 — Source data Fig. 2 [file 44319_2024_347_MOESM3_ESM.zip › Figure 2/2B/Western Cdk1.tif]

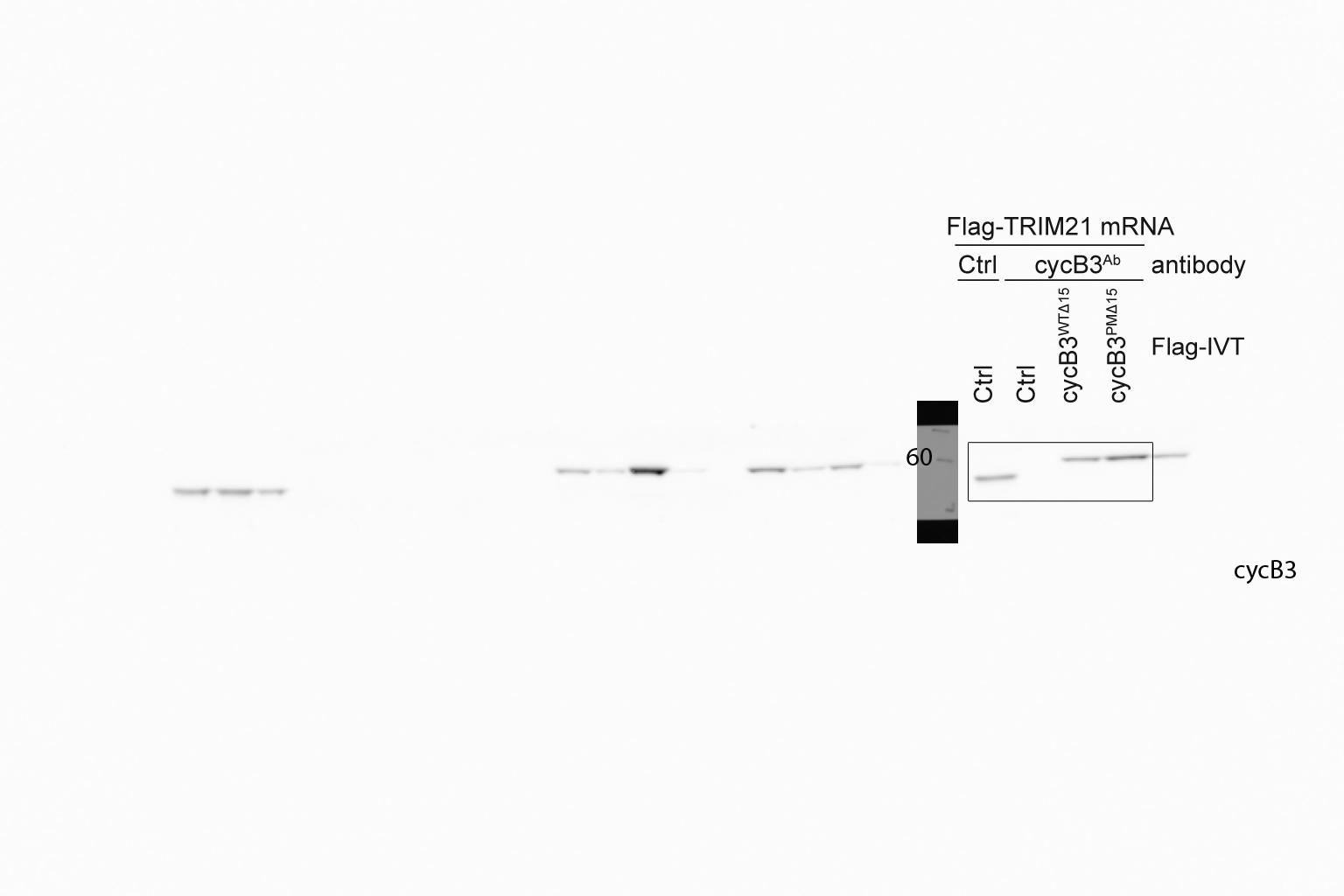

Supplement: Supplementary file 3 — Source data Fig. 2 [file 44319_2024_347_MOESM3_ESM.zip › Figure 2/2B/Western cycB3.tif]

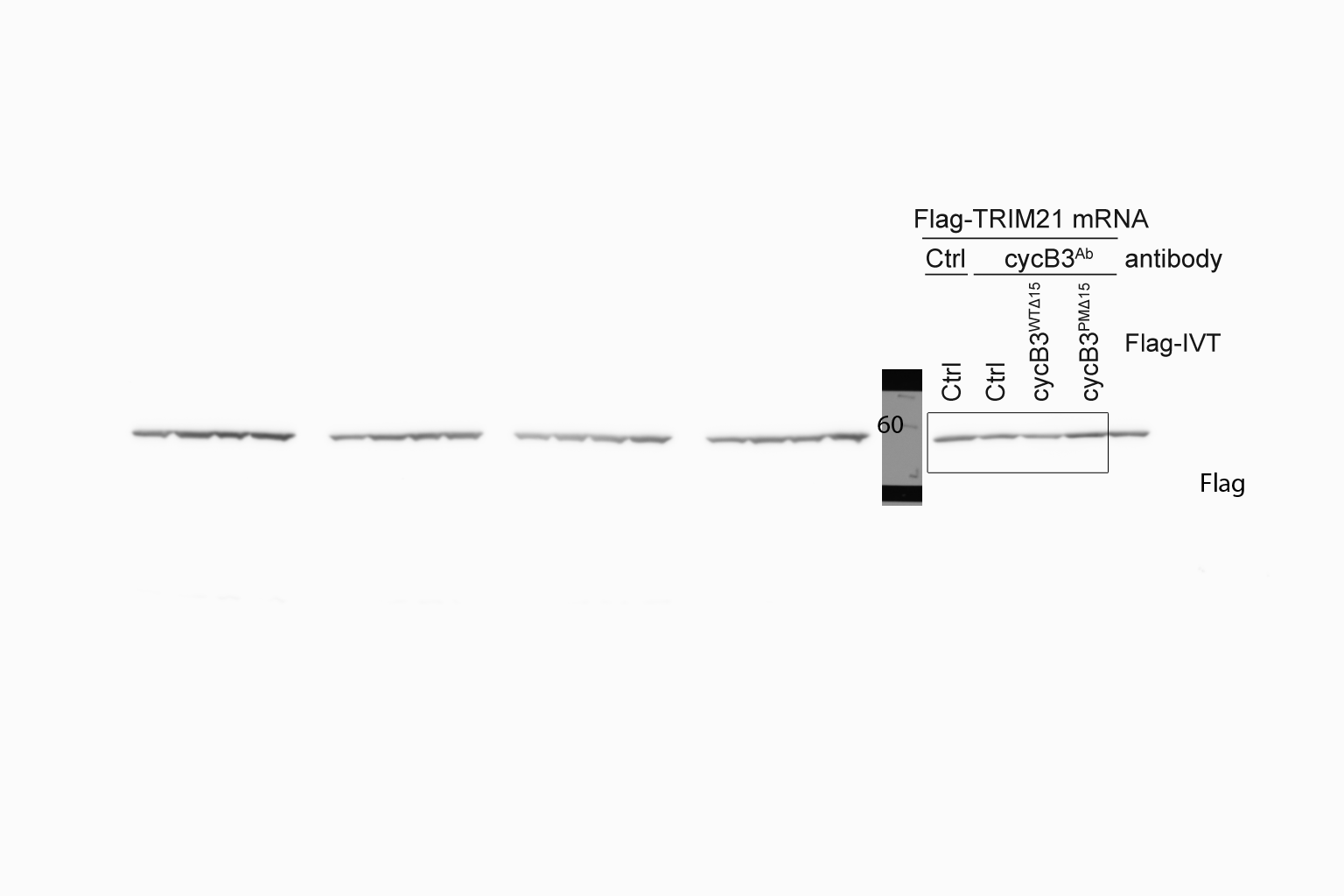

Supplement: Supplementary file 3 — Source data Fig. 2 [file 44319_2024_347_MOESM3_ESM.zip › Figure 2/2B/Western Flag.tif]

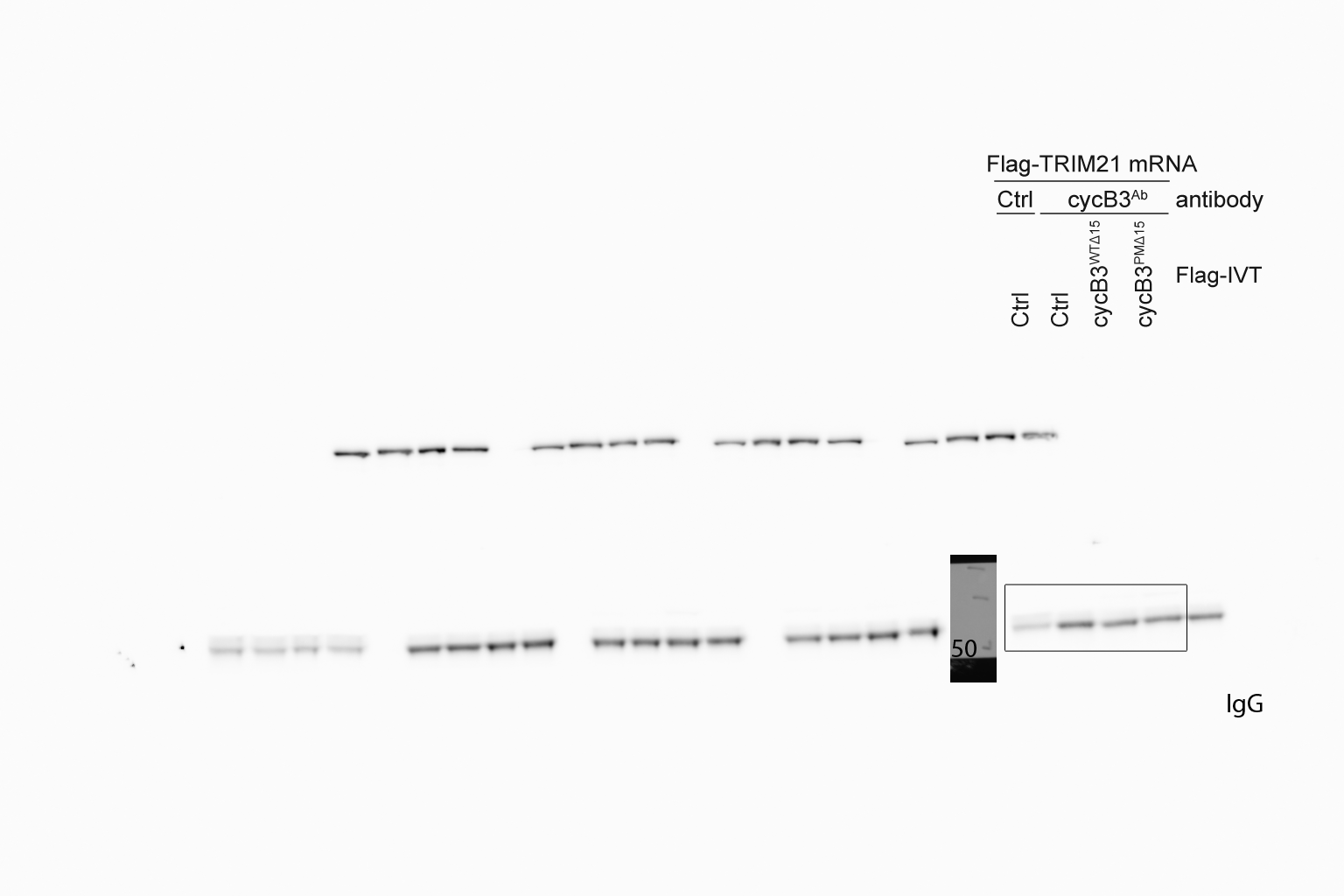

Supplement: Supplementary file 3 — Source data Fig. 2 [file 44319_2024_347_MOESM3_ESM.zip › Figure 2/2B/Western IgG.tif]

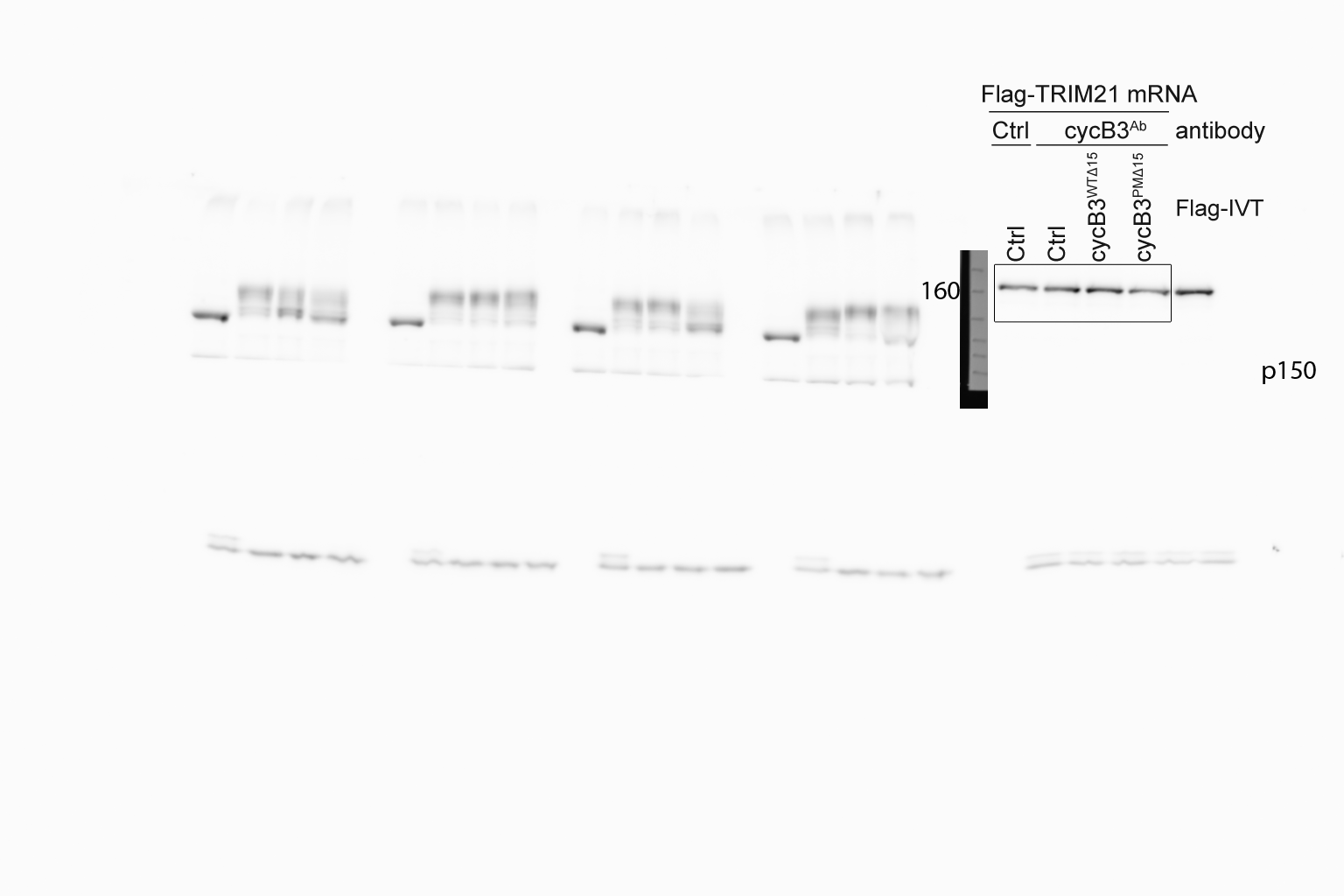

Supplement: Supplementary file 3 — Source data Fig. 2 [file 44319_2024_347_MOESM3_ESM.zip › Figure 2/2B/Western p150.tif]

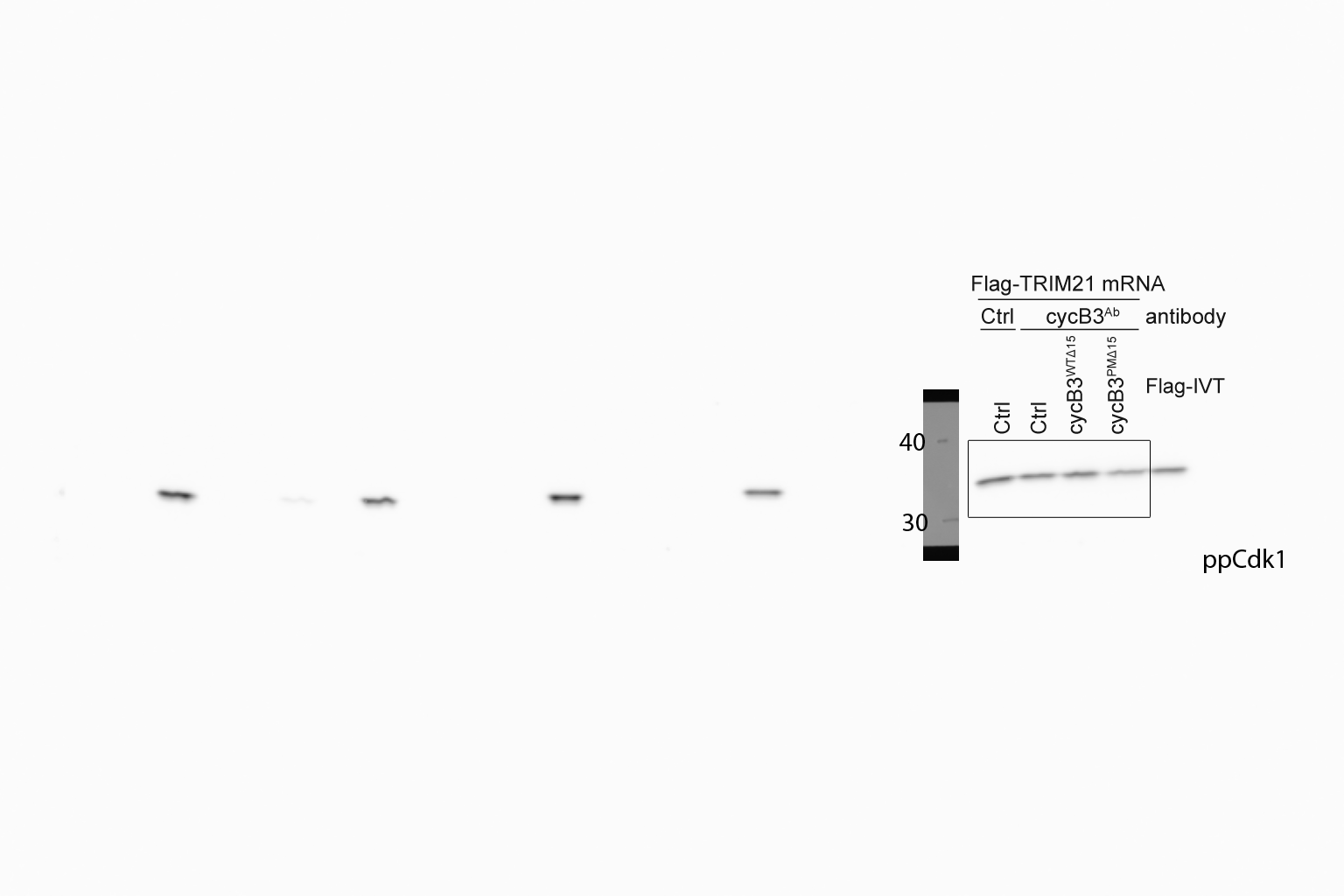

Supplement: Supplementary file 3 — Source data Fig. 2 [file 44319_2024_347_MOESM3_ESM.zip › Figure 2/2B/Western ppCdk1.tif]

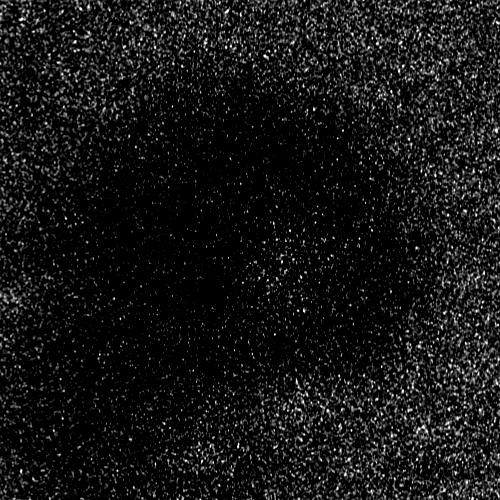

Supplement: Supplementary file 3 — Source data Fig. 2 [file 44319_2024_347_MOESM3_ESM.zip › Figure 2/2C/Anaphase I.tif]

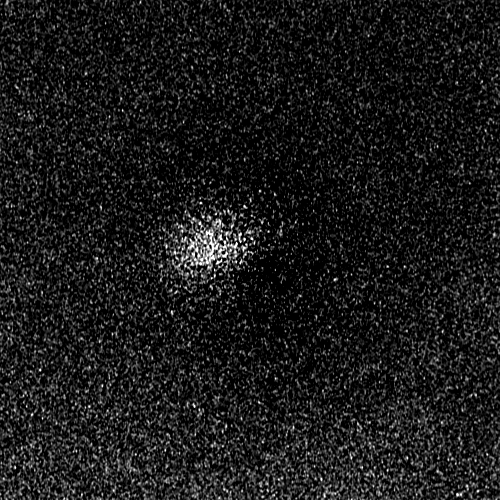

Supplement: Supplementary file 3 — Source data Fig. 2 [file 44319_2024_347_MOESM3_ESM.zip › Figure 2/2C/MI spindle.tif]

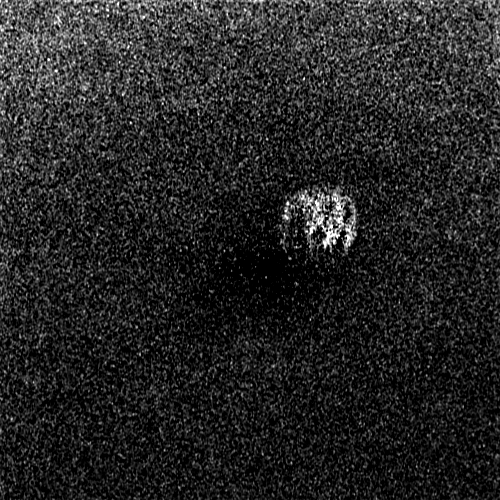

Supplement: Supplementary file 3 — Source data Fig. 2 [file 44319_2024_347_MOESM3_ESM.zip › Figure 2/2C/MII spindle and PB_1.tif]

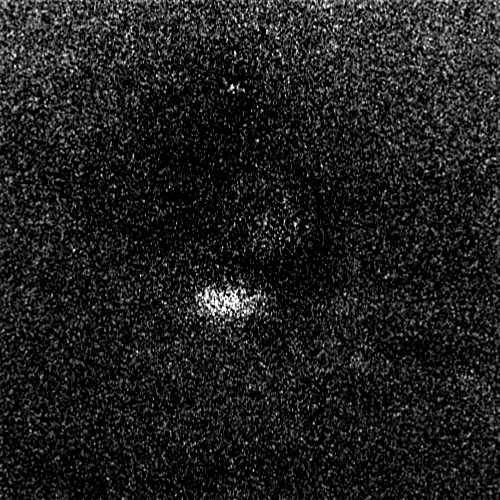

Supplement: Supplementary file 3 — Source data Fig. 2 [file 44319_2024_347_MOESM3_ESM.zip › Figure 2/2C/MII spindle and PB_2.tif]

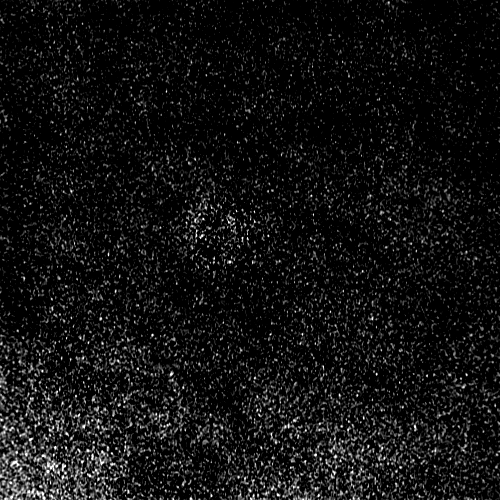

Supplement: Supplementary file 3 — Source data Fig. 2 [file 44319_2024_347_MOESM3_ESM.zip › Figure 2/2C/PB.tif]

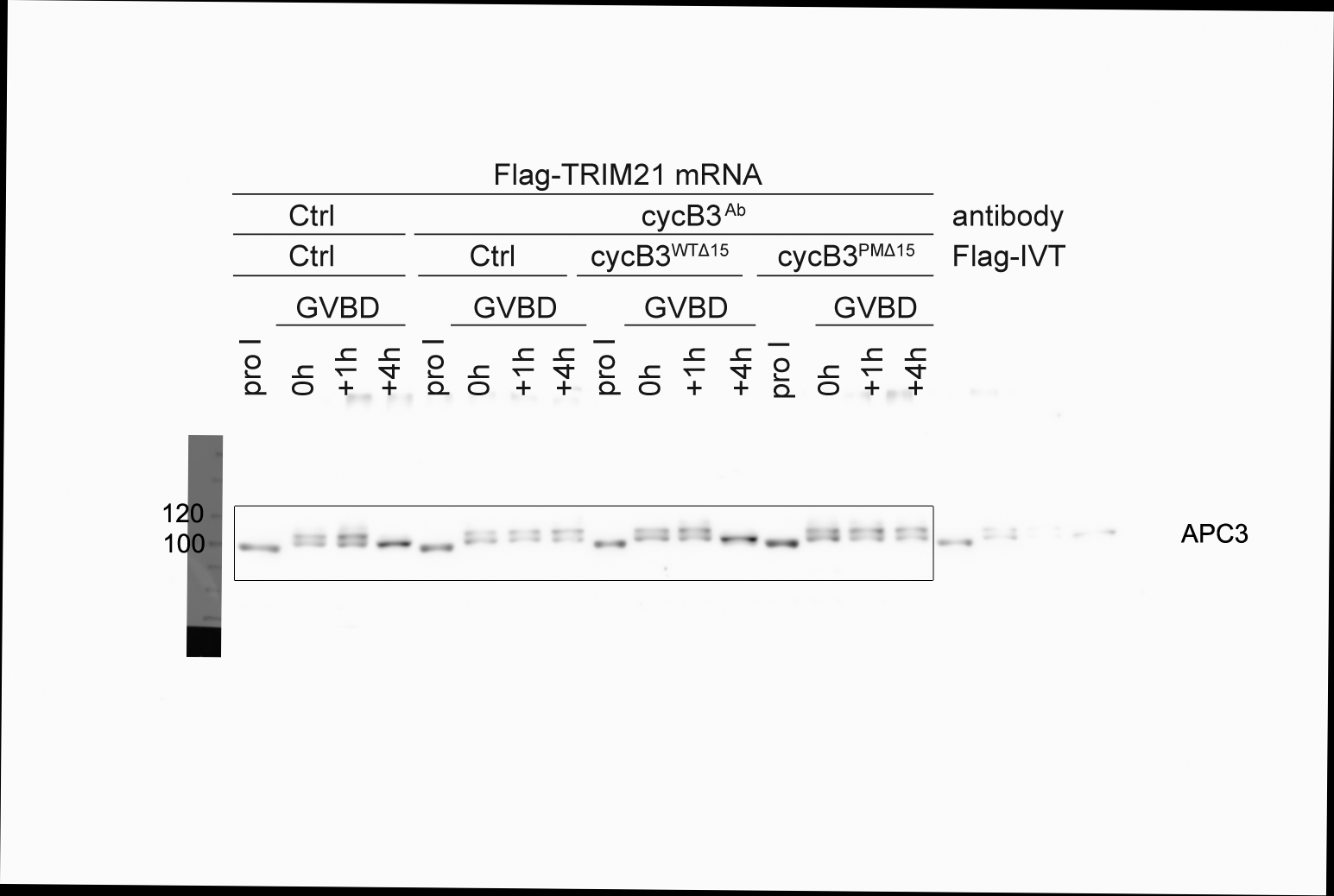

Supplement: Supplementary file 3 — Source data Fig. 2 [file 44319_2024_347_MOESM3_ESM.zip › Figure 2/2E/Western APC3.tif]

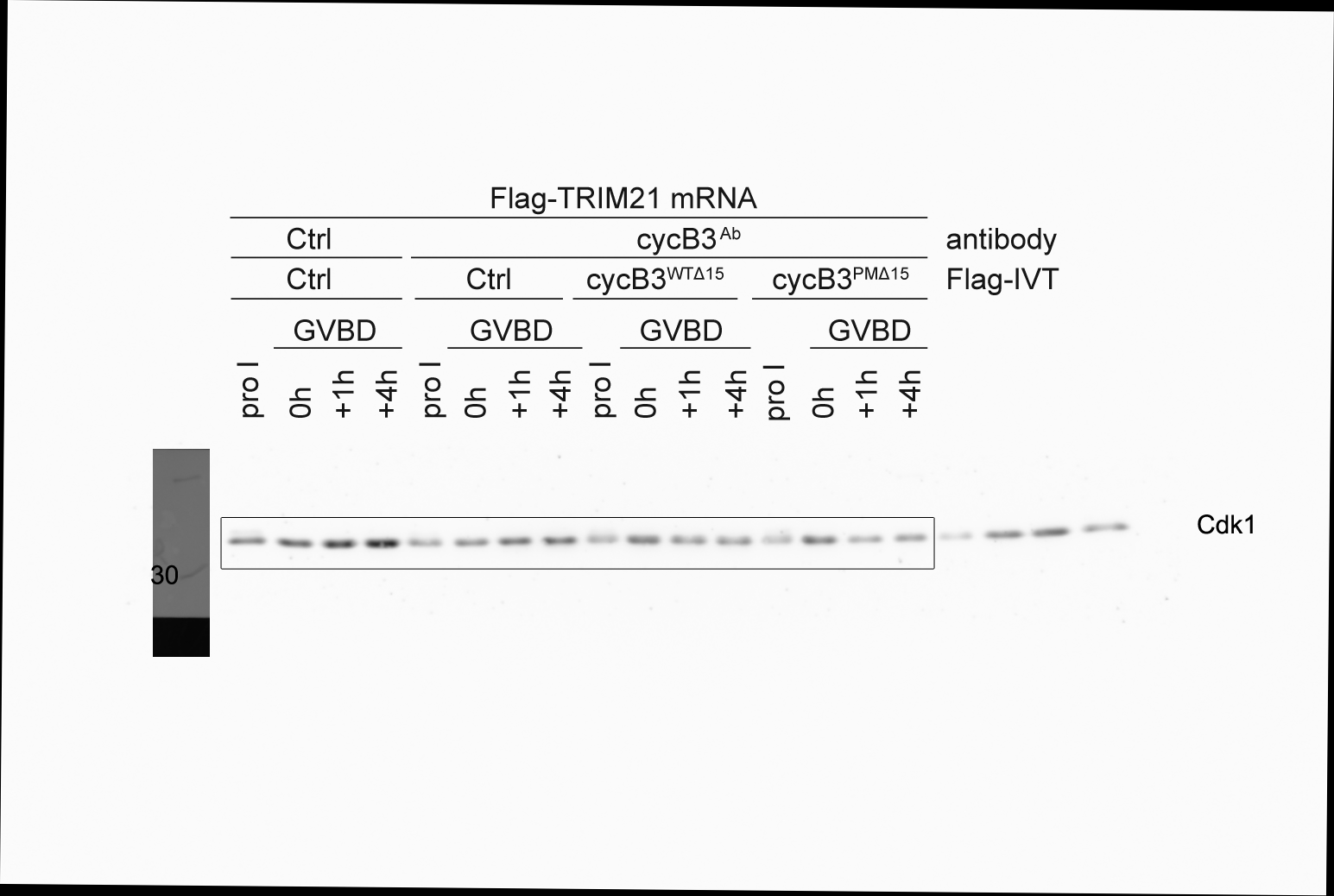

Supplement: Supplementary file 3 — Source data Fig. 2 [file 44319_2024_347_MOESM3_ESM.zip › Figure 2/2E/Western Cdk1.tif]

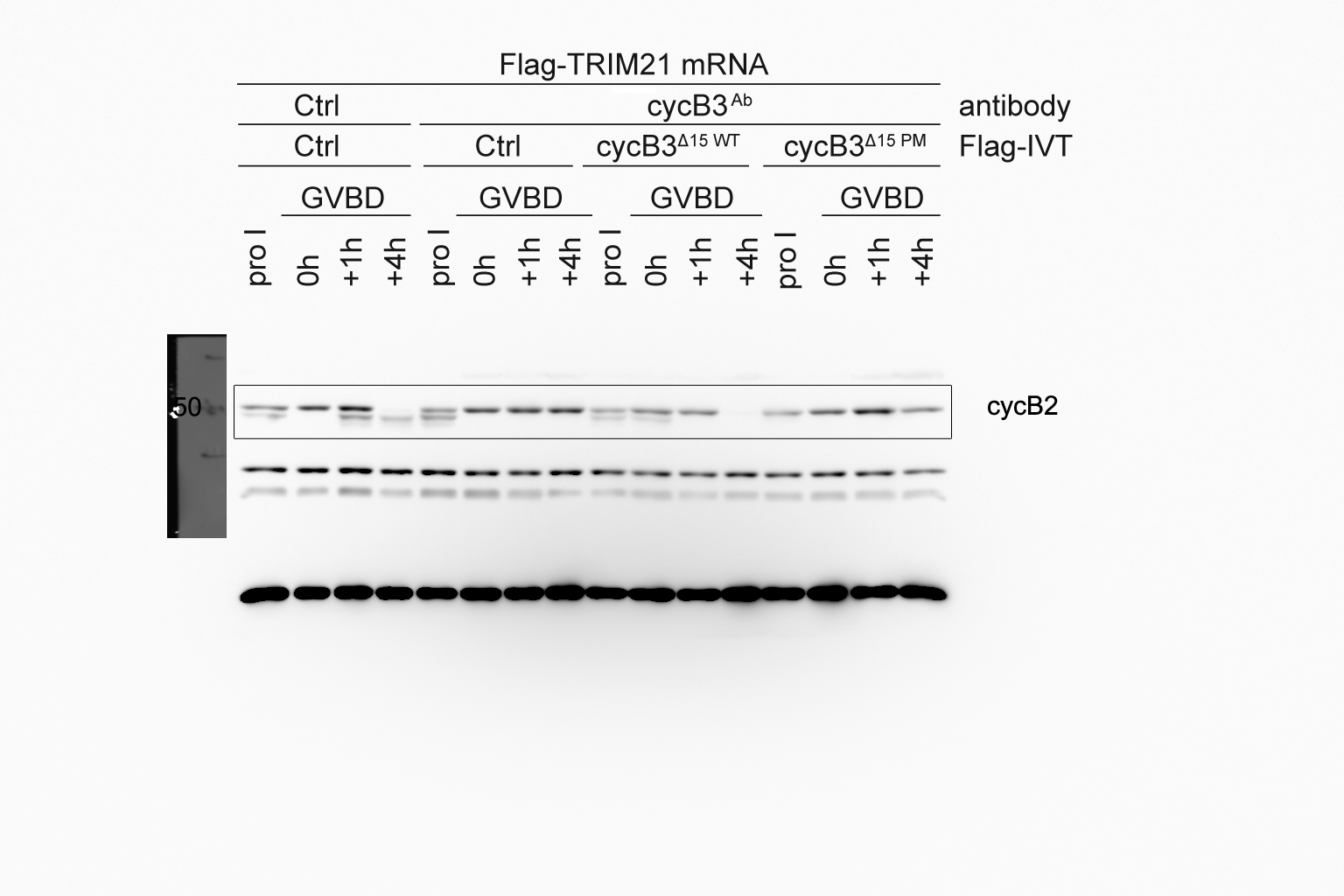

Supplement: Supplementary file 3 — Source data Fig. 2 [file 44319_2024_347_MOESM3_ESM.zip › Figure 2/2E/Western cycB2.tif]

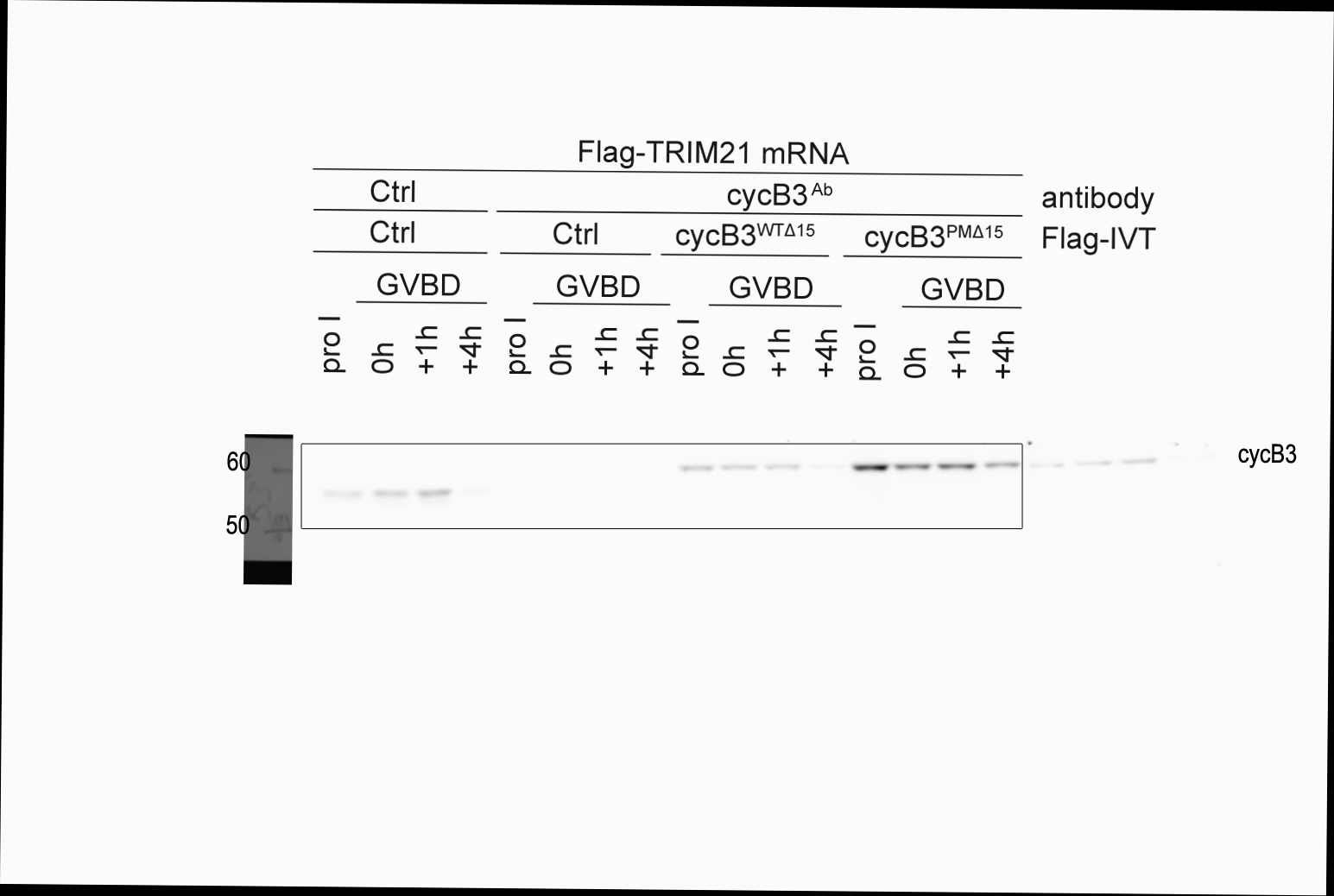

Supplement: Supplementary file 3 — Source data Fig. 2 [file 44319_2024_347_MOESM3_ESM.zip › Figure 2/2E/Western cycB3.tif]

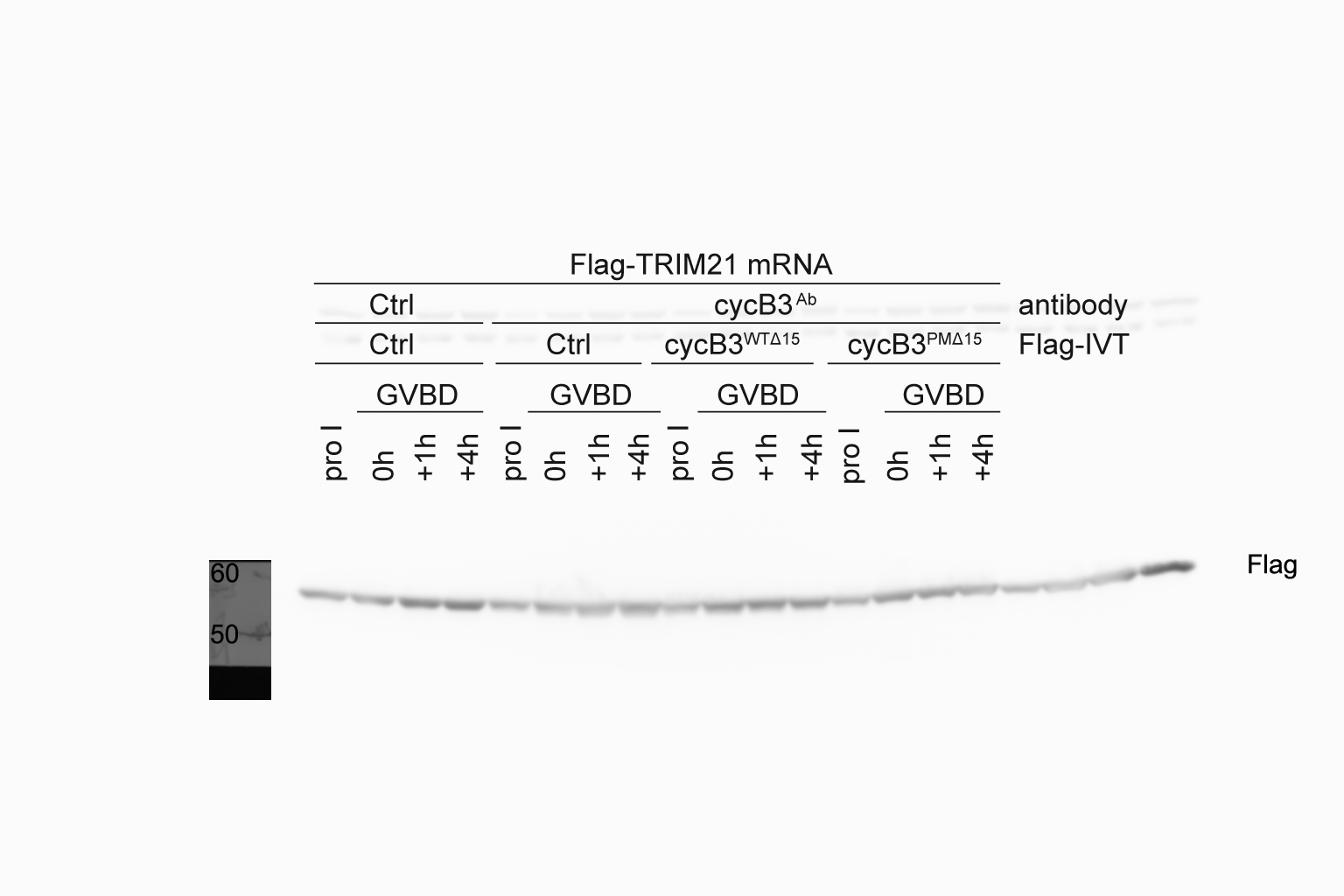

Supplement: Supplementary file 3 — Source data Fig. 2 [file 44319_2024_347_MOESM3_ESM.zip › Figure 2/2E/Western Flag.tif]

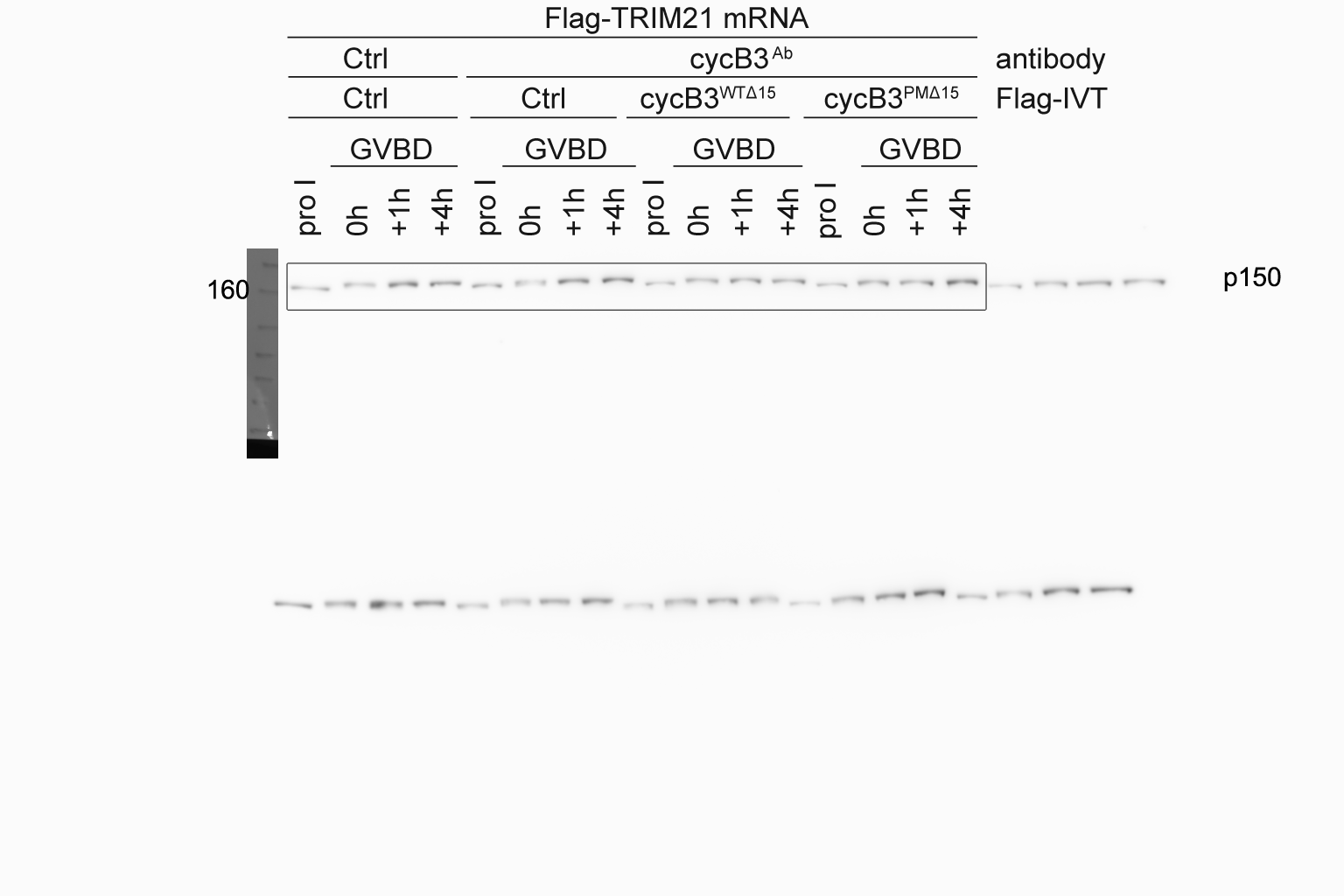

Supplement: Supplementary file 3 — Source data Fig. 2 [file 44319_2024_347_MOESM3_ESM.zip › Figure 2/2E/Western p150 (loading control APC3, cycB3, ppCdk1, Flag).tif]

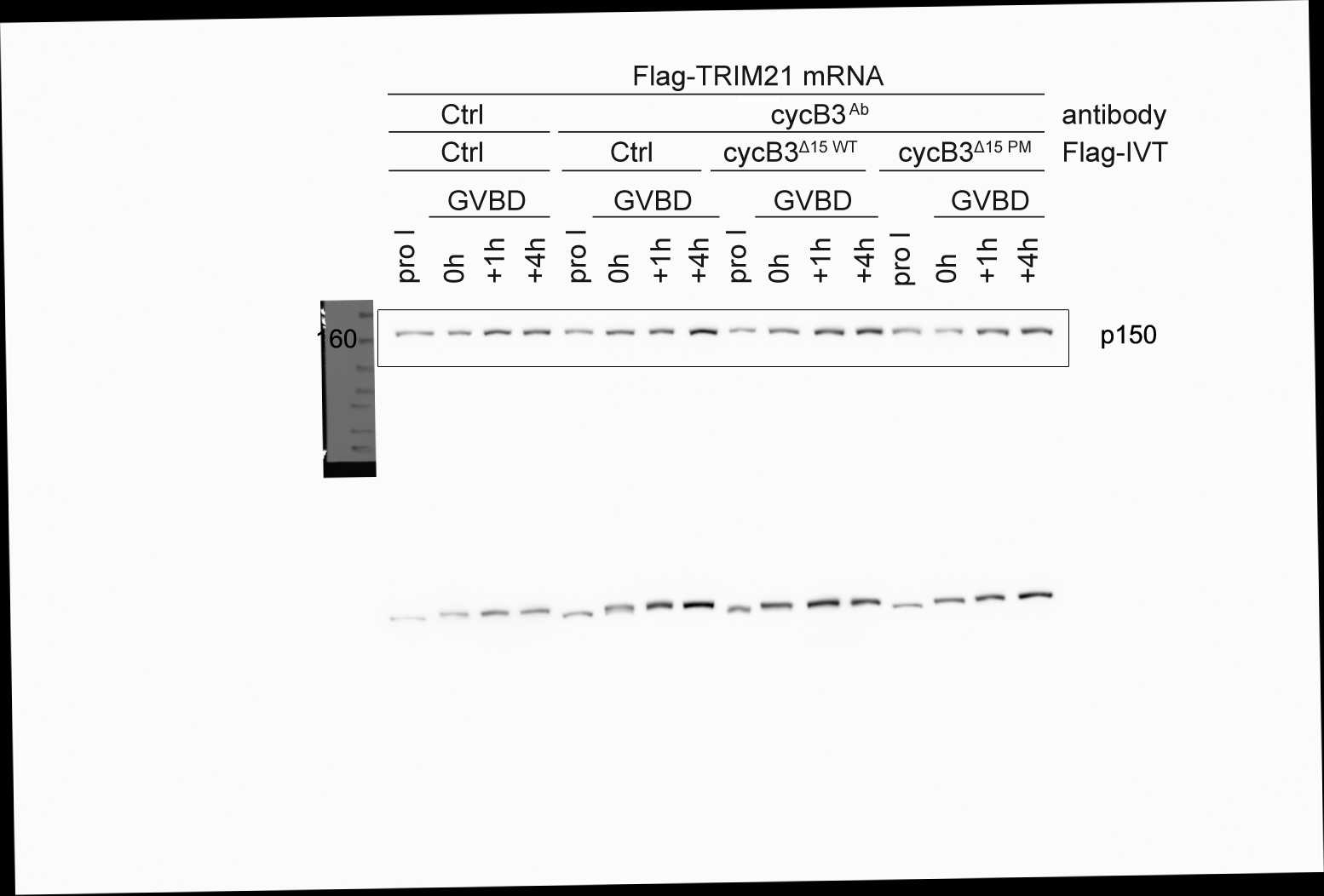

Supplement: Supplementary file 3 — Source data Fig. 2 [file 44319_2024_347_MOESM3_ESM.zip › Figure 2/2E/Western p150 (loading control cycB2).tif]

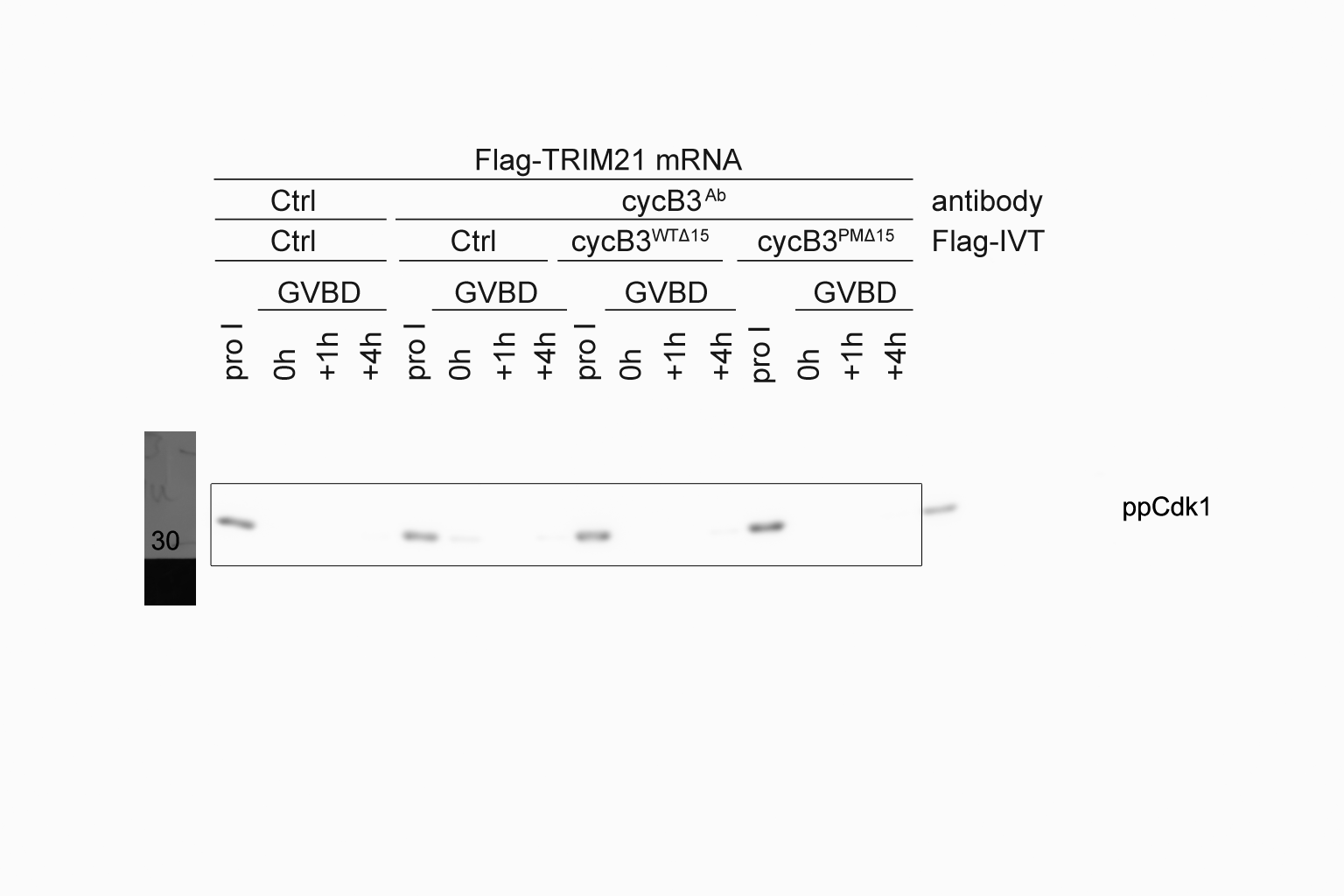

Supplement: Supplementary file 3 — Source data Fig. 2 [file 44319_2024_347_MOESM3_ESM.zip › Figure 2/2E/Western ppCdk1.tif]

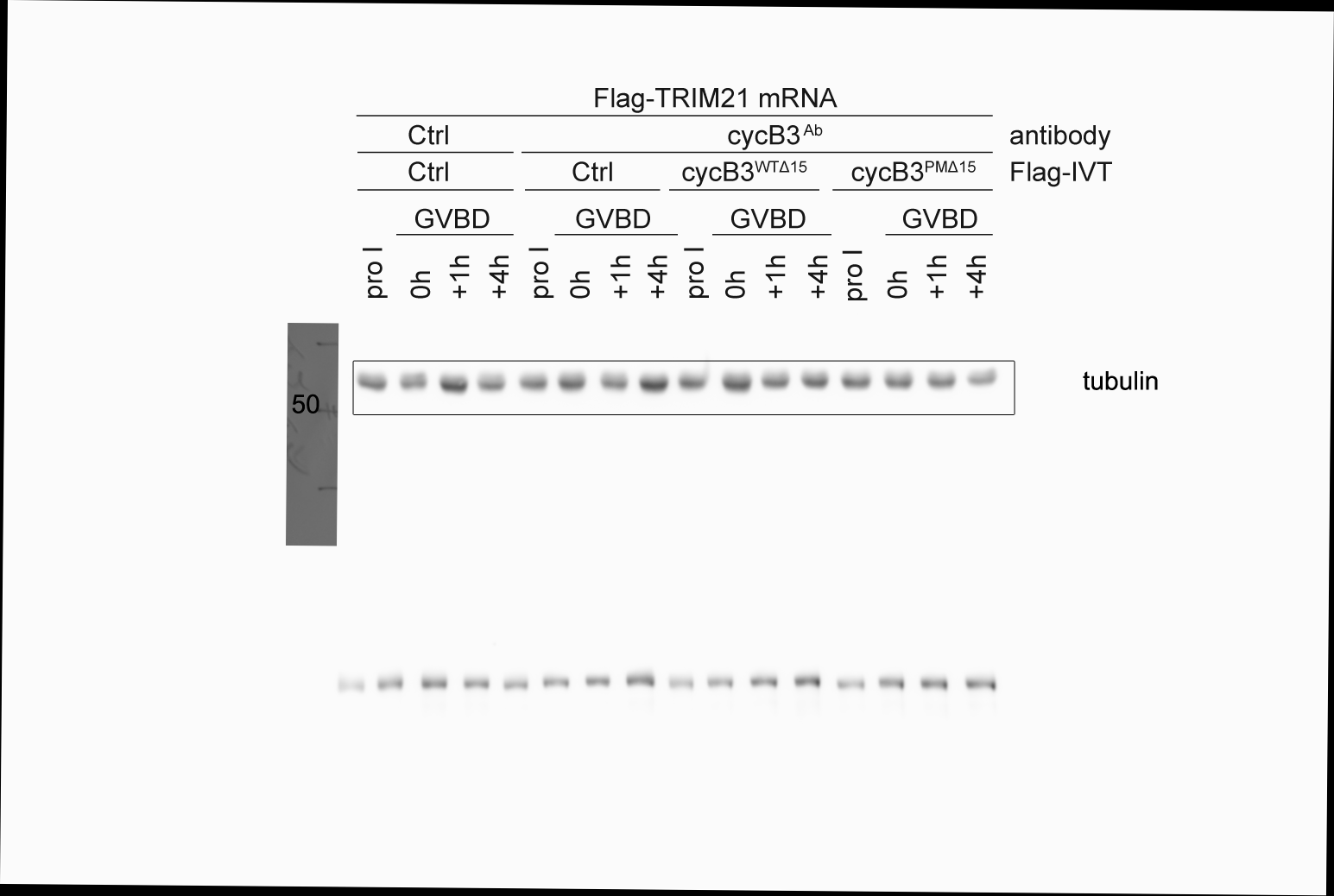

Supplement: Supplementary file 3 — Source data Fig. 2 [file 44319_2024_347_MOESM3_ESM.zip › Figure 2/2E/Western tubulin (loading control XErp1).tif]

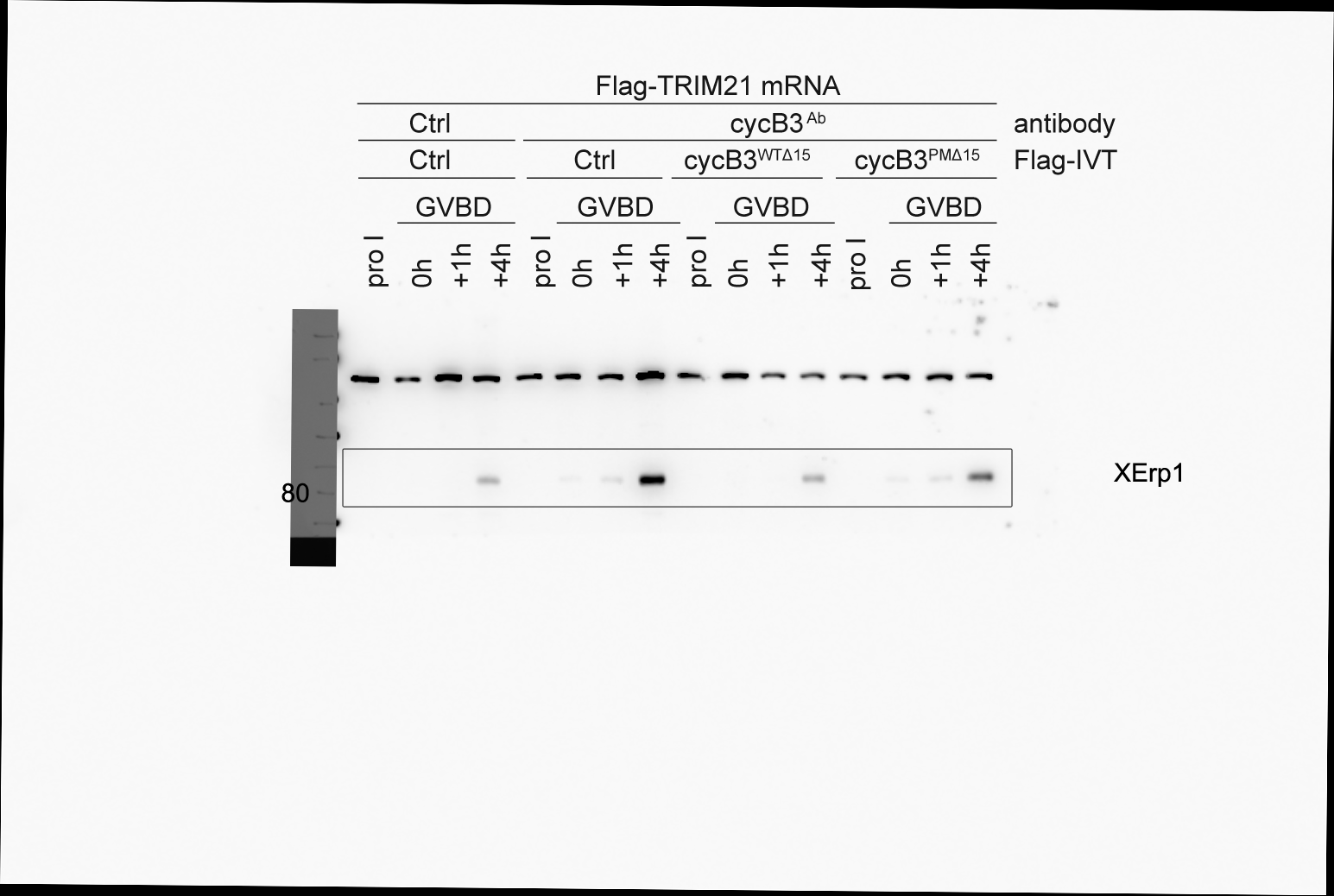

Supplement: Supplementary file 3 — Source data Fig. 2 [file 44319_2024_347_MOESM3_ESM.zip › Figure 2/2E/Western XErp1 (lambda-treated).tif]

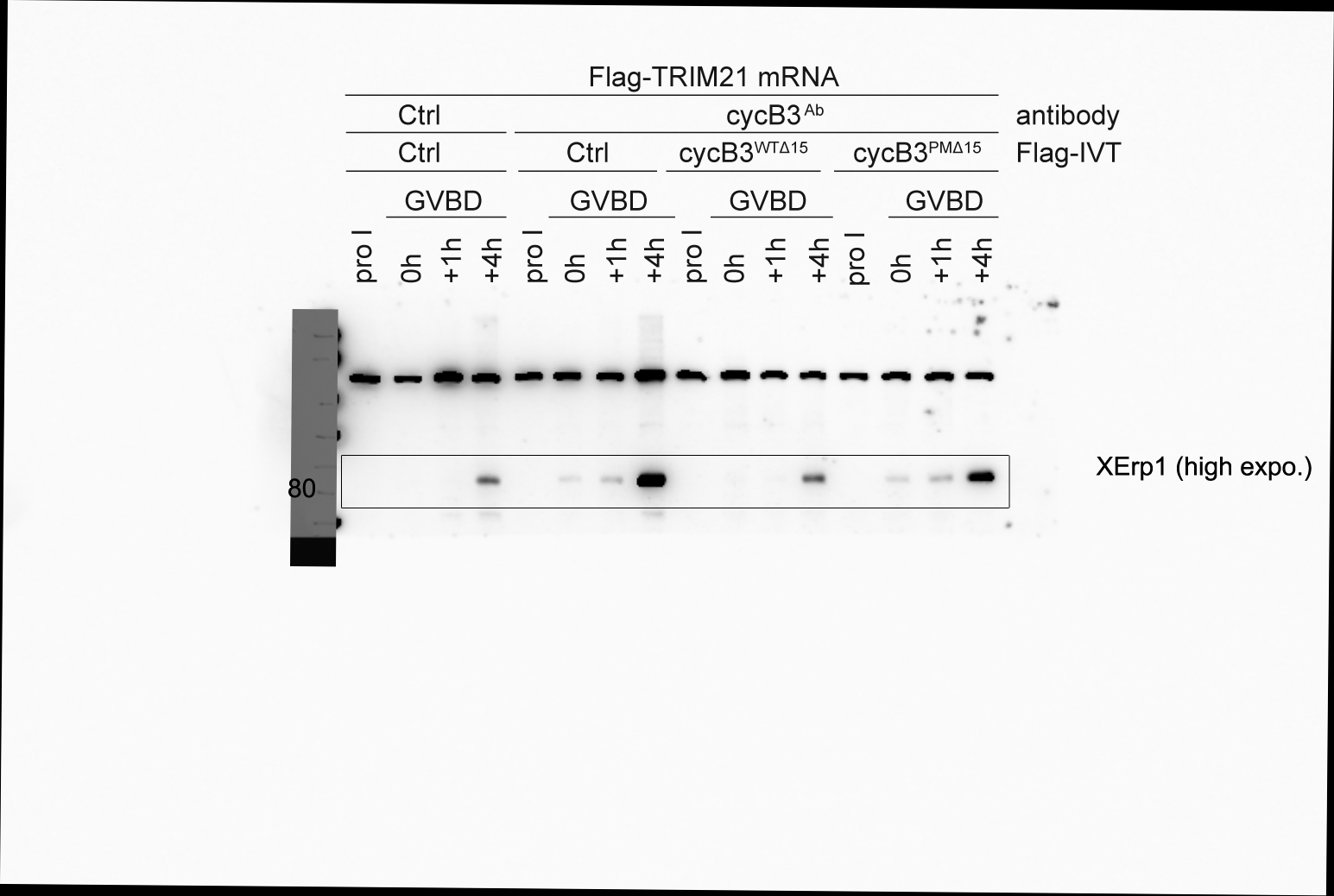

Supplement: Supplementary file 3 — Source data Fig. 2 [file 44319_2024_347_MOESM3_ESM.zip › Figure 2/2E/Western XErp1 (lambda-treated, high exposure).tif]

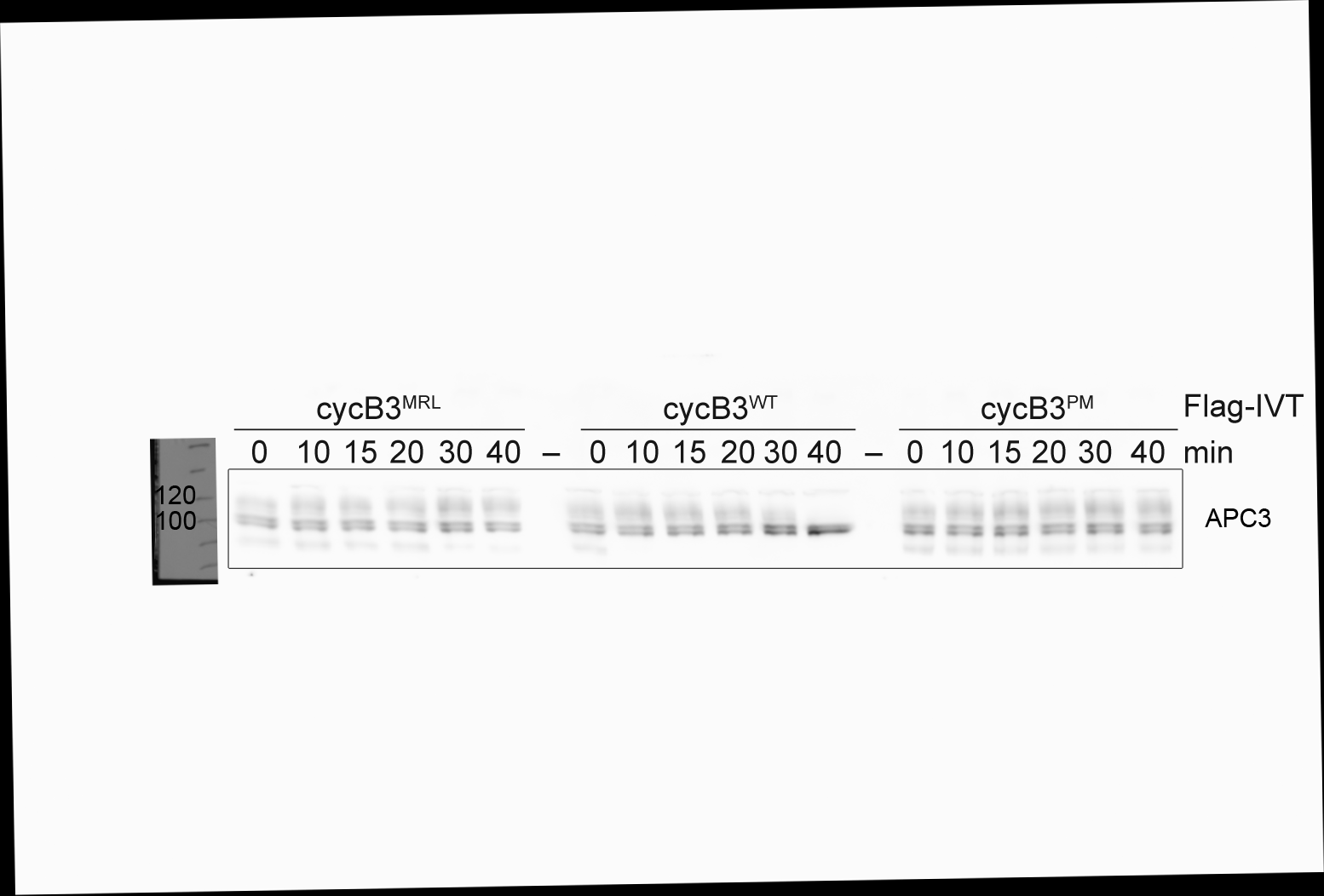

Supplement: Supplementary file 4 — Source data Fig. 3 [file 44319_2024_347_MOESM4_ESM.zip › Figure 3/3A/Western APC3.tif]

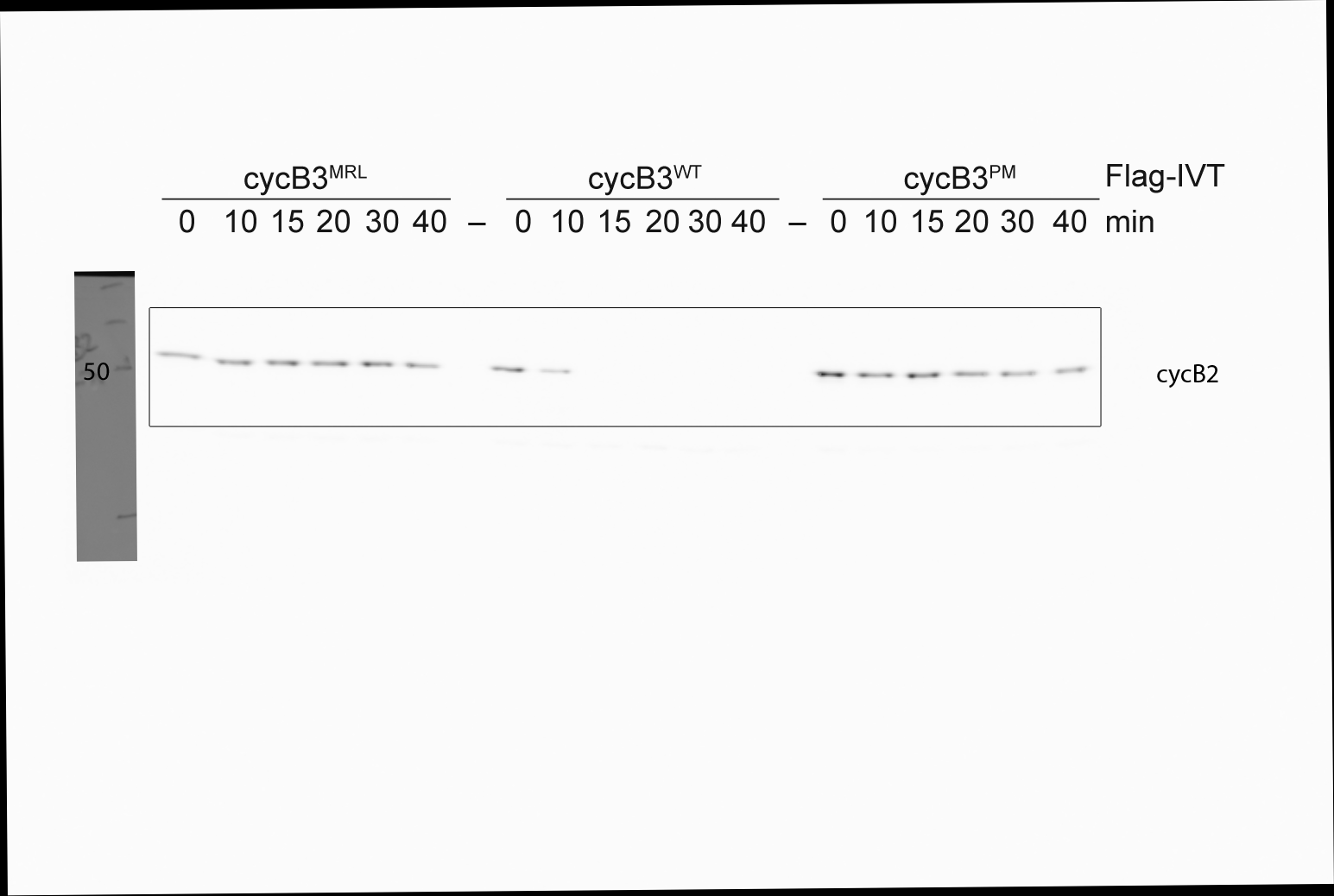

Supplement: Supplementary file 4 — Source data Fig. 3 [file 44319_2024_347_MOESM4_ESM.zip › Figure 3/3A/Western cycB2.tif]

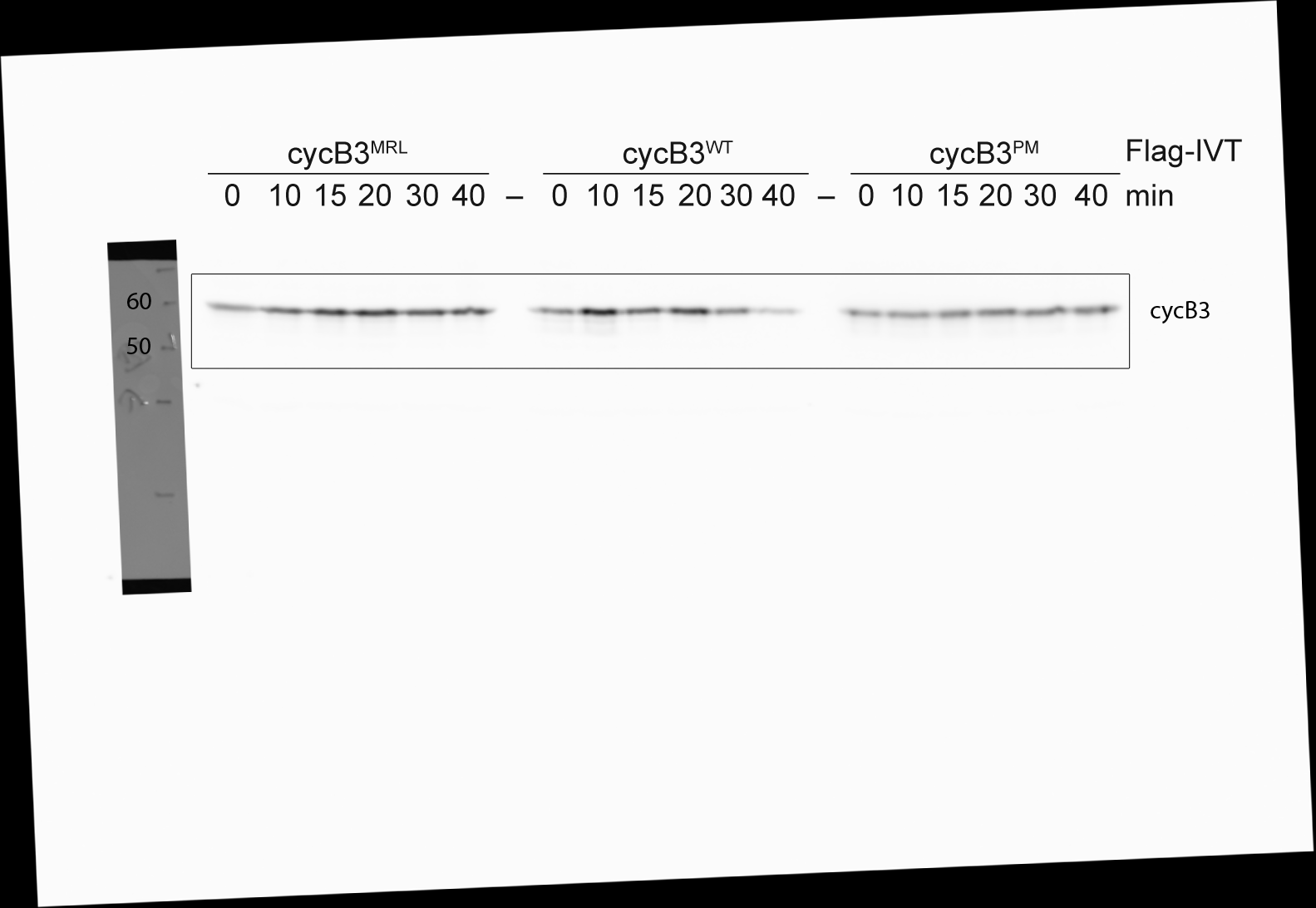

Supplement: Supplementary file 4 — Source data Fig. 3 [file 44319_2024_347_MOESM4_ESM.zip › Figure 3/3A/Western cycB3.tif]

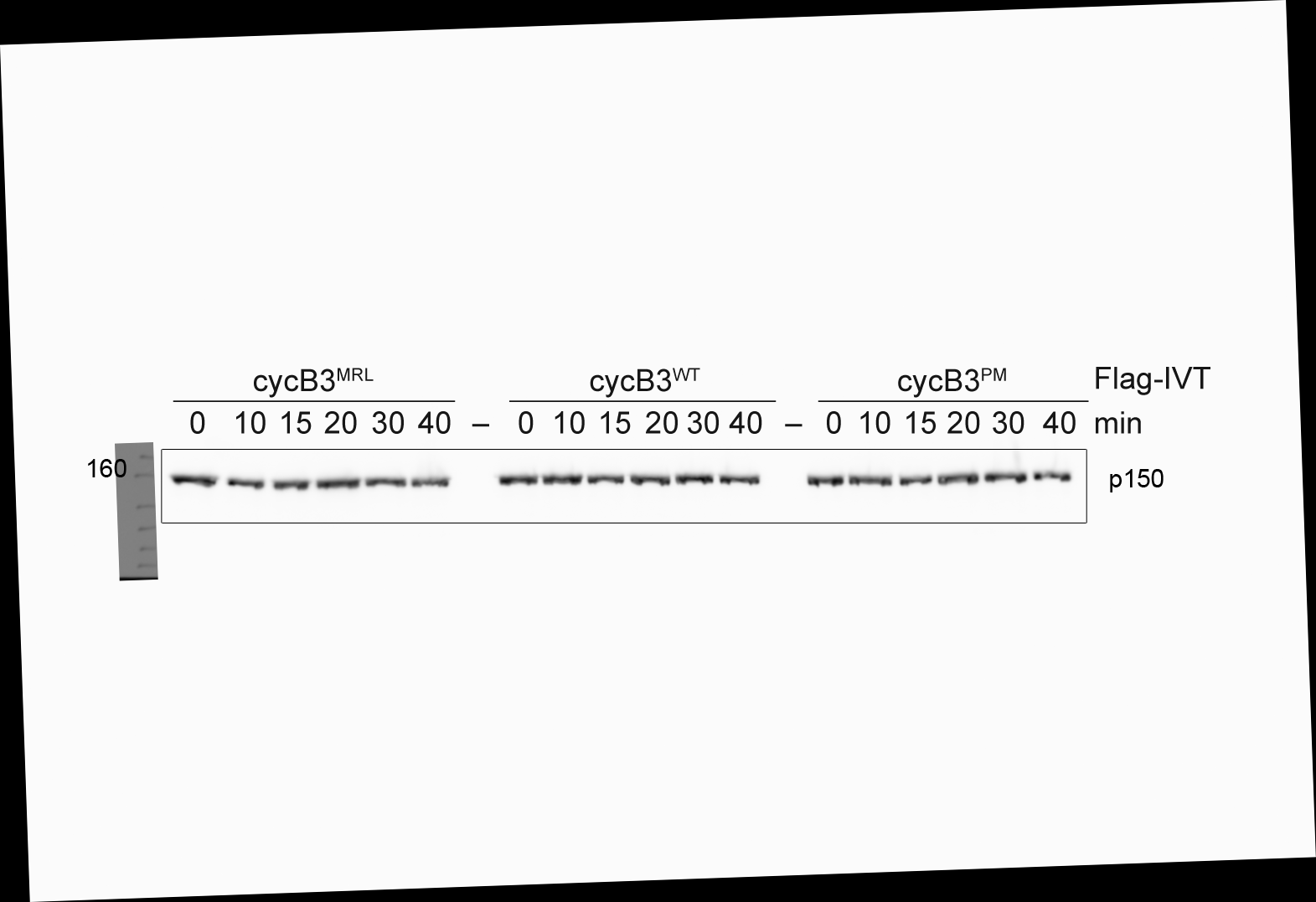

Supplement: Supplementary file 4 — Source data Fig. 3 [file 44319_2024_347_MOESM4_ESM.zip › Figure 3/3A/Western p150.tif]

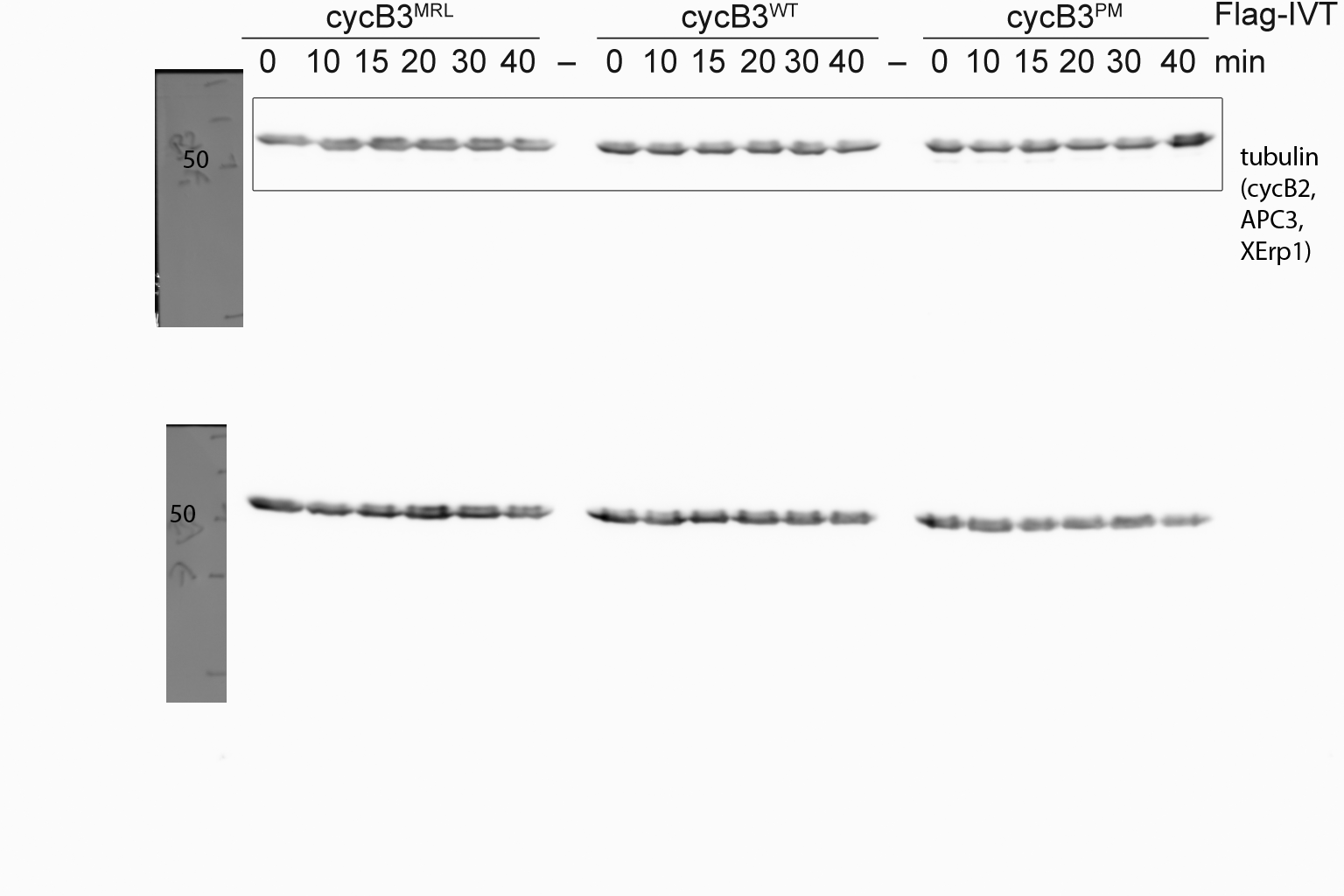

Supplement: Supplementary file 4 — Source data Fig. 3 [file 44319_2024_347_MOESM4_ESM.zip › Figure 3/3A/Western tubulin.tif]

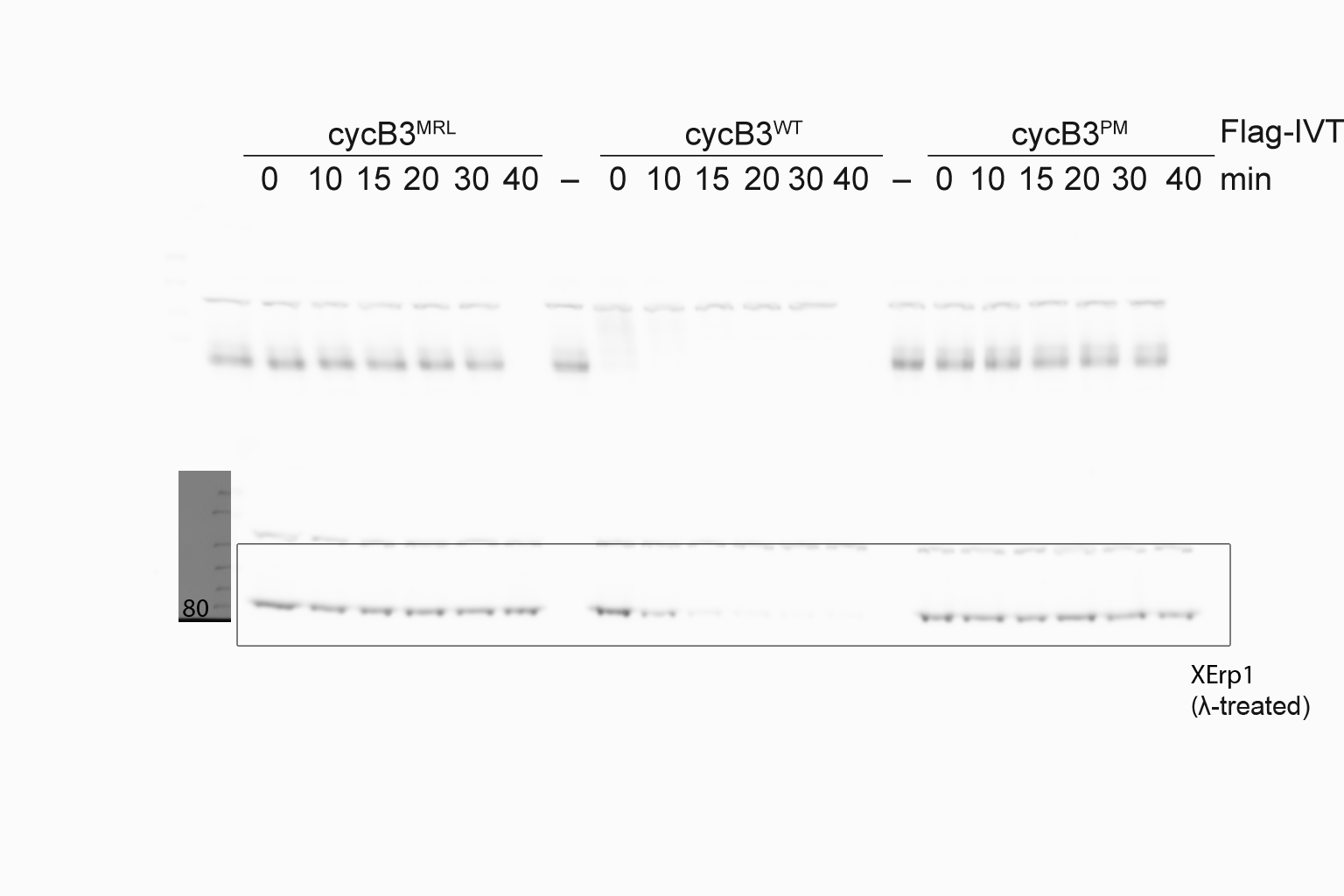

Supplement: Supplementary file 4 — Source data Fig. 3 [file 44319_2024_347_MOESM4_ESM.zip › Figure 3/3A/Western XErp1 lambda-treated.tif]

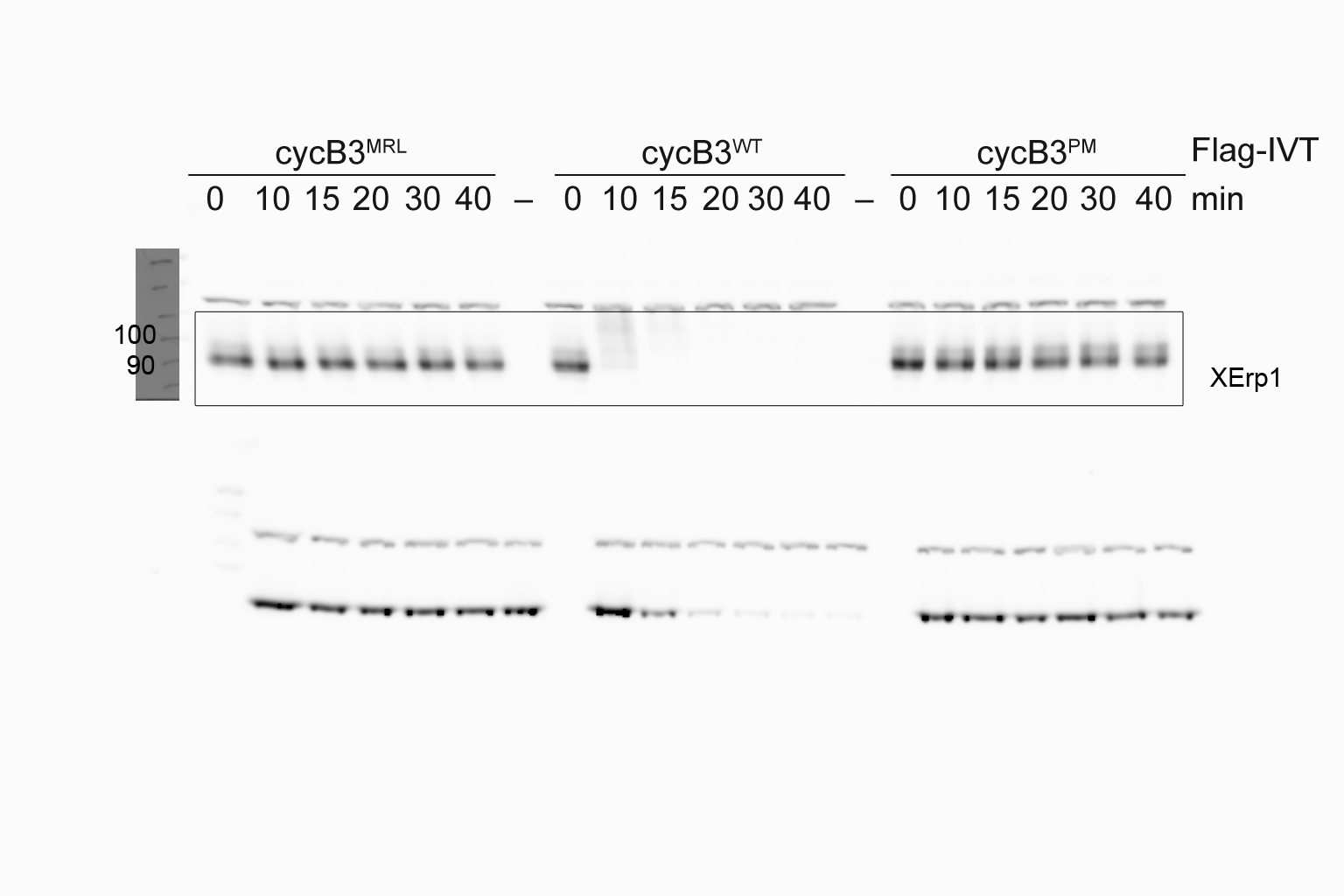

Supplement: Supplementary file 4 — Source data Fig. 3 [file 44319_2024_347_MOESM4_ESM.zip › Figure 3/3A/Western XErp1.tif]

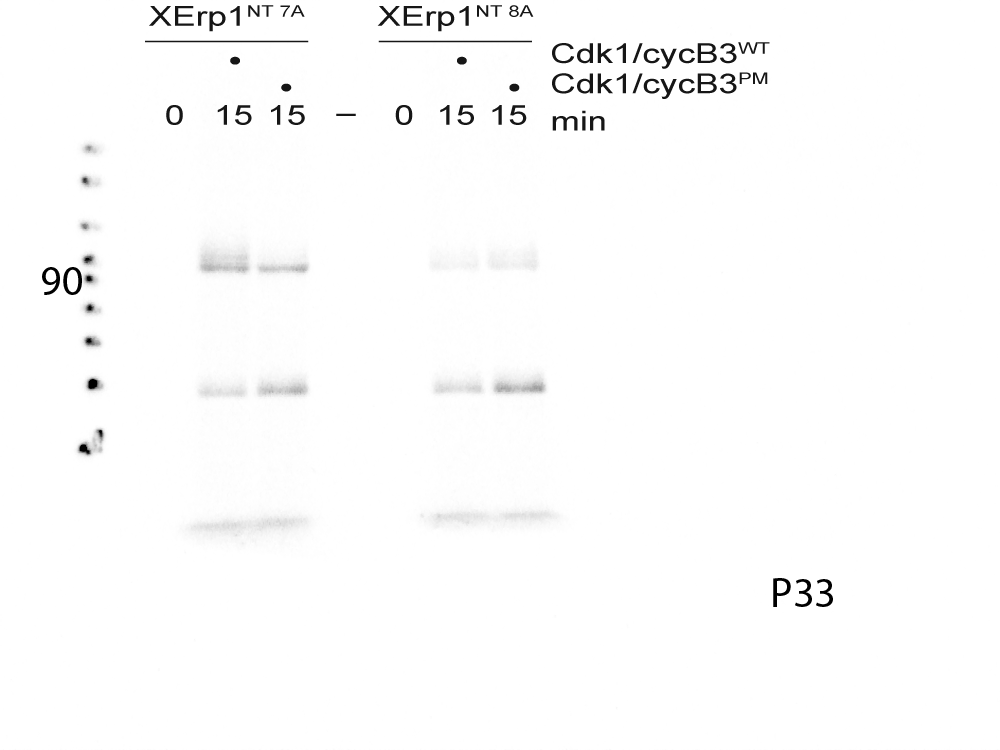

Supplement: Supplementary file 4 — Source data Fig. 3 [file 44319_2024_347_MOESM4_ESM.zip › Figure 3/3D/Autoradiogram P33.tif]

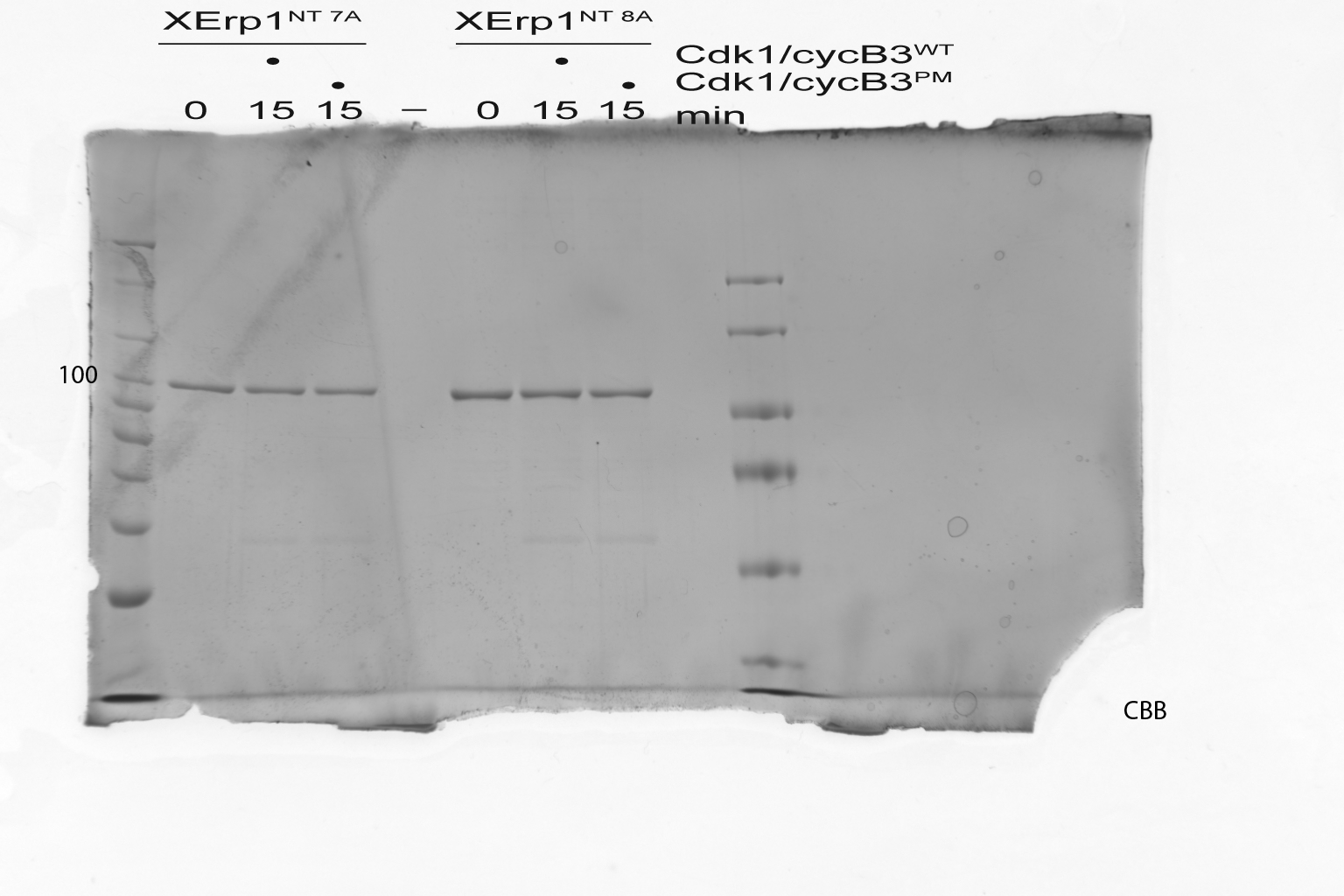

Supplement: Supplementary file 4 — Source data Fig. 3 [file 44319_2024_347_MOESM4_ESM.zip › Figure 3/3D/Coomassie.tif]

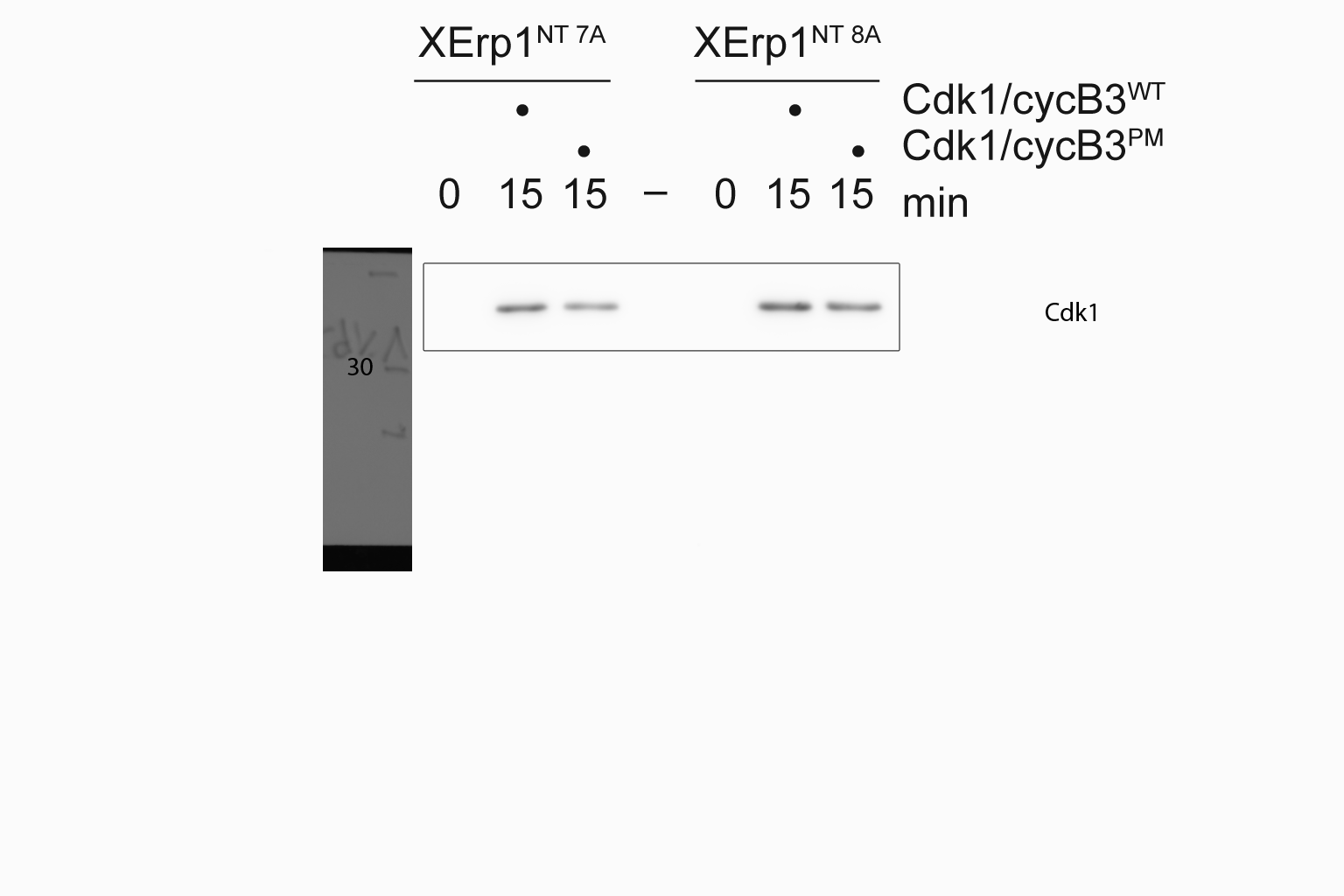

Supplement: Supplementary file 4 — Source data Fig. 3 [file 44319_2024_347_MOESM4_ESM.zip › Figure 3/3D/Western Cdk1.tif]

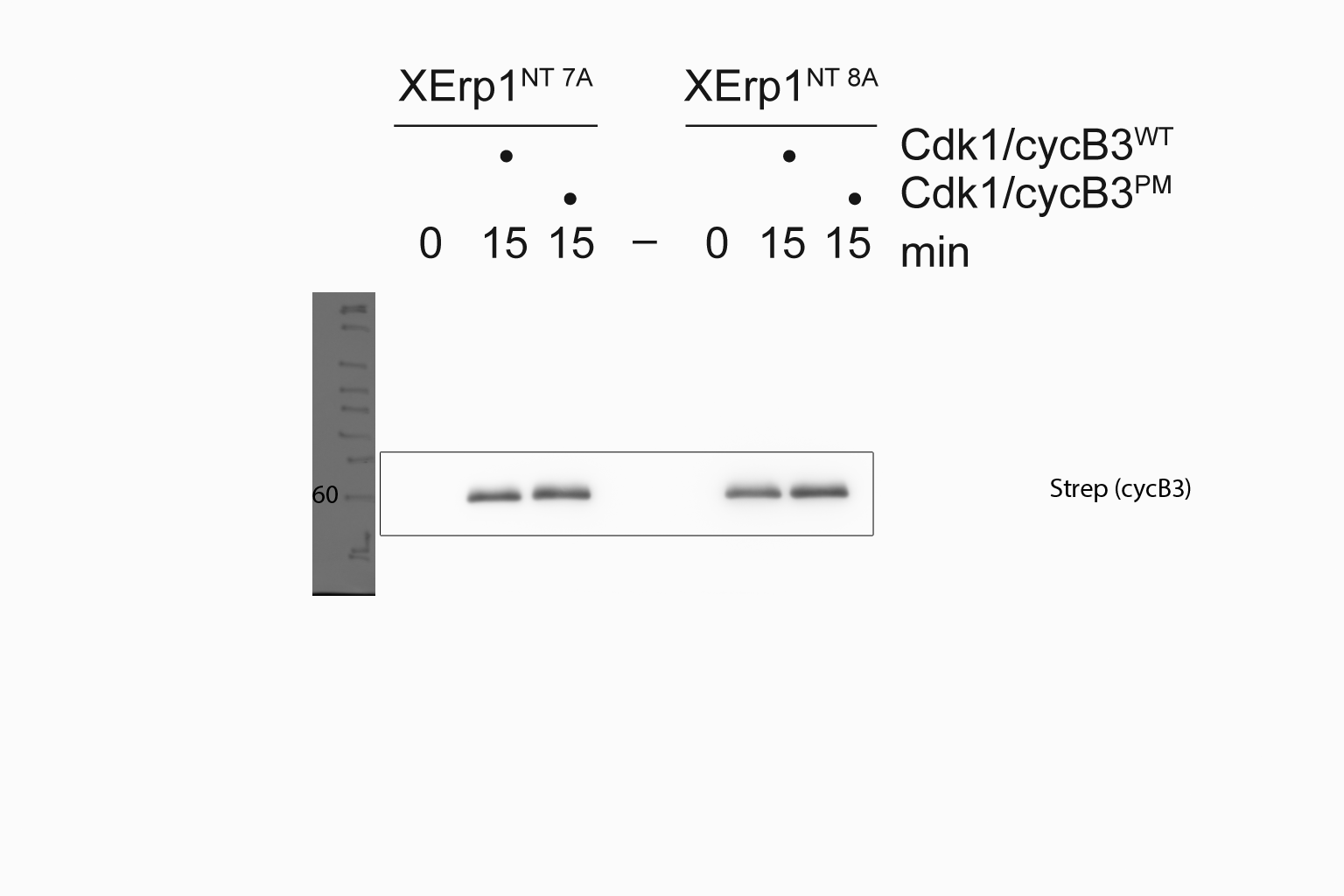

Supplement: Supplementary file 4 — Source data Fig. 3 [file 44319_2024_347_MOESM4_ESM.zip › Figure 3/3D/Western Strep.tif]

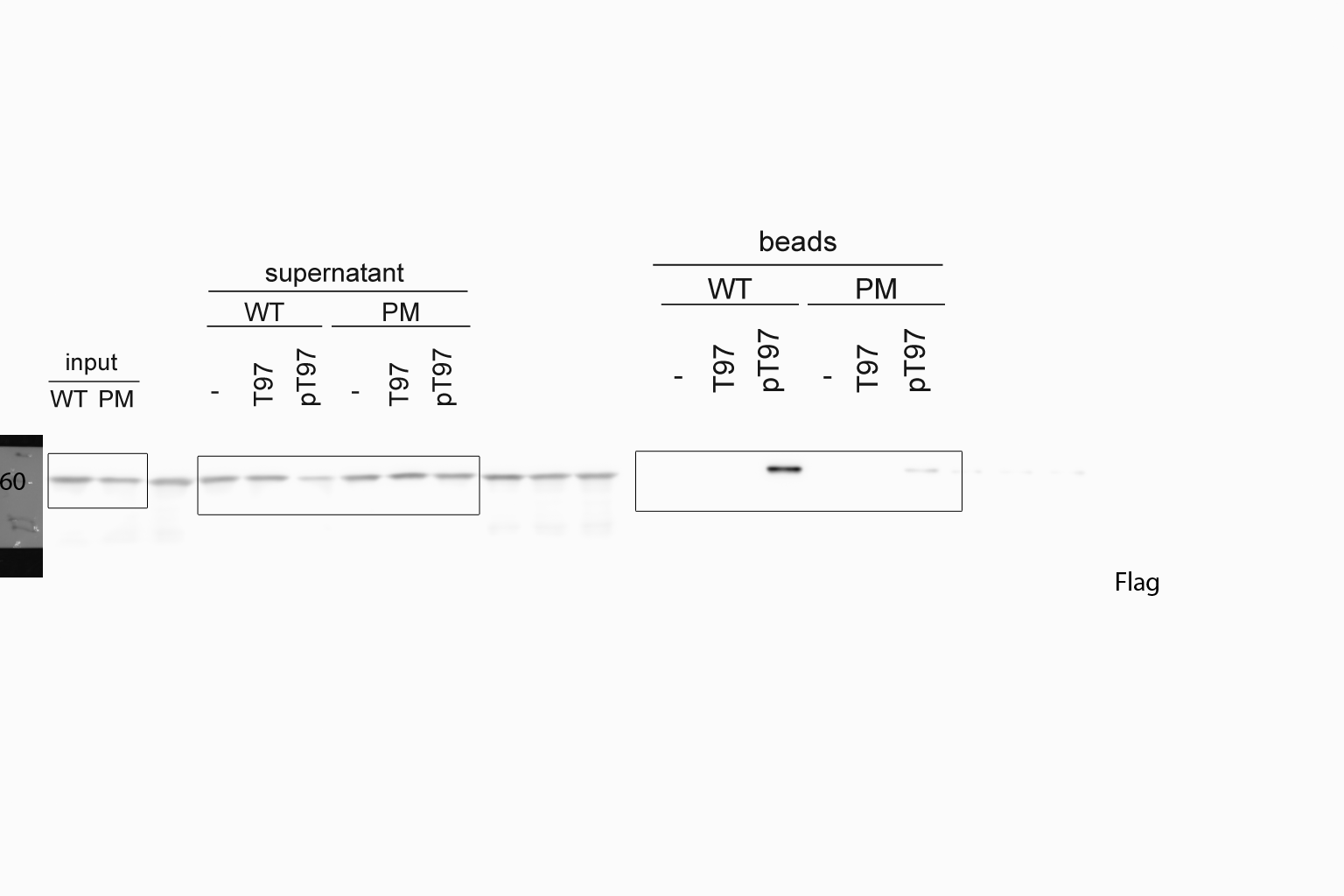

Supplement: Supplementary file 5 — Source data Fig. 4 [file 44319_2024_347_MOESM5_ESM.zip › Figure 4/4B/Western Flag.tif]

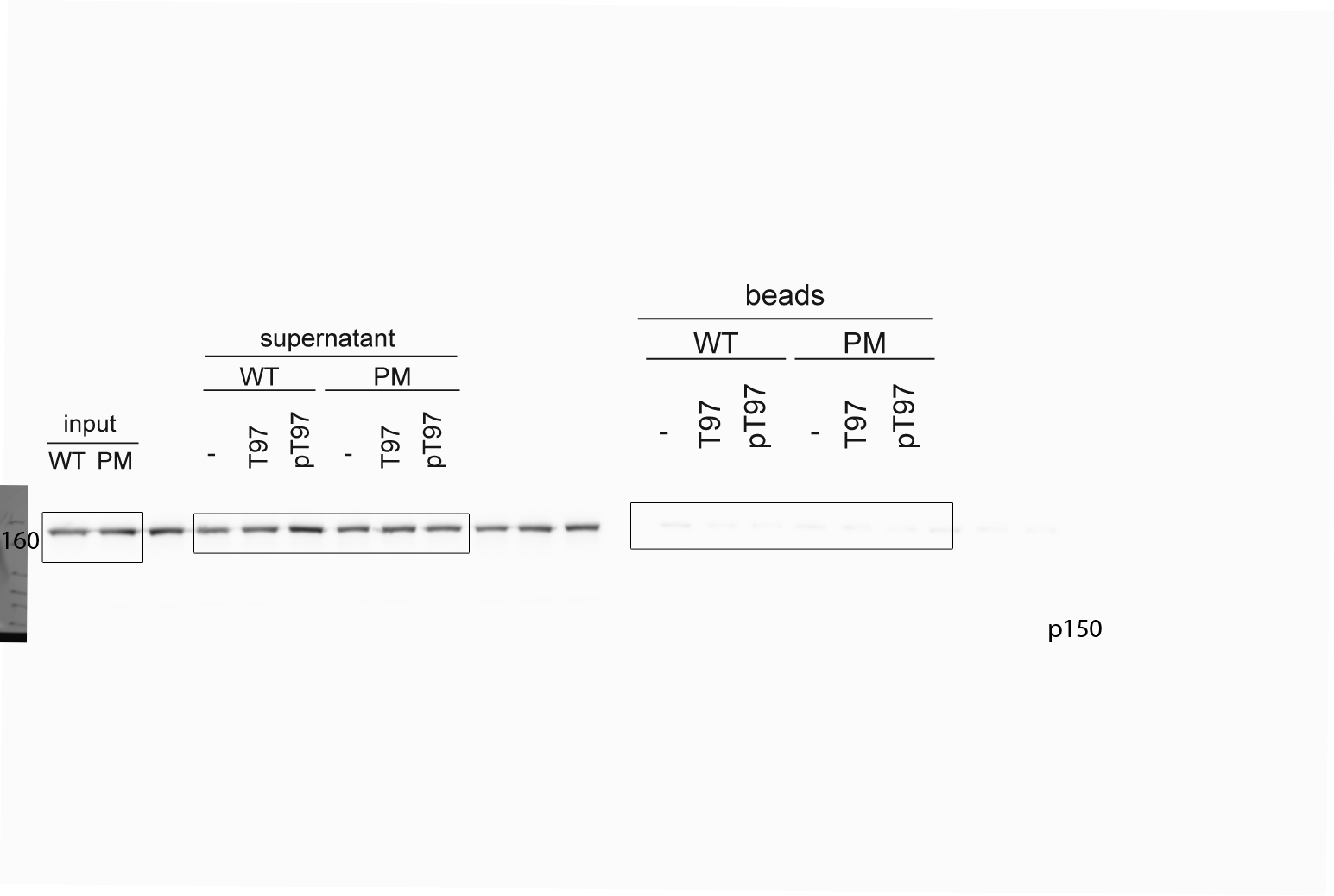

Supplement: Supplementary file 5 — Source data Fig. 4 [file 44319_2024_347_MOESM5_ESM.zip › Figure 4/4B/Western p150.tif]

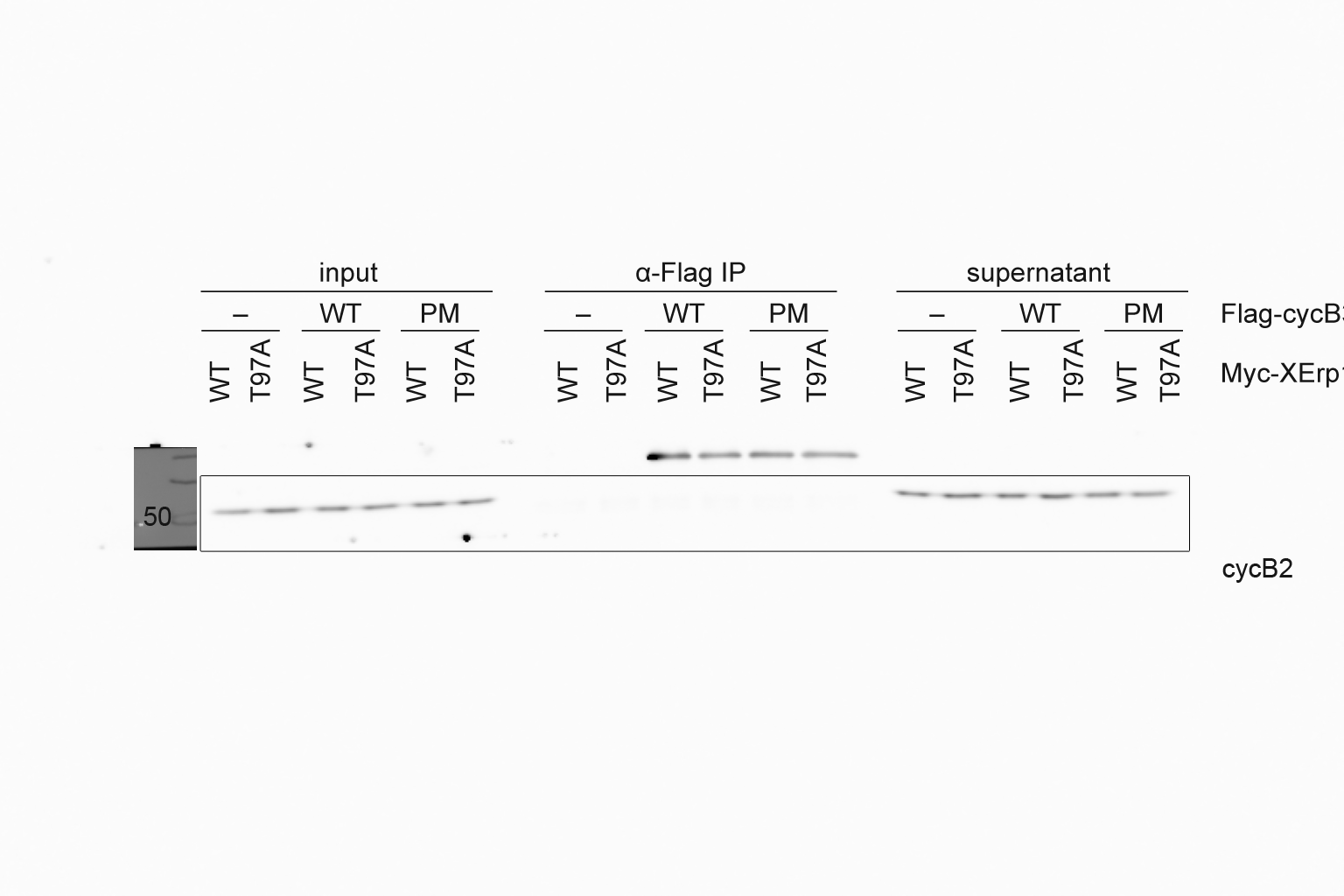

Supplement: Supplementary file 5 — Source data Fig. 4 [file 44319_2024_347_MOESM5_ESM.zip › Figure 4/4C/Western cycB2.tif]

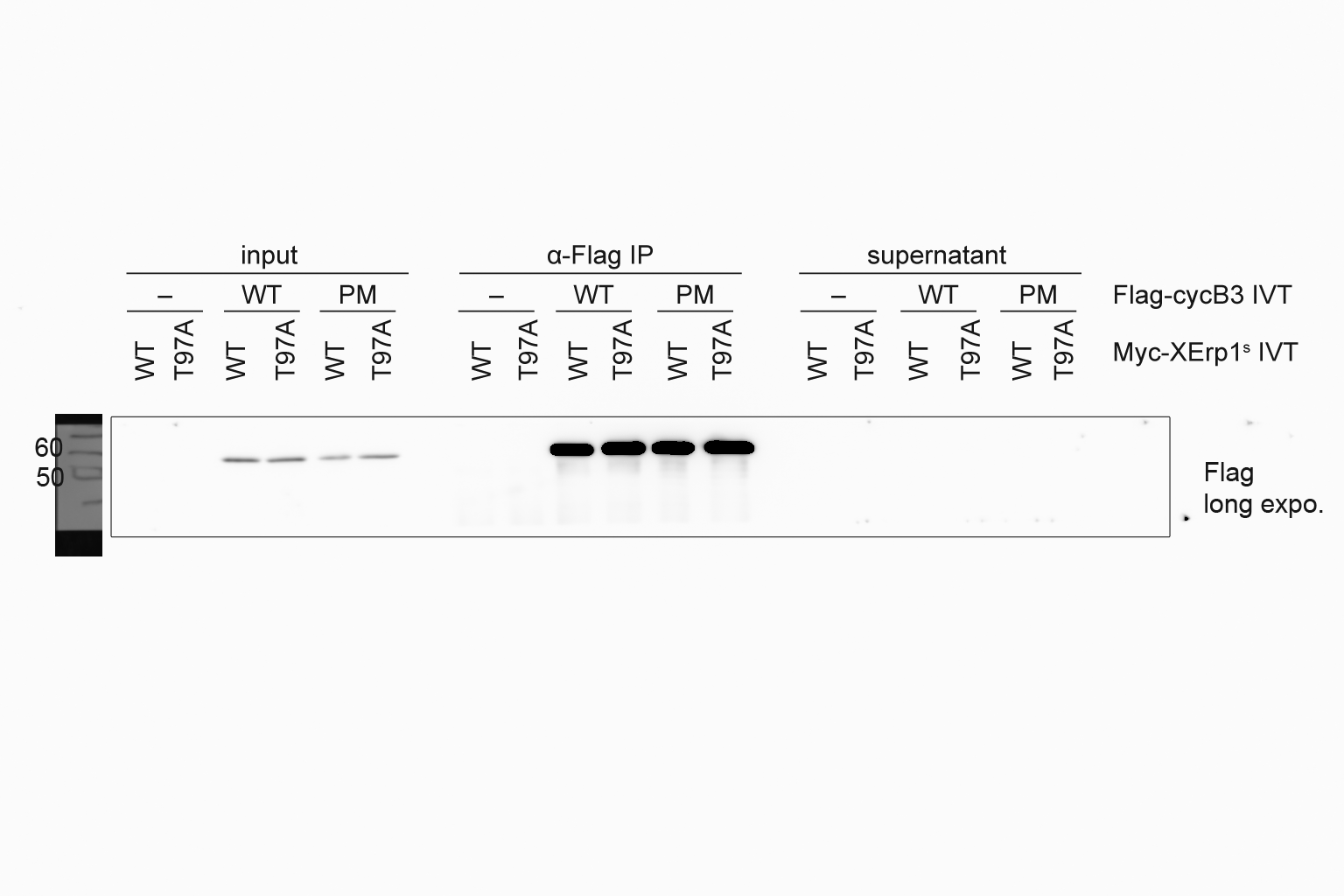

Supplement: Supplementary file 5 — Source data Fig. 4 [file 44319_2024_347_MOESM5_ESM.zip › Figure 4/4C/Western Flag long expo.tif]

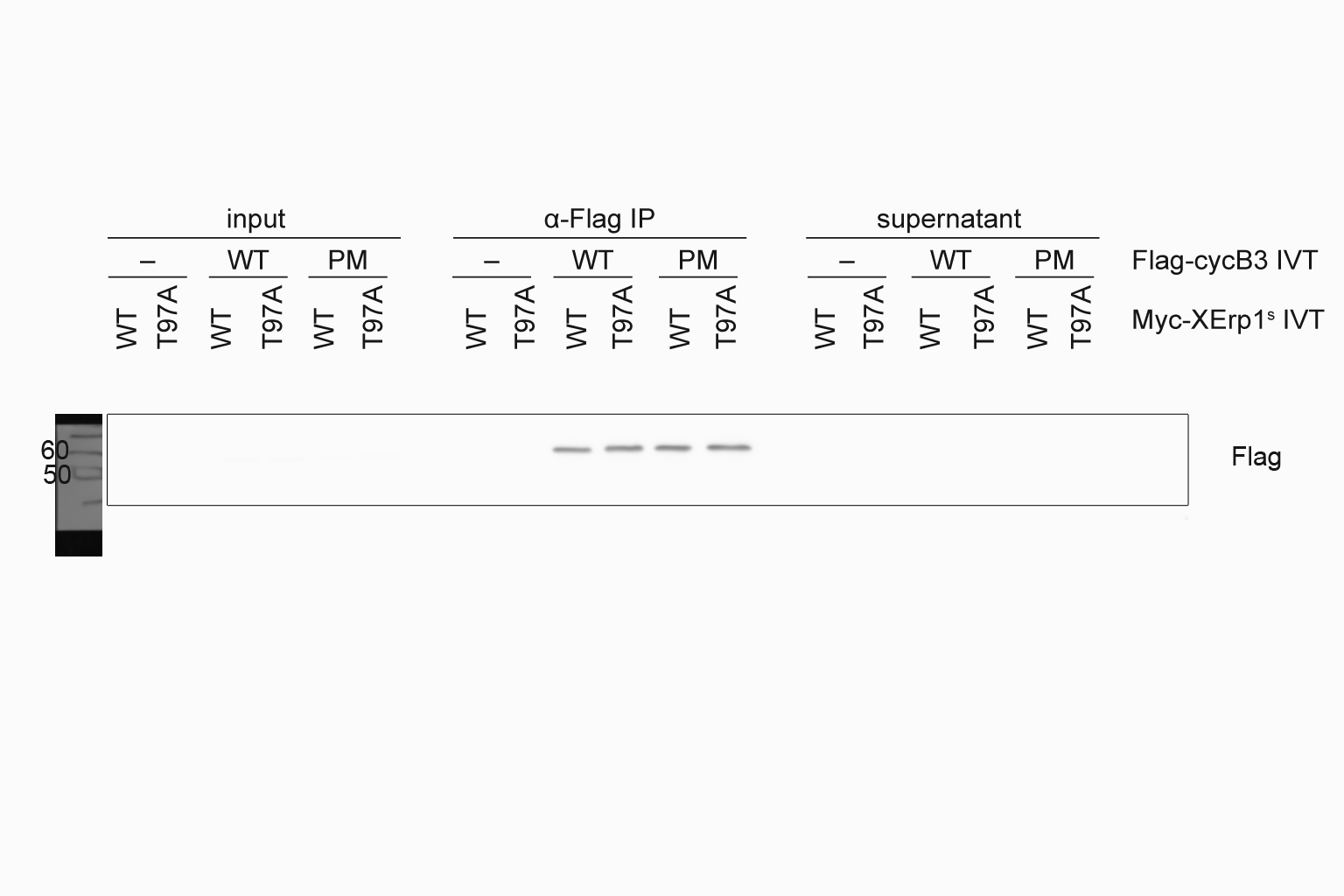

Supplement: Supplementary file 5 — Source data Fig. 4 [file 44319_2024_347_MOESM5_ESM.zip › Figure 4/4C/Western Flag.tif]

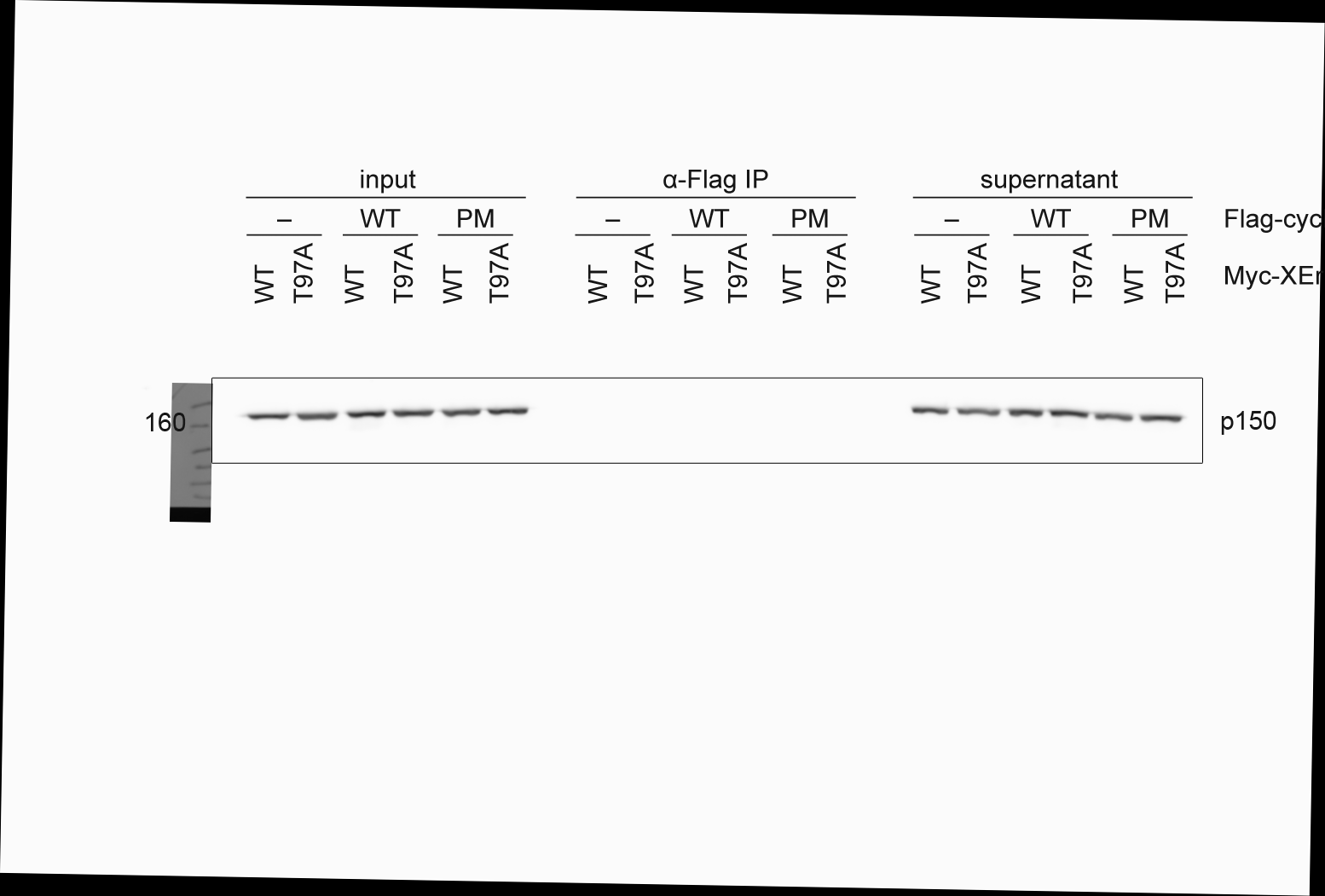

Supplement: Supplementary file 5 — Source data Fig. 4 [file 44319_2024_347_MOESM5_ESM.zip › Figure 4/4C/Western p150 (cycB2).tif]

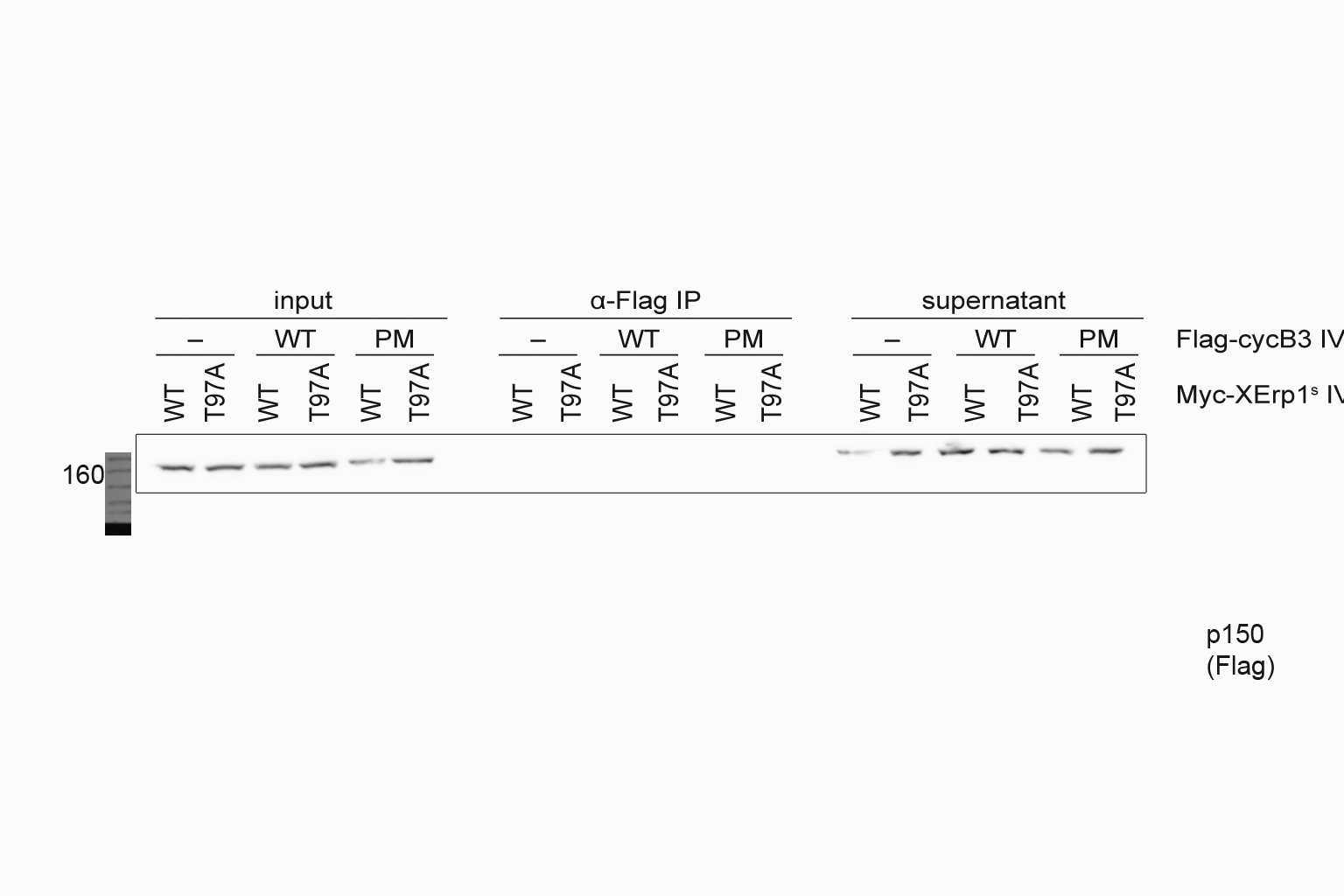

Supplement: Supplementary file 5 — Source data Fig. 4 [file 44319_2024_347_MOESM5_ESM.zip › Figure 4/4C/Western p150 (Flag).tif]

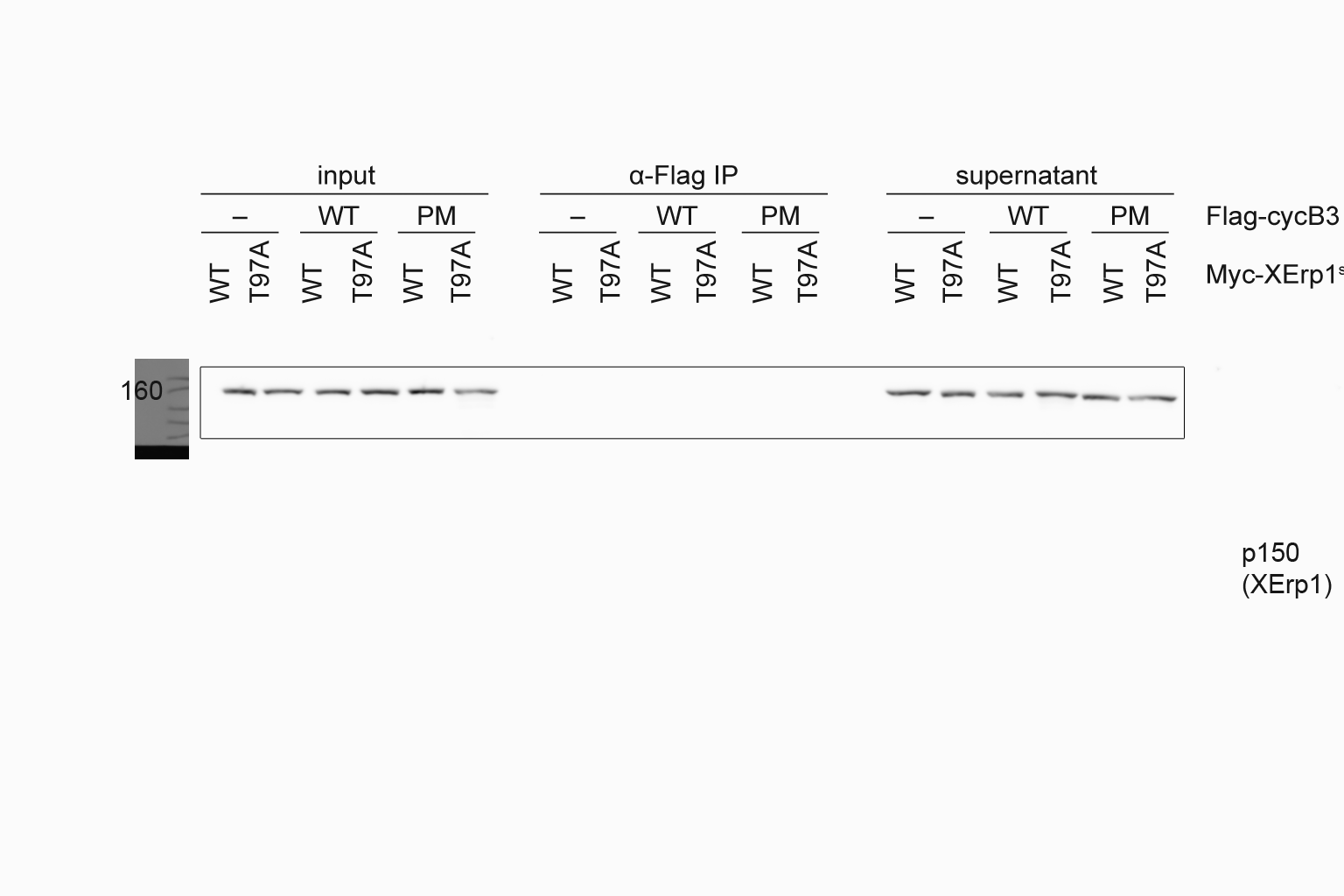

Supplement: Supplementary file 5 — Source data Fig. 4 [file 44319_2024_347_MOESM5_ESM.zip › Figure 4/4C/Western p150 (XErp1).tif]

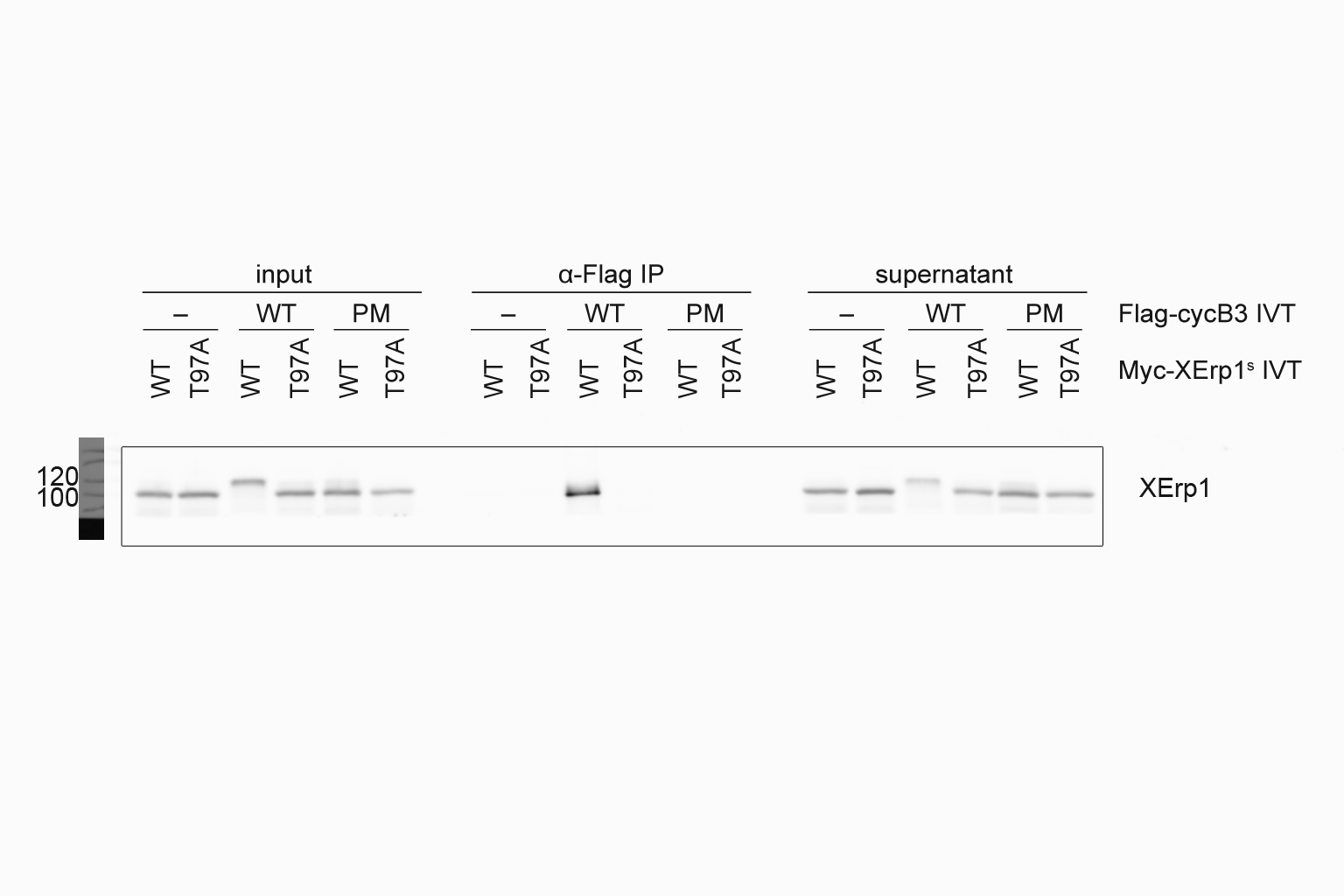

Supplement: Supplementary file 5 — Source data Fig. 4 [file 44319_2024_347_MOESM5_ESM.zip › Figure 4/4C/Western XErp1.tif]

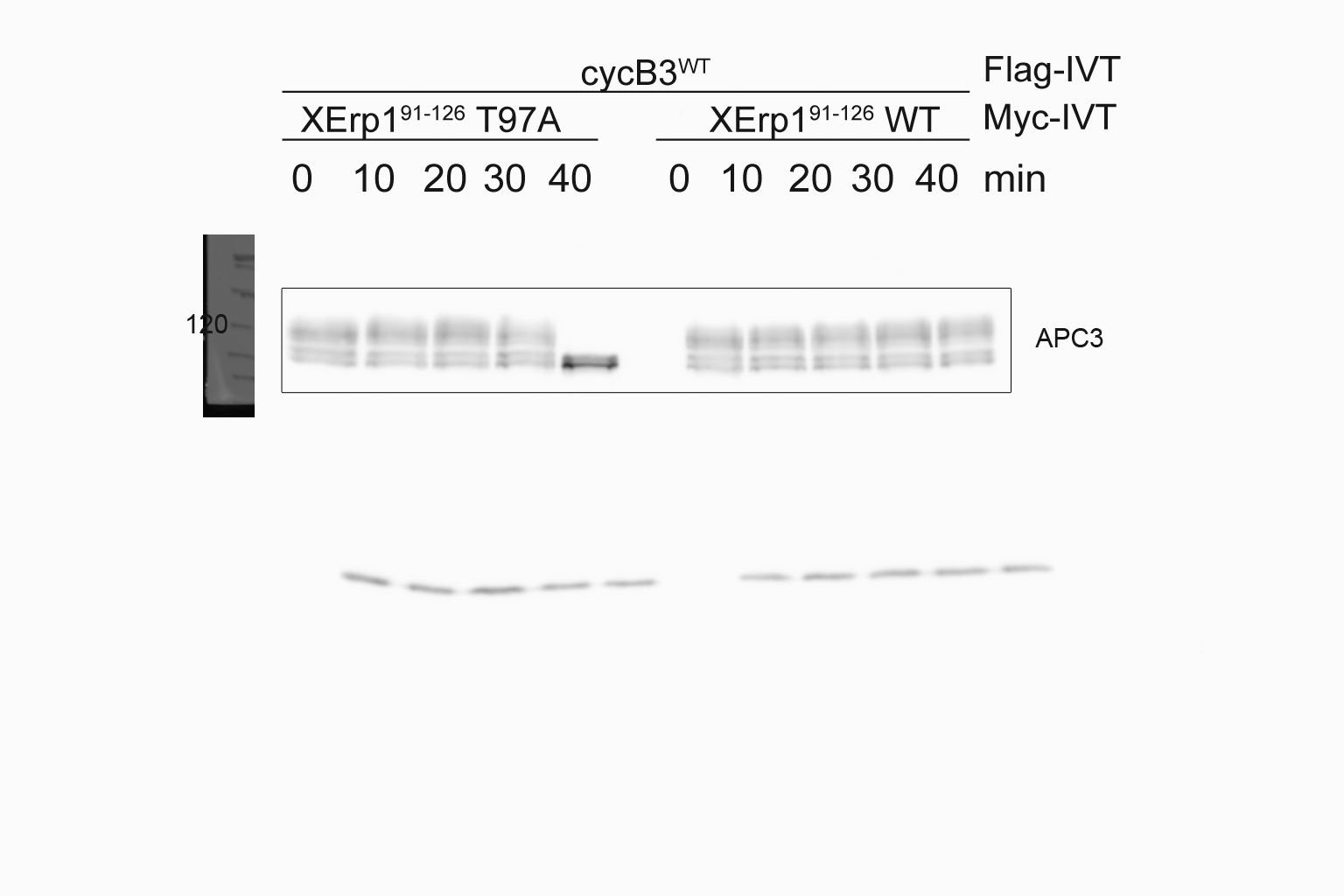

Supplement: Supplementary file 5 — Source data Fig. 4 [file 44319_2024_347_MOESM5_ESM.zip › Figure 4/4D/Western APC3.tif]

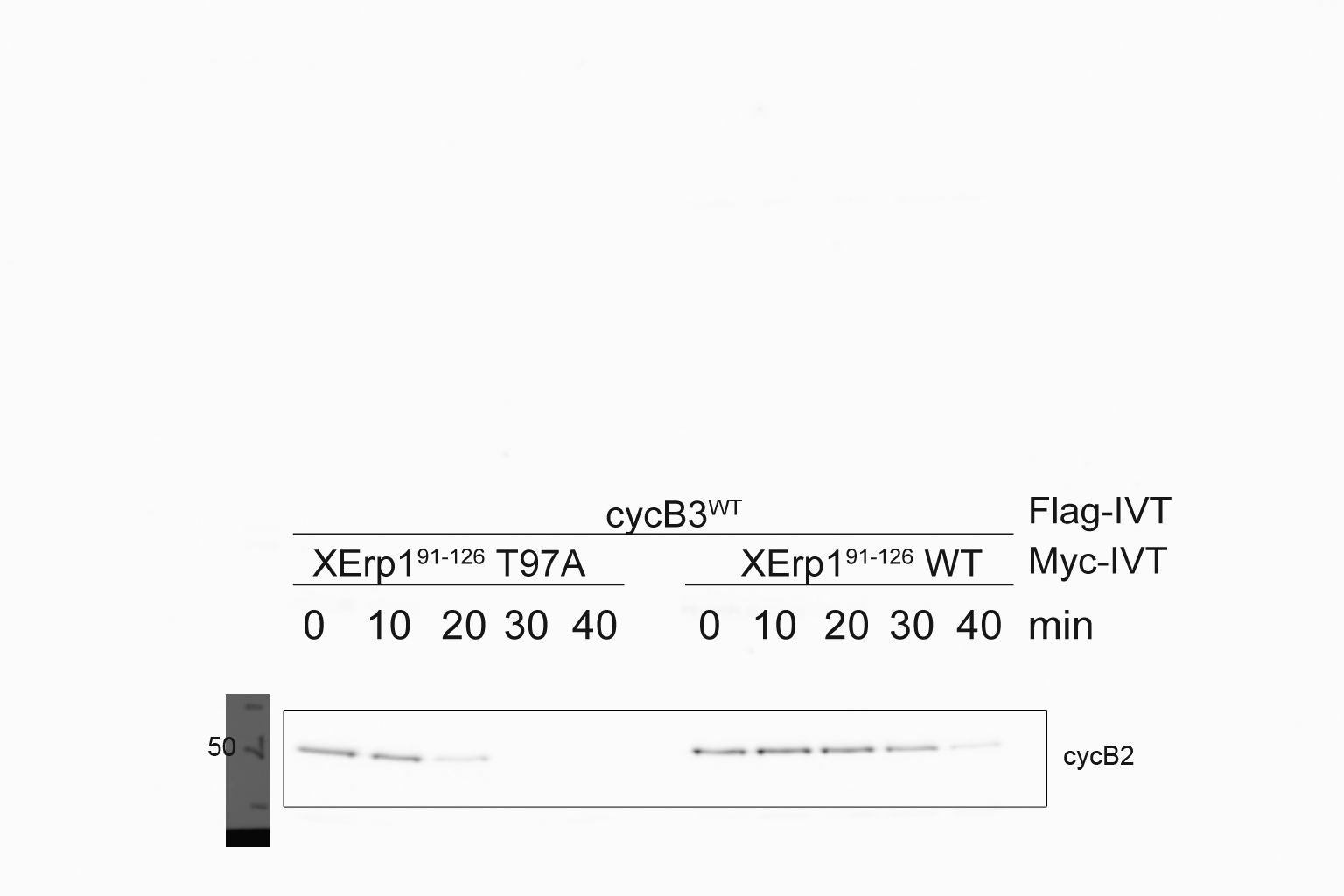

Supplement: Supplementary file 5 — Source data Fig. 4 [file 44319_2024_347_MOESM5_ESM.zip › Figure 4/4D/Western cycB2.tif]

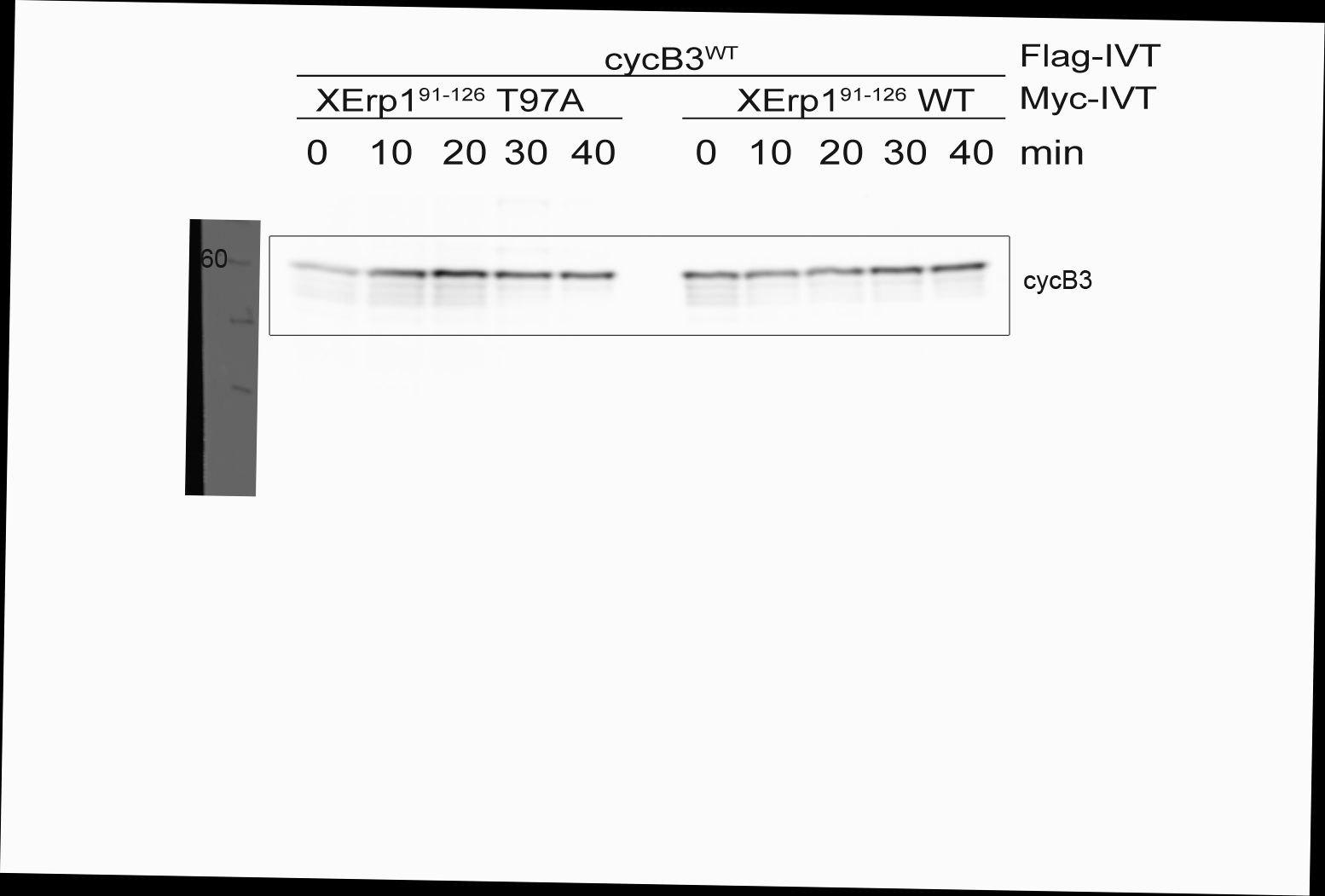

Supplement: Supplementary file 5 — Source data Fig. 4 [file 44319_2024_347_MOESM5_ESM.zip › Figure 4/4D/Western cycB3.tif]

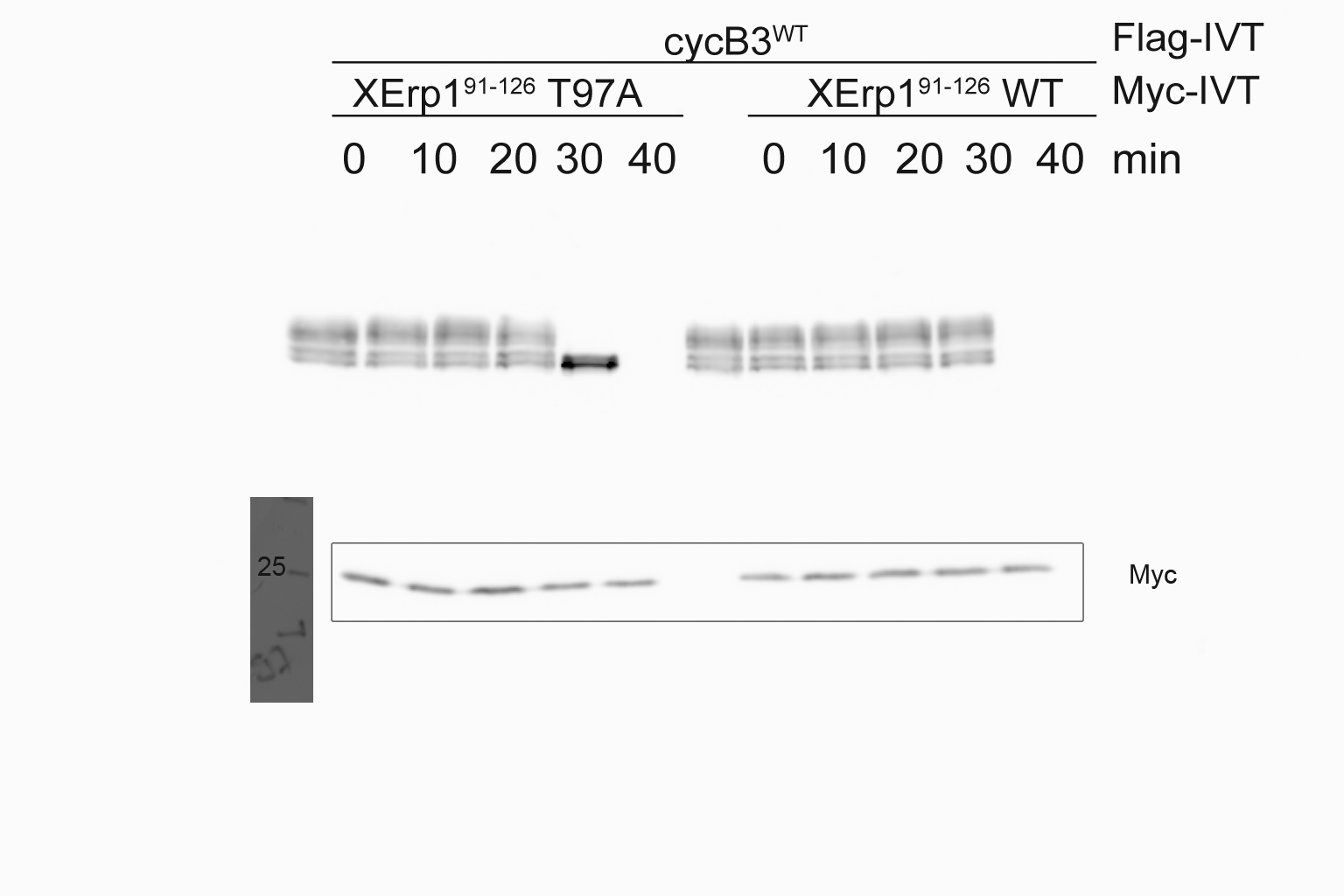

Supplement: Supplementary file 5 — Source data Fig. 4 [file 44319_2024_347_MOESM5_ESM.zip › Figure 4/4D/Western Myc.tif]

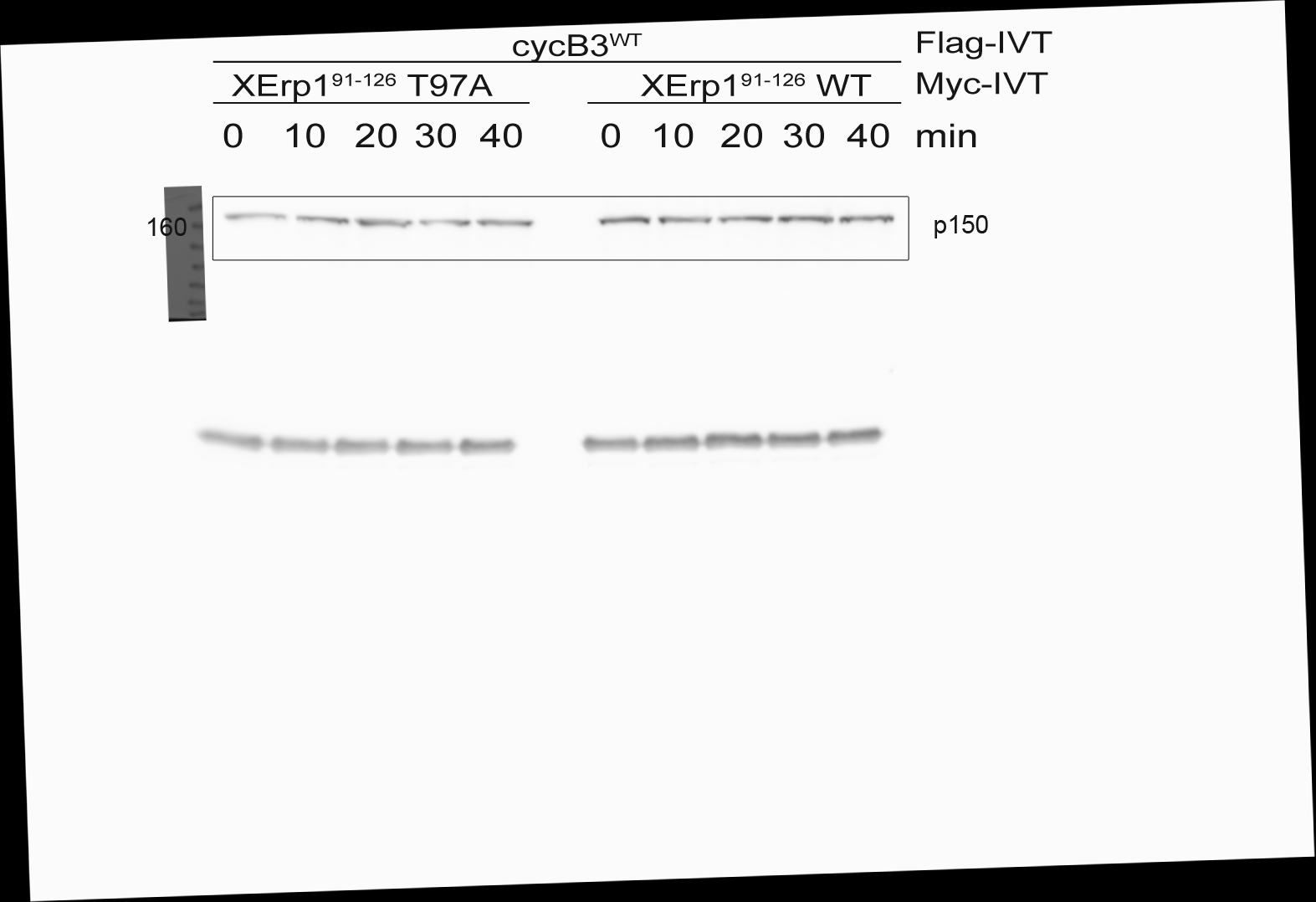

Supplement: Supplementary file 5 — Source data Fig. 4 [file 44319_2024_347_MOESM5_ESM.zip › Figure 4/4D/Western p150.tif]

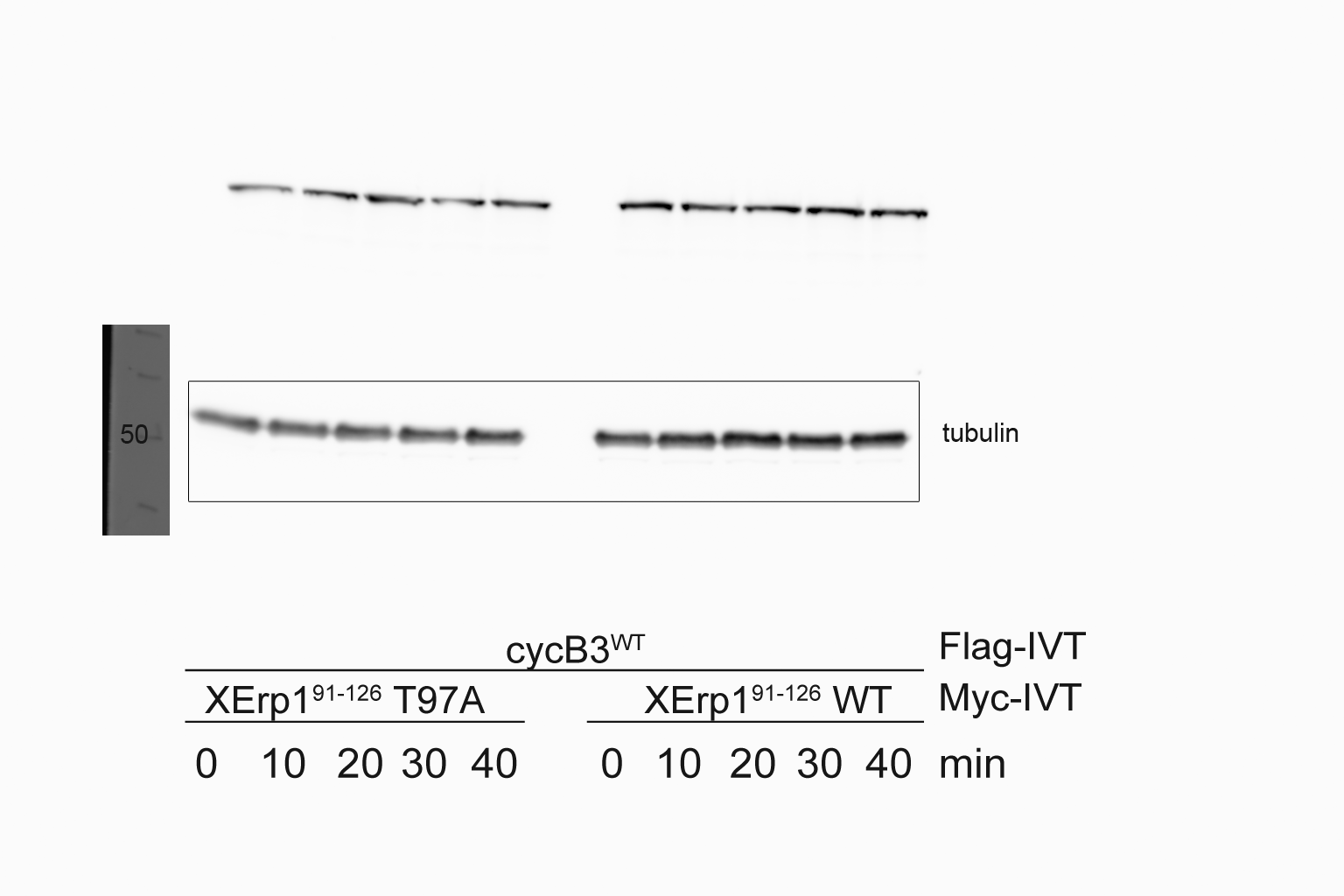

Supplement: Supplementary file 5 — Source data Fig. 4 [file 44319_2024_347_MOESM5_ESM.zip › Figure 4/4D/Western tubulin.tif]

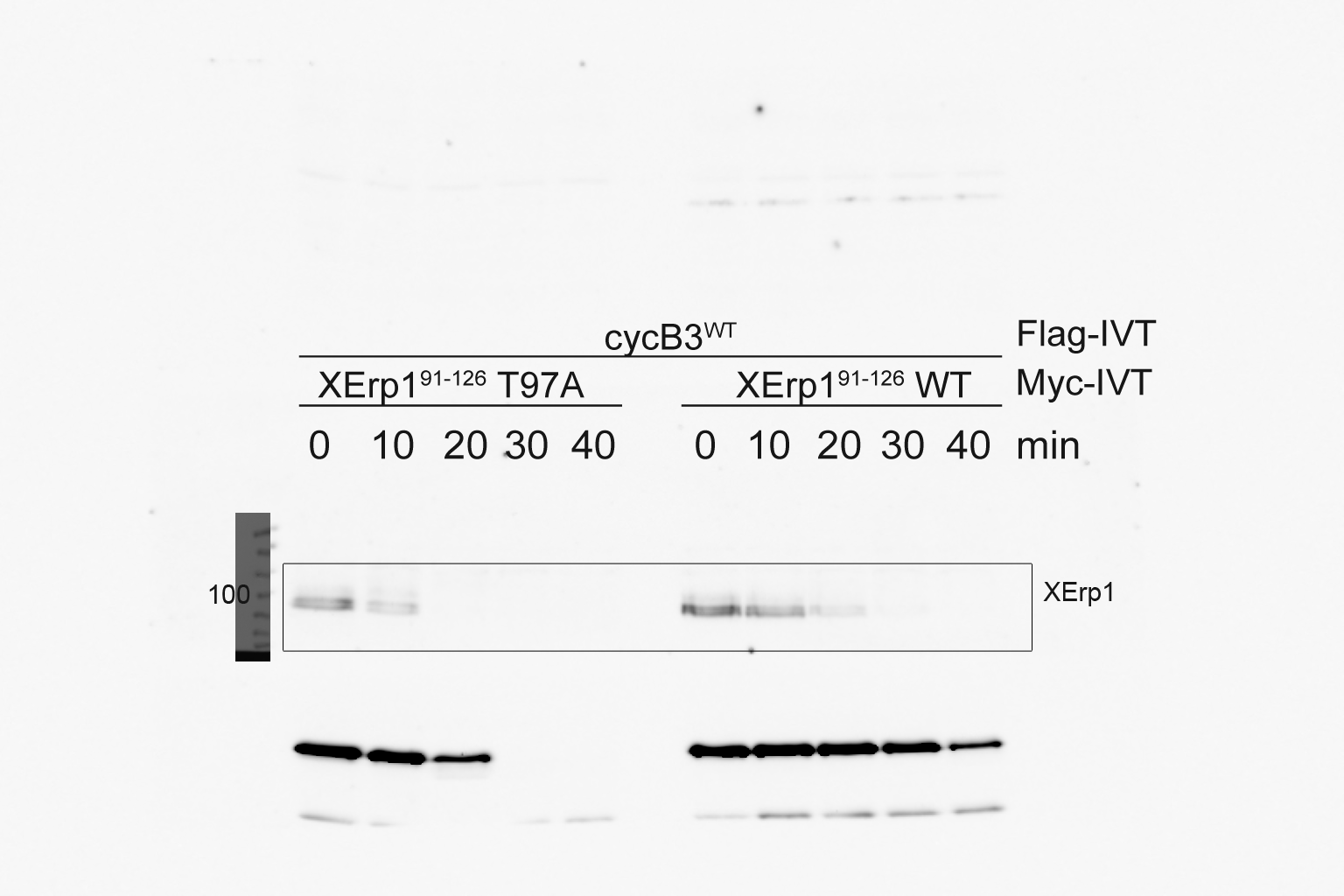

Supplement: Supplementary file 5 — Source data Fig. 4 [file 44319_2024_347_MOESM5_ESM.zip › Figure 4/4D/Western XErp1.tif]

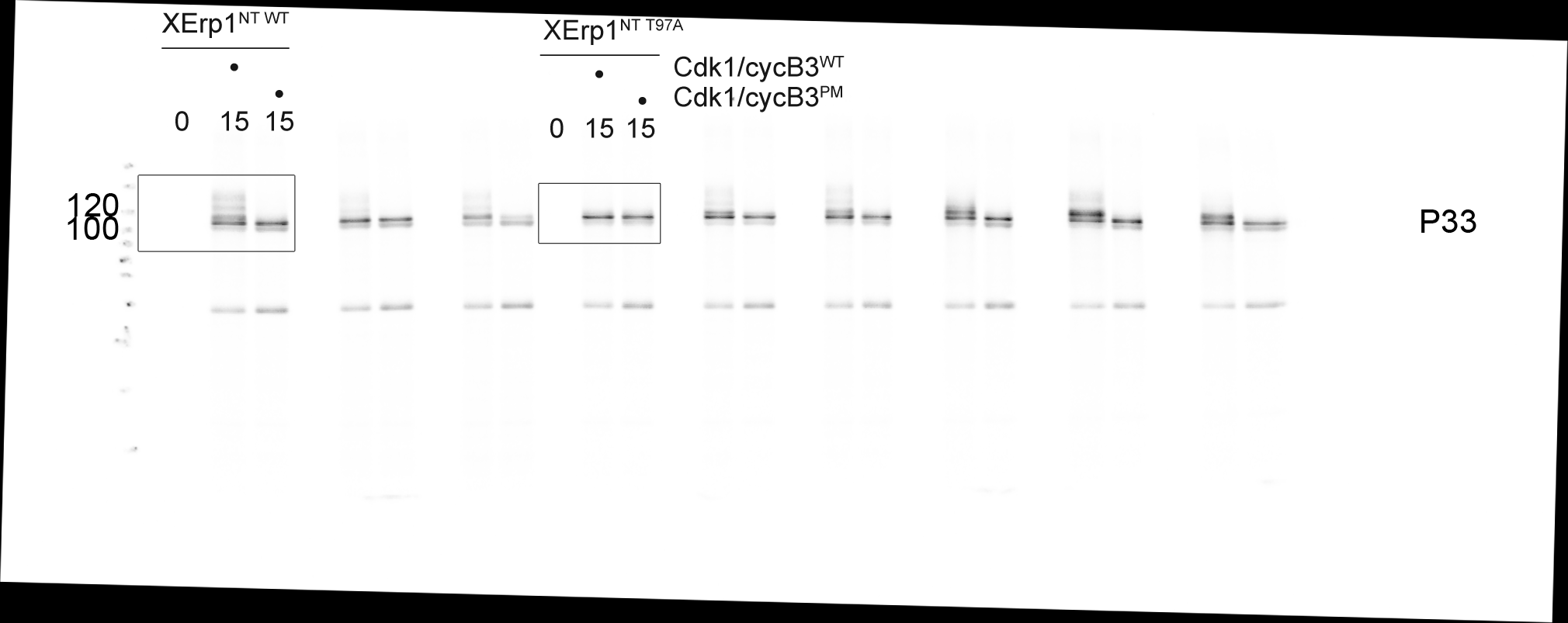

Supplement: Supplementary file 6 — Source data Fig. 5 [file 44319_2024_347_MOESM6_ESM.zip › Figure 5/5A/Autoradiogram P33.tif]

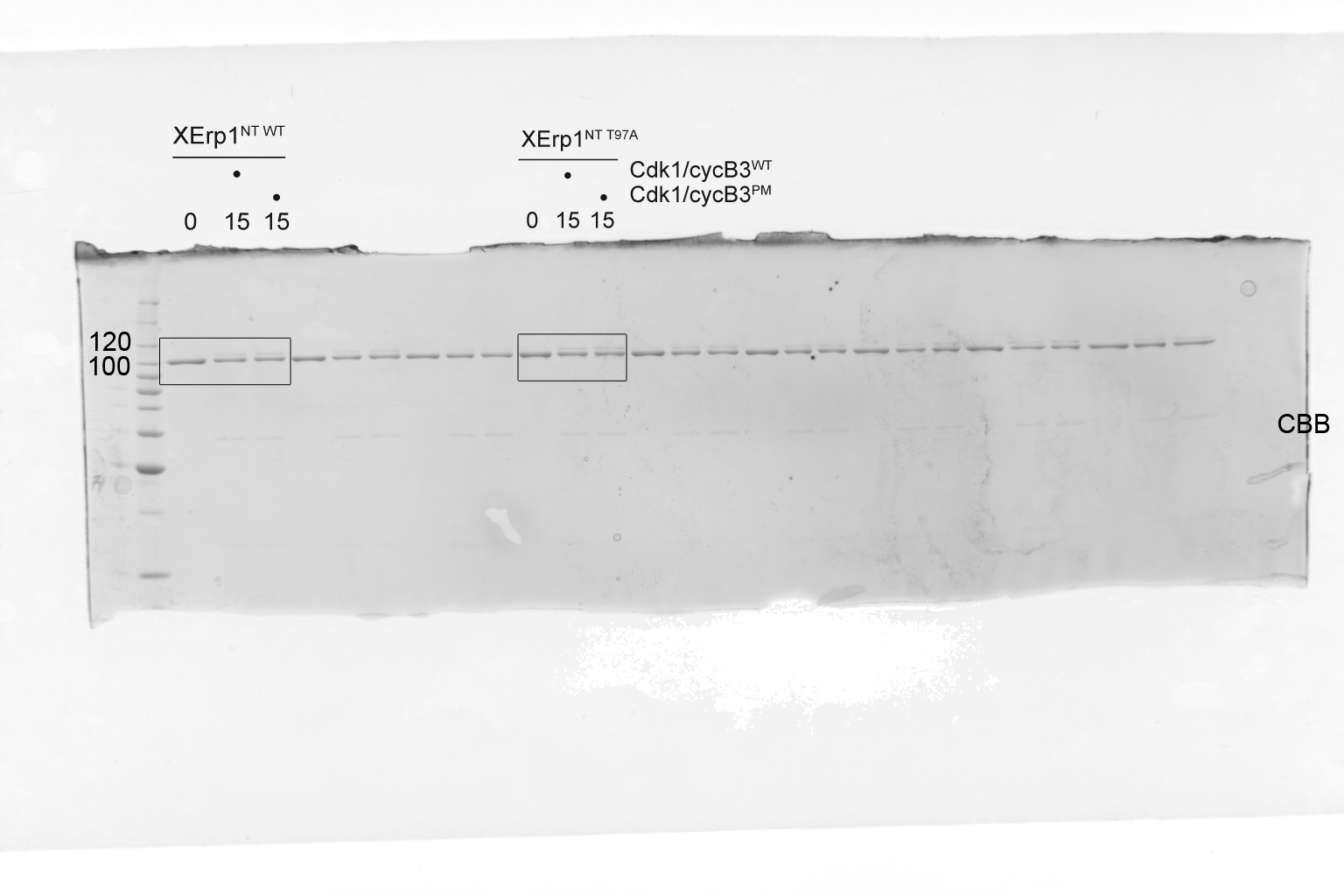

Supplement: Supplementary file 6 — Source data Fig. 5 [file 44319_2024_347_MOESM6_ESM.zip › Figure 5/5A/Coomassie CBB.tif]

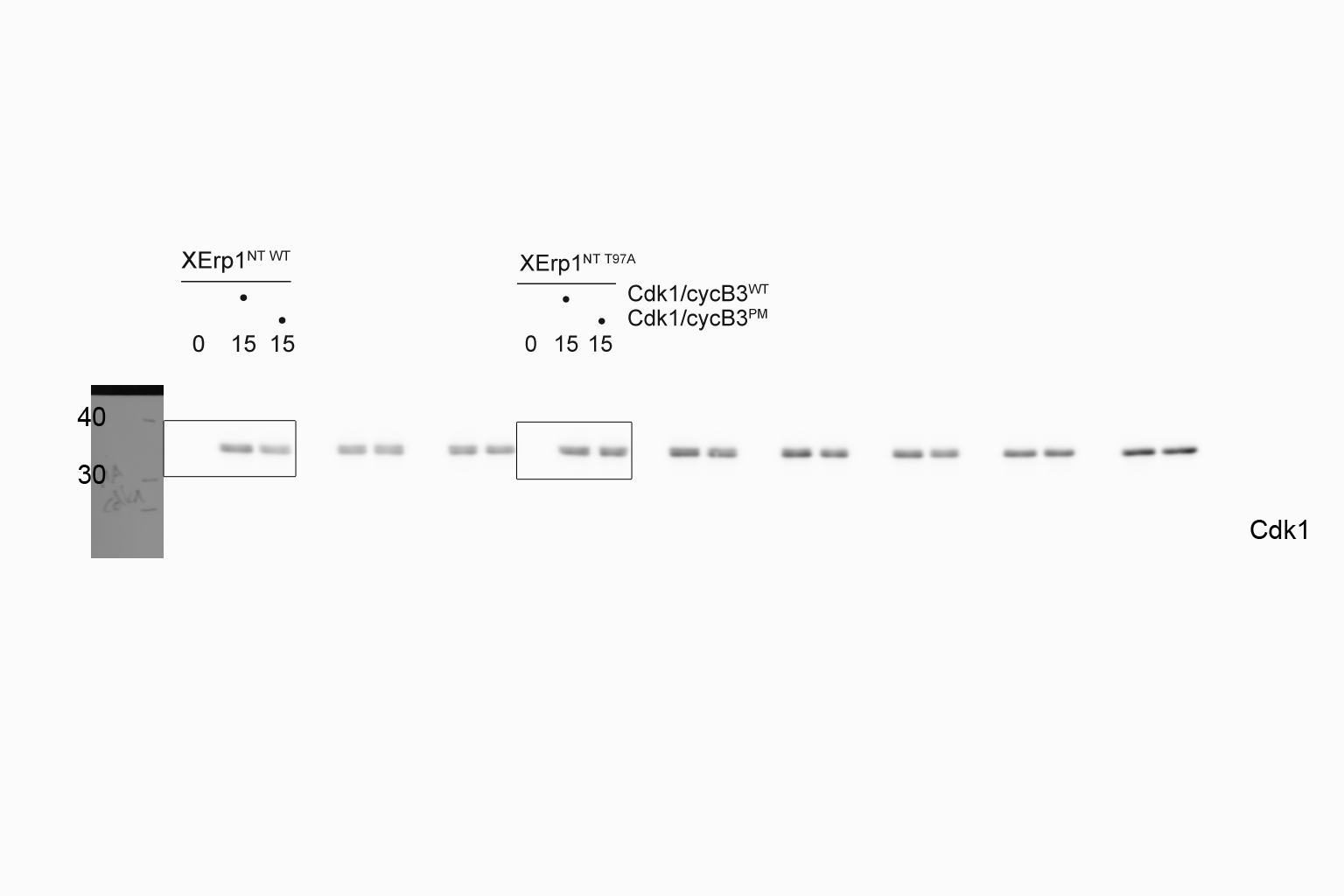

Supplement: Supplementary file 6 — Source data Fig. 5 [file 44319_2024_347_MOESM6_ESM.zip › Figure 5/5A/Western Cdk1.tif]

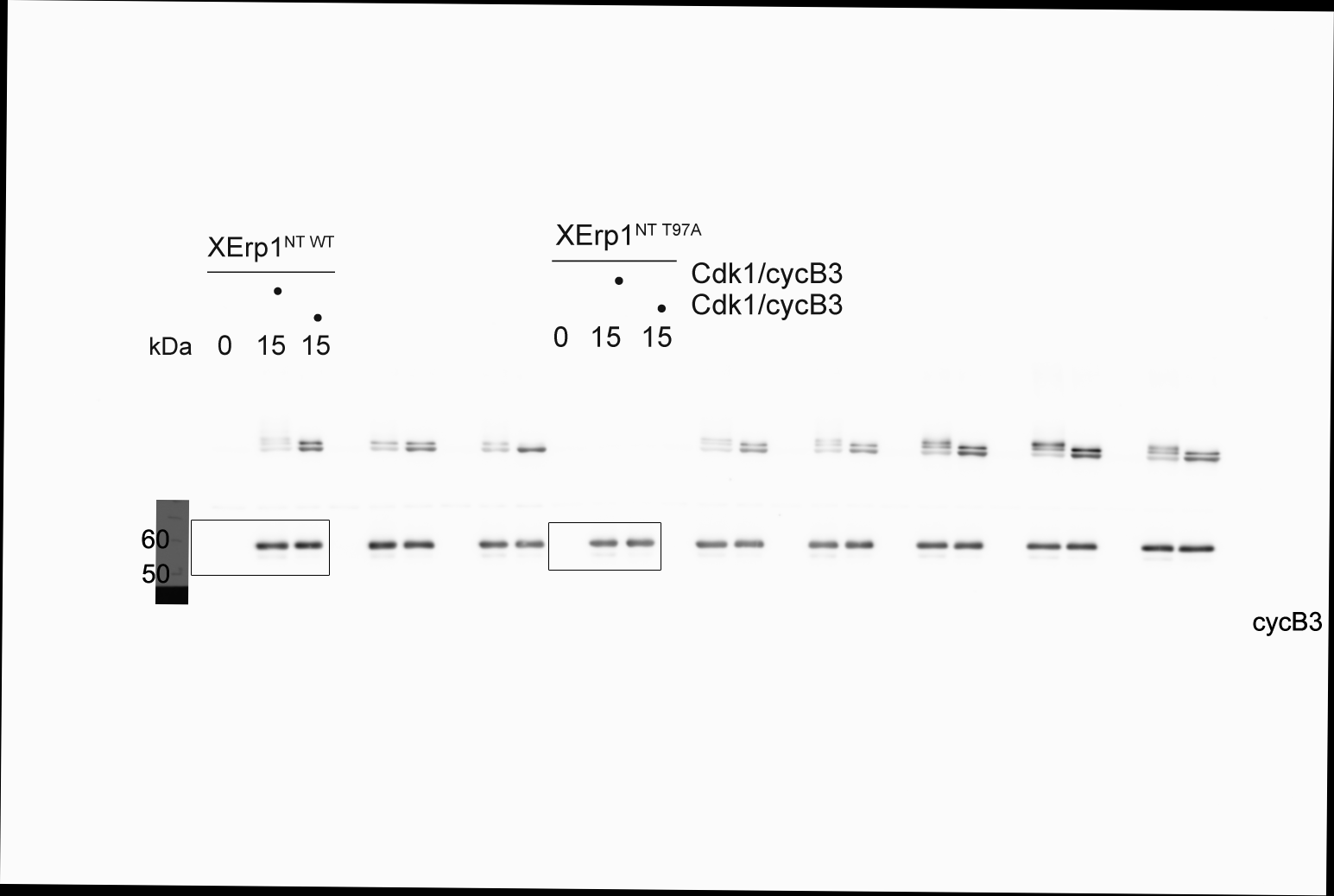

Supplement: Supplementary file 6 — Source data Fig. 5 [file 44319_2024_347_MOESM6_ESM.zip › Figure 5/5A/Western cycB3.tif]

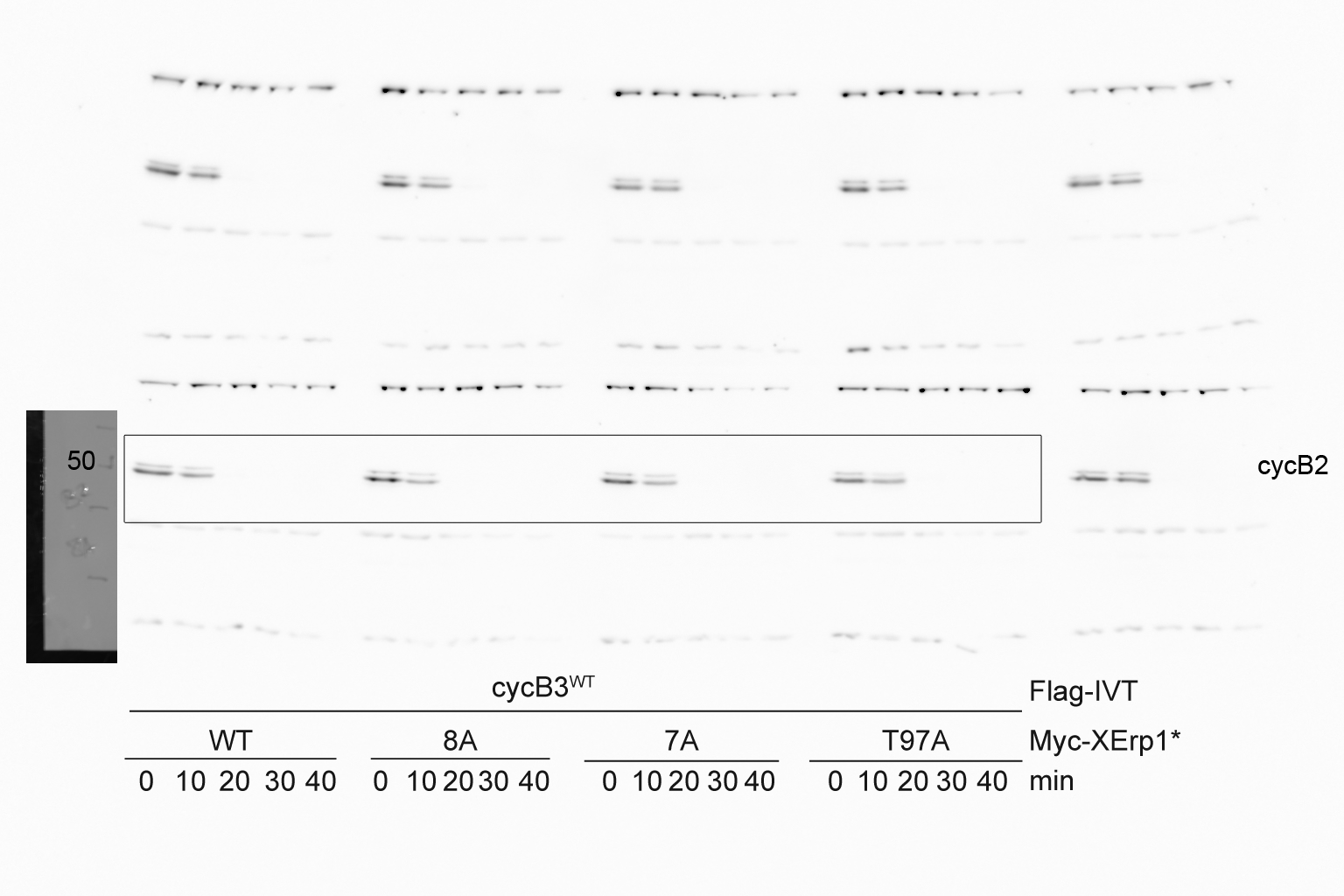

Supplement: Supplementary file 6 — Source data Fig. 5 [file 44319_2024_347_MOESM6_ESM.zip › Figure 5/5B/Western cycB2.tif]

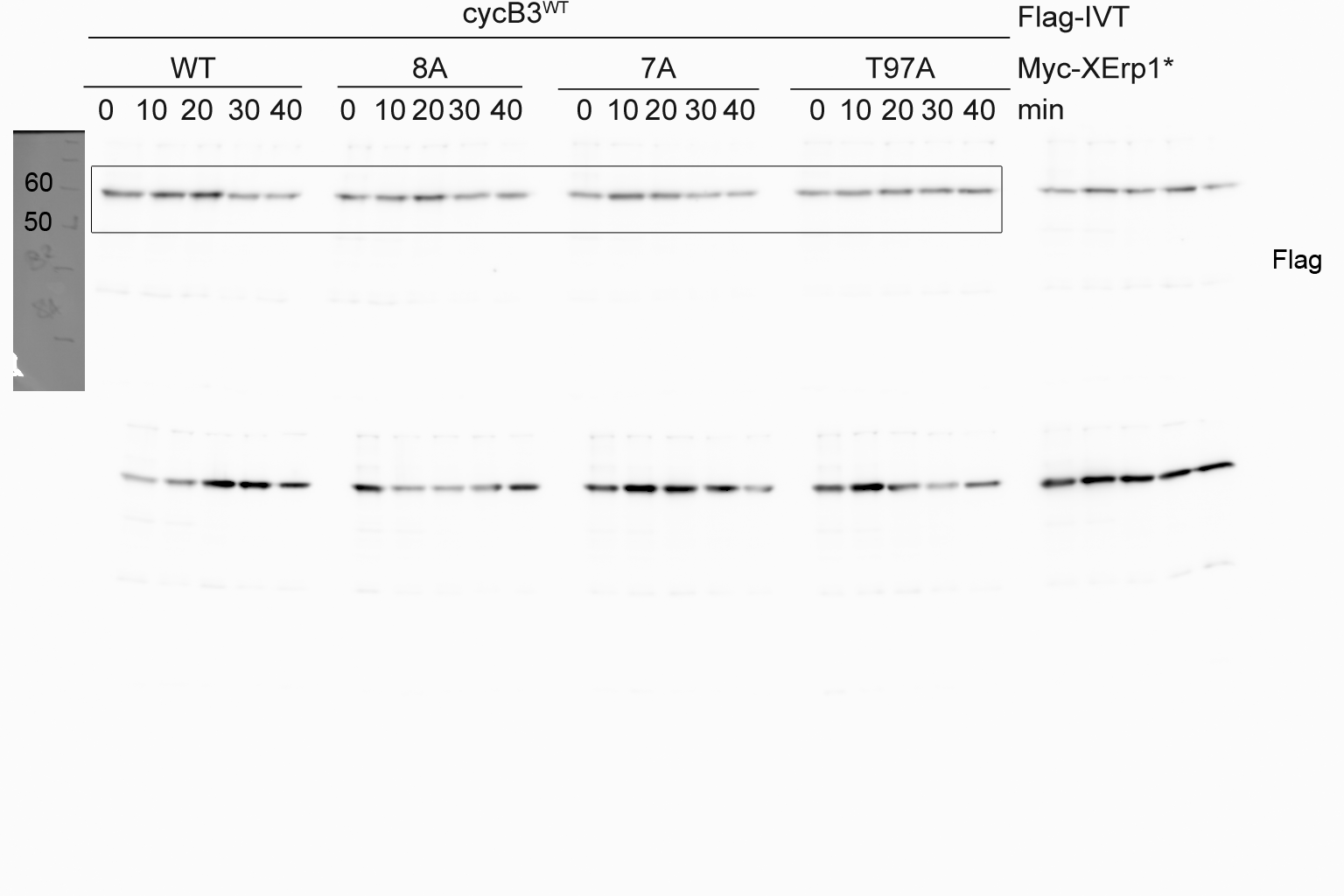

Supplement: Supplementary file 6 — Source data Fig. 5 [file 44319_2024_347_MOESM6_ESM.zip › Figure 5/5B/Western Flag.tif]

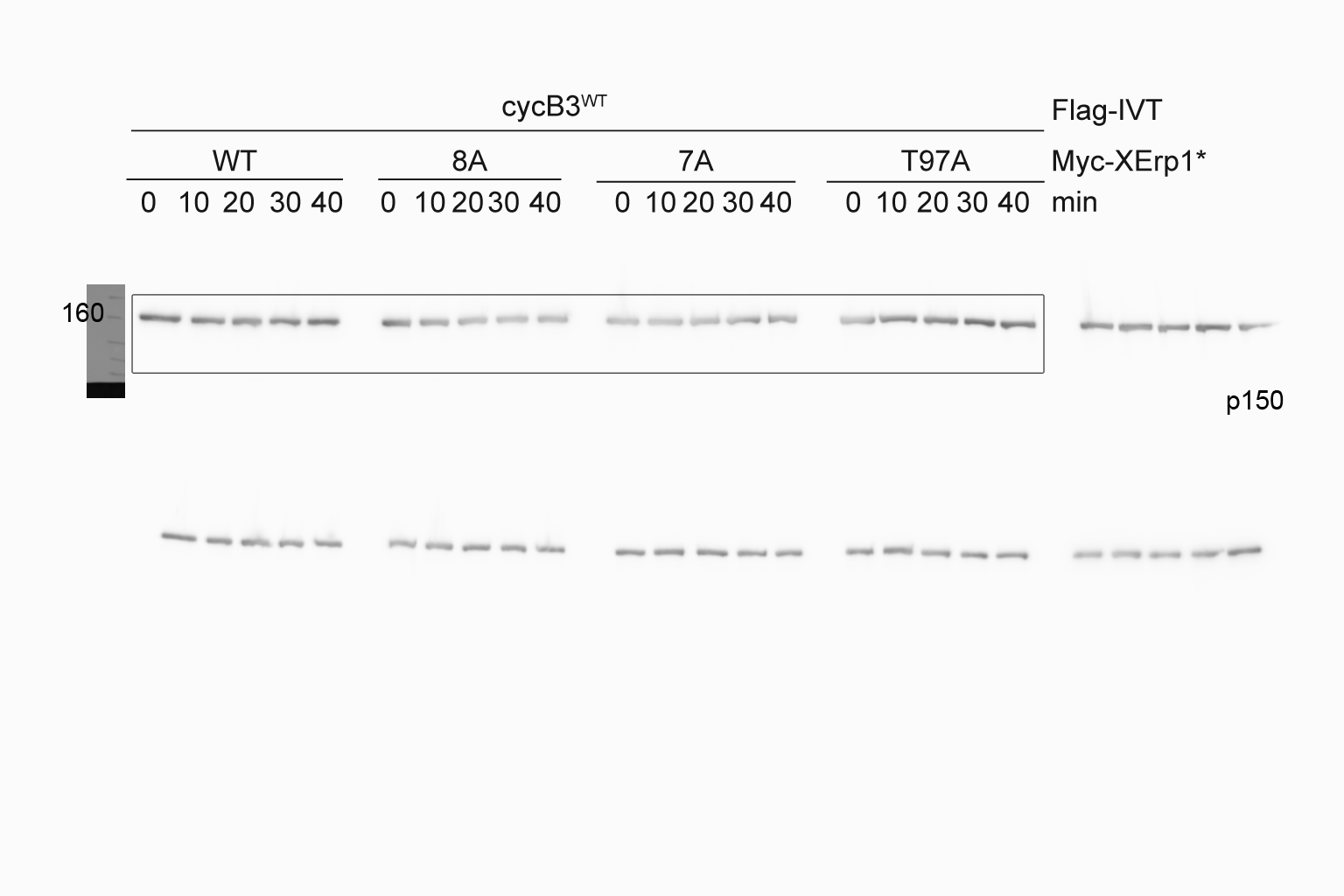

Supplement: Supplementary file 6 — Source data Fig. 5 [file 44319_2024_347_MOESM6_ESM.zip › Figure 5/5B/Western p150.tif]

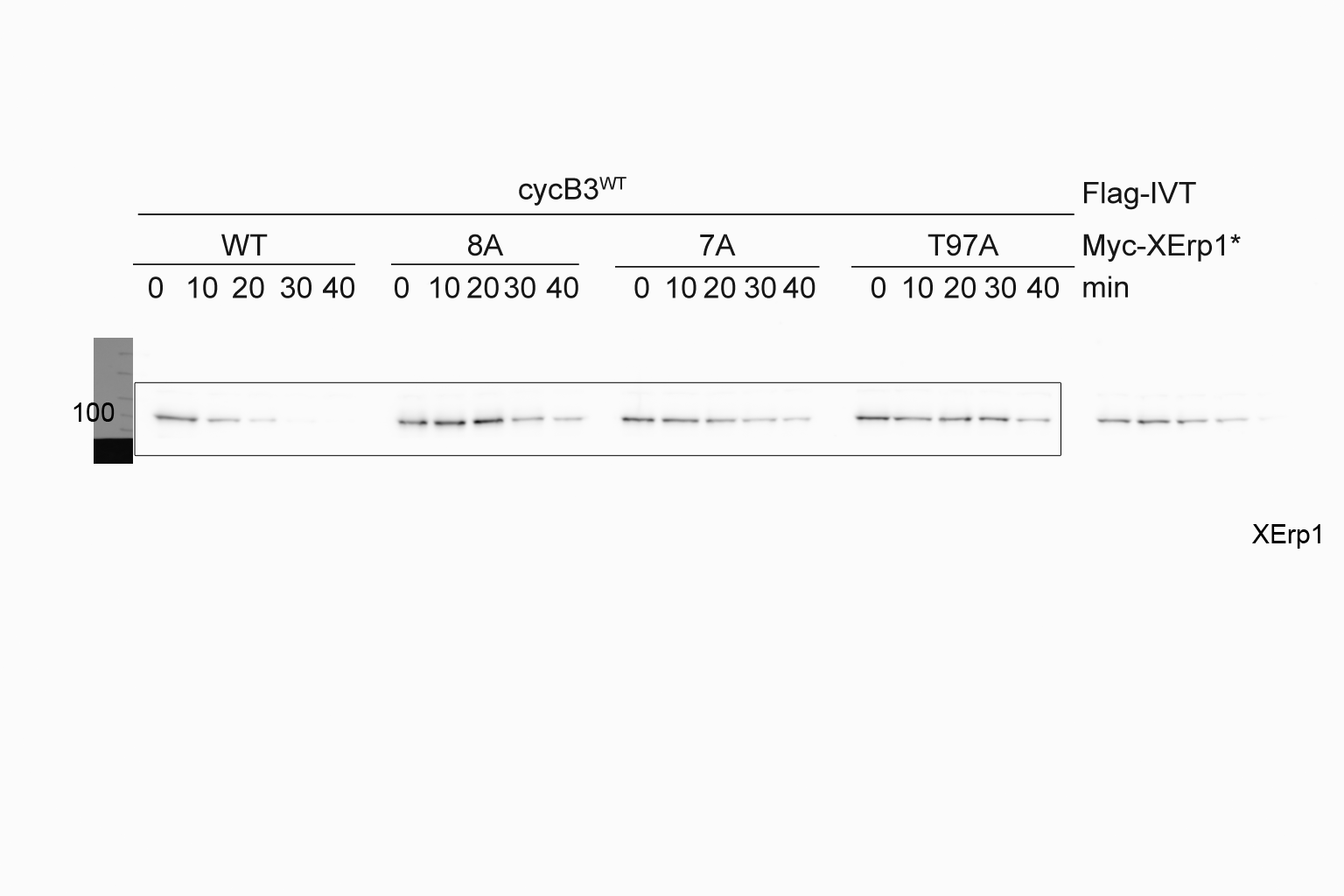

Supplement: Supplementary file 6 — Source data Fig. 5 [file 44319_2024_347_MOESM6_ESM.zip › Figure 5/5B/Western XErp1.tif]

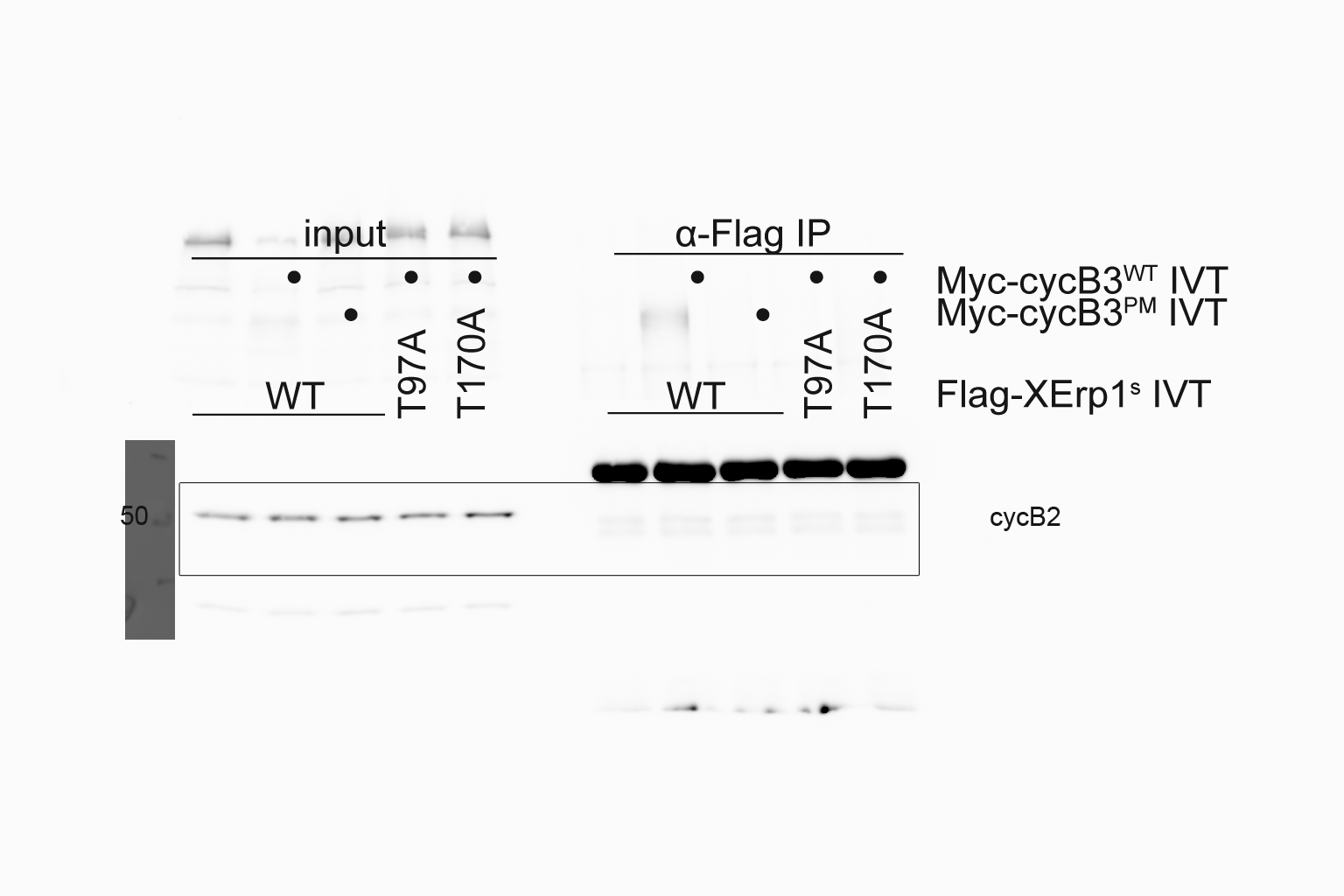

Supplement: Supplementary file 6 — Source data Fig. 5 [file 44319_2024_347_MOESM6_ESM.zip › Figure 5/5C/Western cycB2.tif]

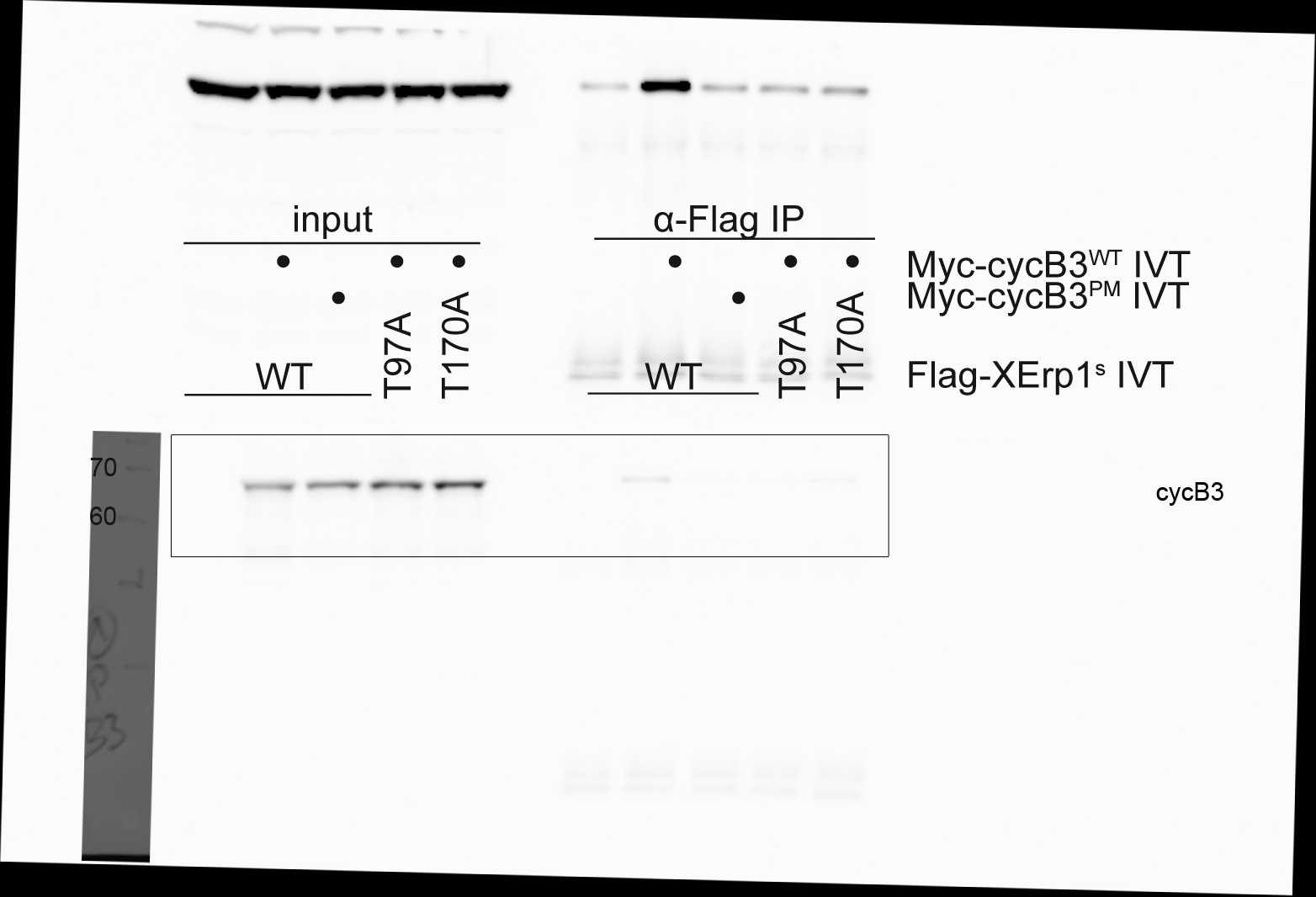

Supplement: Supplementary file 6 — Source data Fig. 5 [file 44319_2024_347_MOESM6_ESM.zip › Figure 5/5C/Western cycB3.tif]

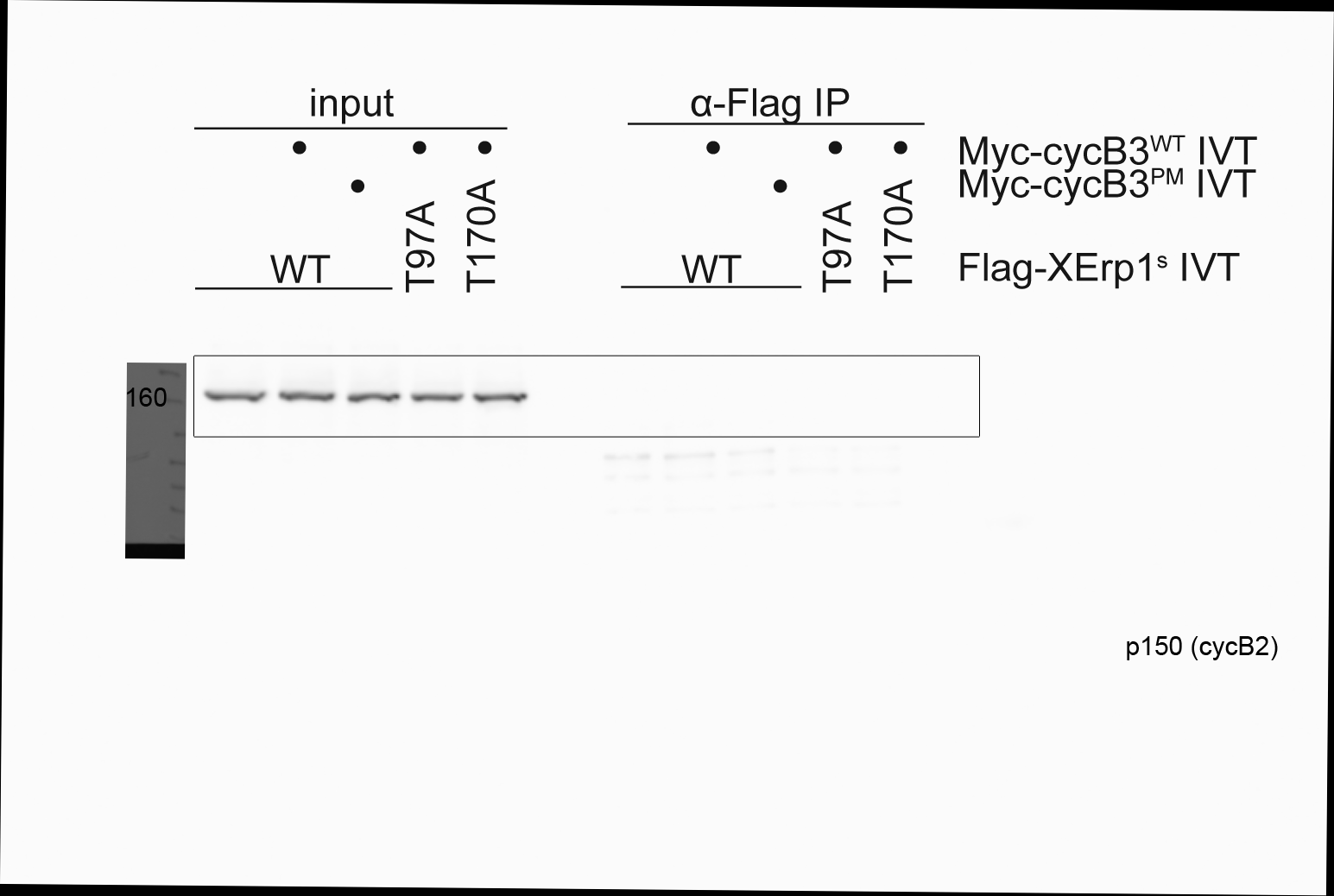

Supplement: Supplementary file 6 — Source data Fig. 5 [file 44319_2024_347_MOESM6_ESM.zip › Figure 5/5C/Western p150 (cycB2).tif]

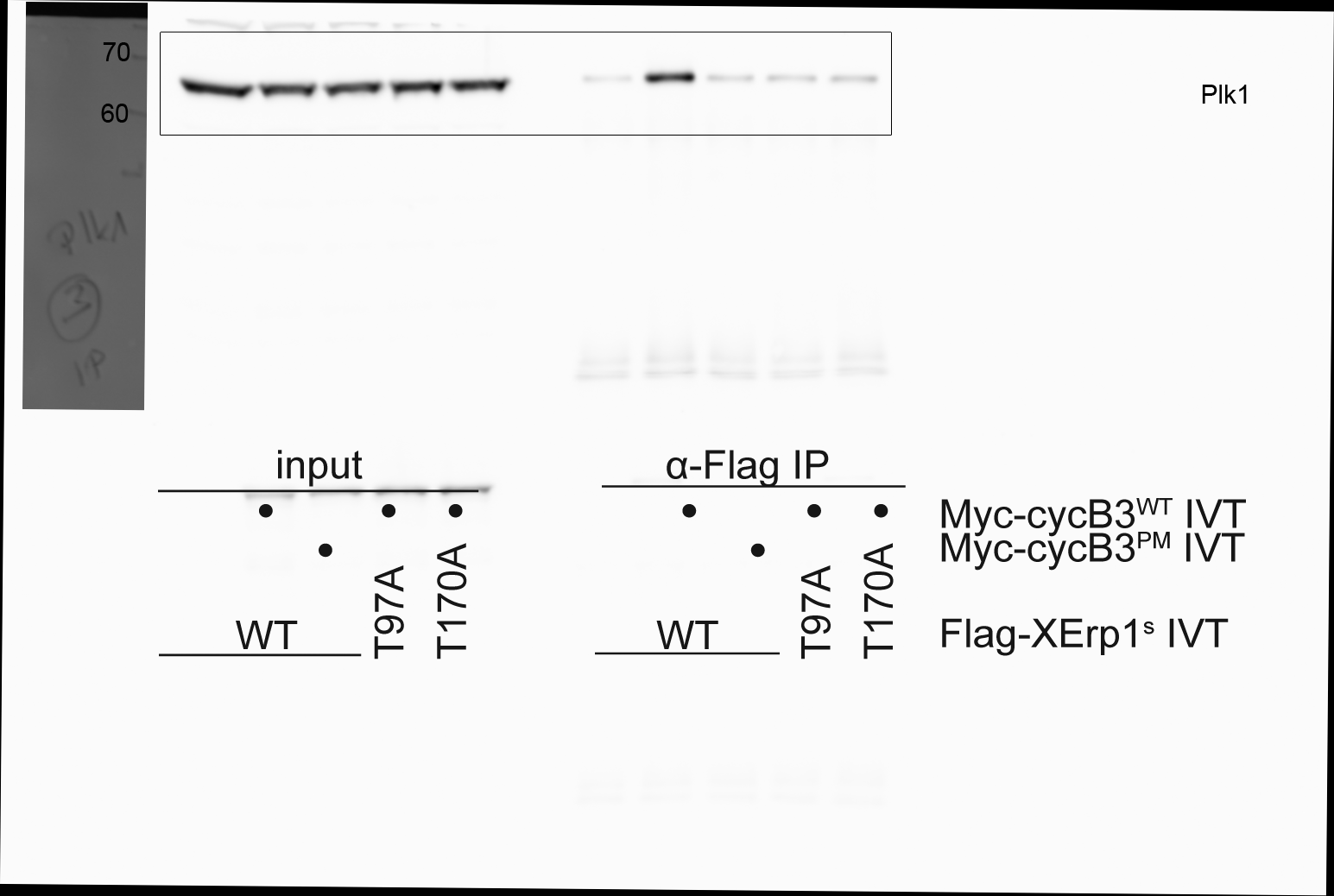

Supplement: Supplementary file 6 — Source data Fig. 5 [file 44319_2024_347_MOESM6_ESM.zip › Figure 5/5C/Western Plk1.tif]

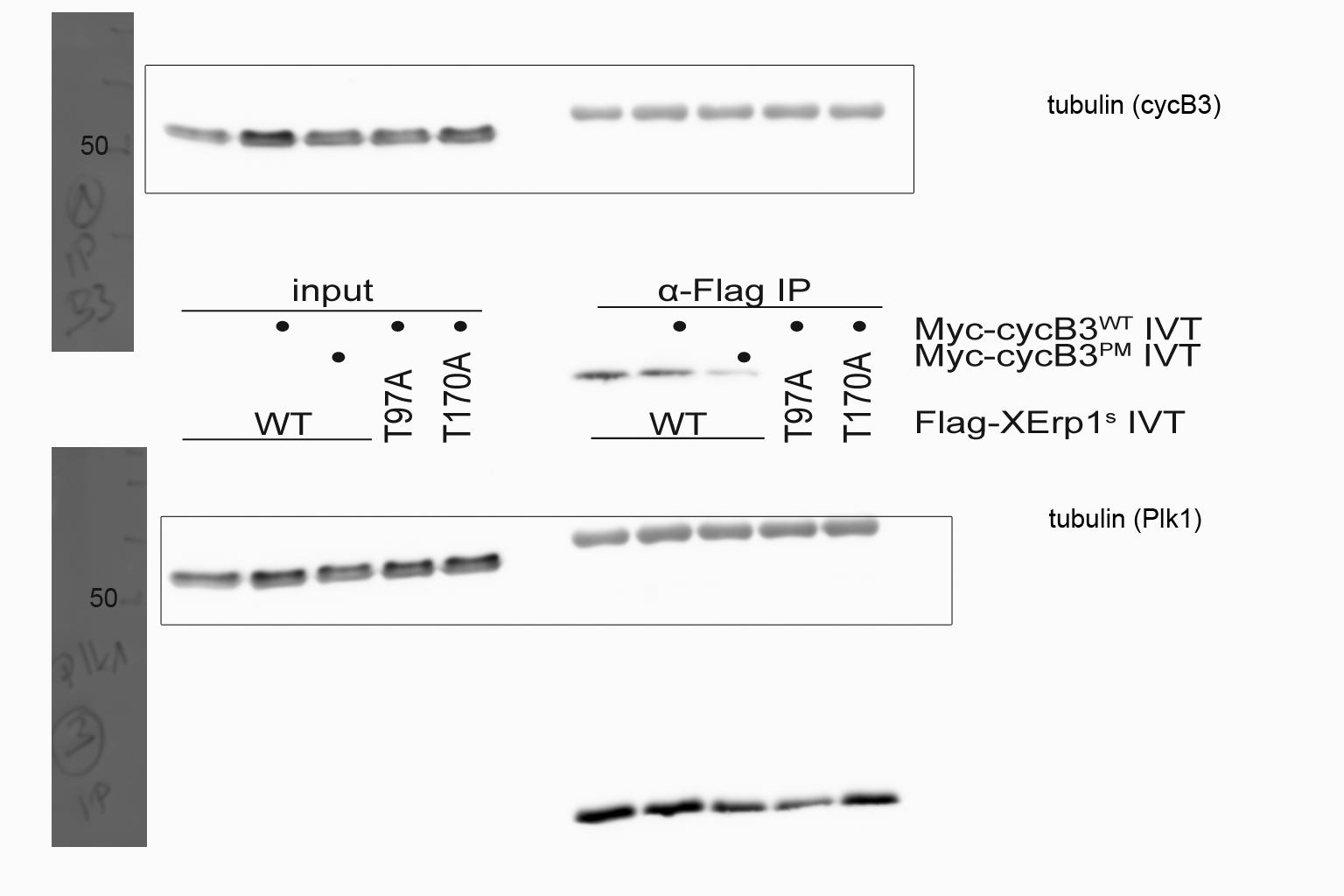

Supplement: Supplementary file 6 — Source data Fig. 5 [file 44319_2024_347_MOESM6_ESM.zip › Figure 5/5C/Western tubulin (cycB3), tubulin (Plk1).tif]

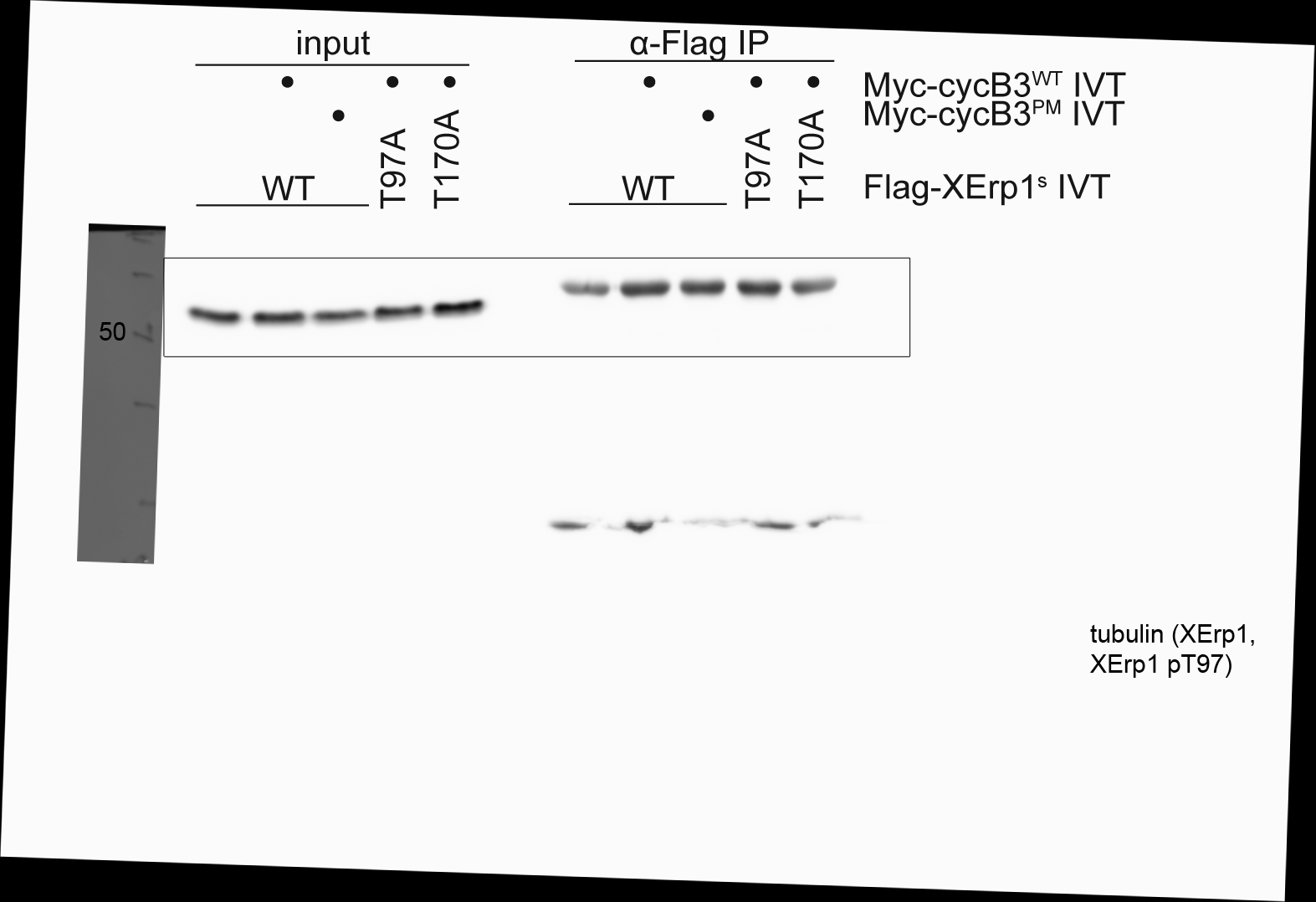

Supplement: Supplementary file 6 — Source data Fig. 5 [file 44319_2024_347_MOESM6_ESM.zip › Figure 5/5C/Western tubulin (XErp1).tif]

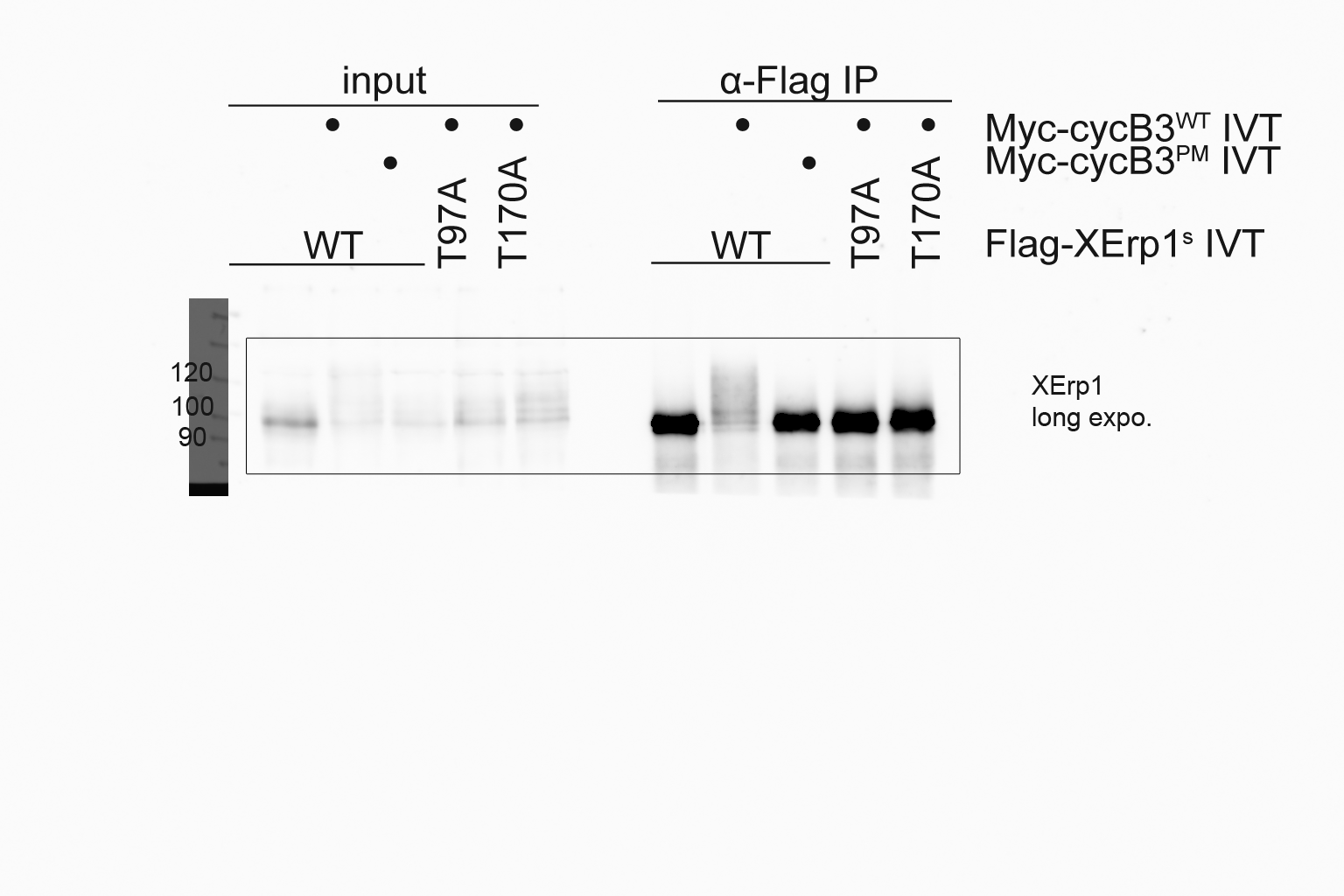

Supplement: Supplementary file 6 — Source data Fig. 5 [file 44319_2024_347_MOESM6_ESM.zip › Figure 5/5C/Western XErp1 long exposure.tif]

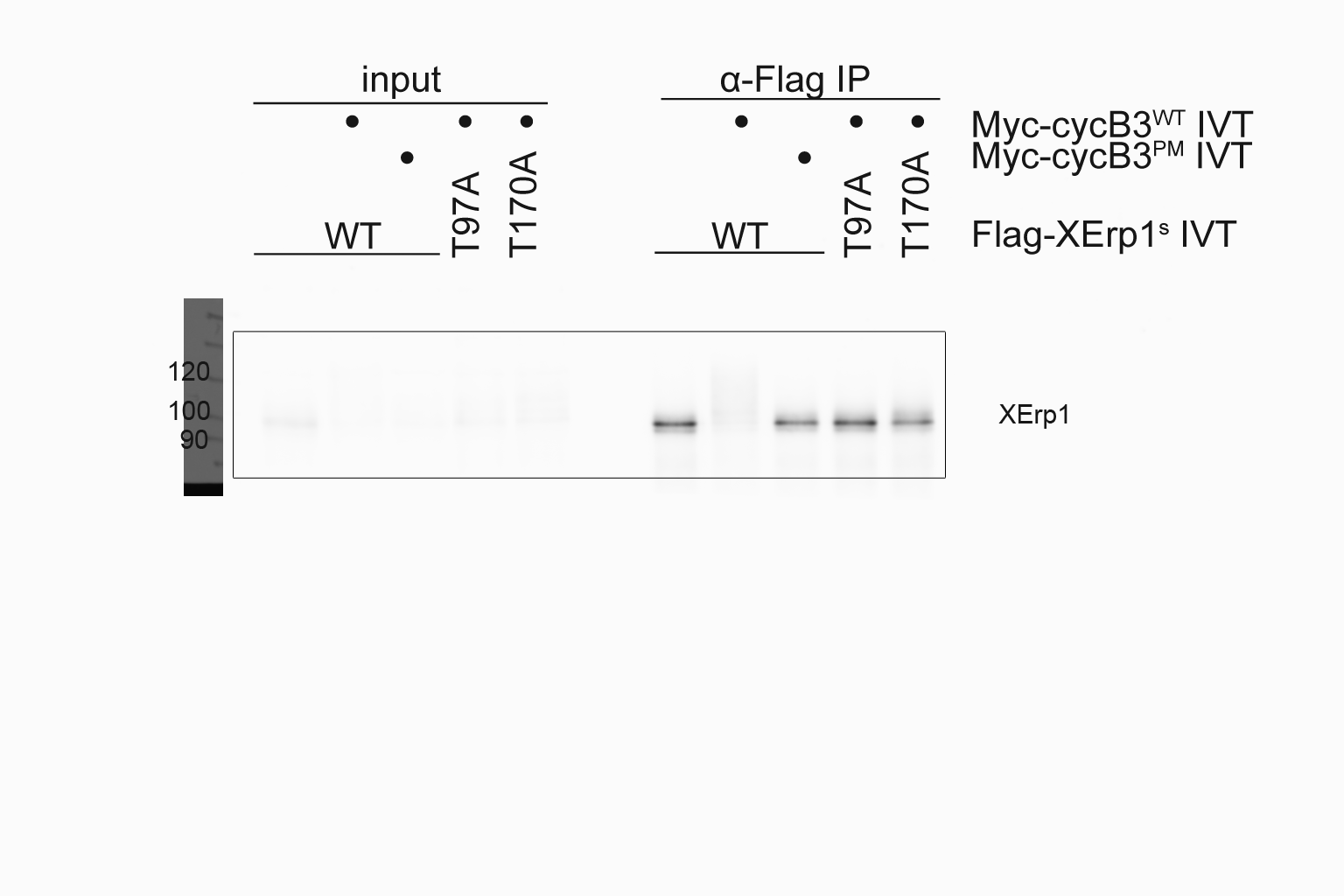

Supplement: Supplementary file 6 — Source data Fig. 5 [file 44319_2024_347_MOESM6_ESM.zip › Figure 5/5C/Western XErp1.tif]

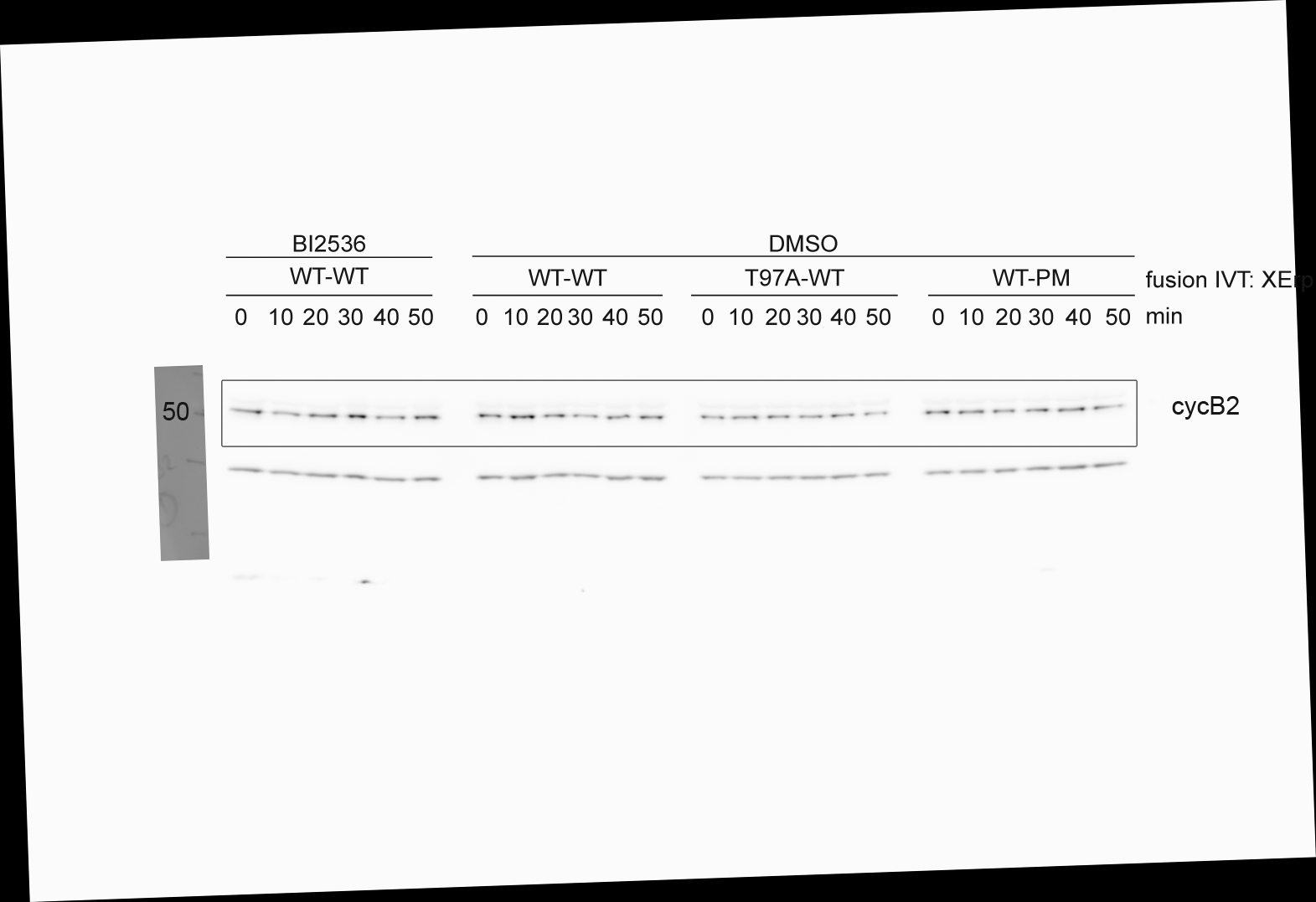

Supplement: Supplementary file 6 — Source data Fig. 5 [file 44319_2024_347_MOESM6_ESM.zip › Figure 5/5D/Western cycB2.tif]

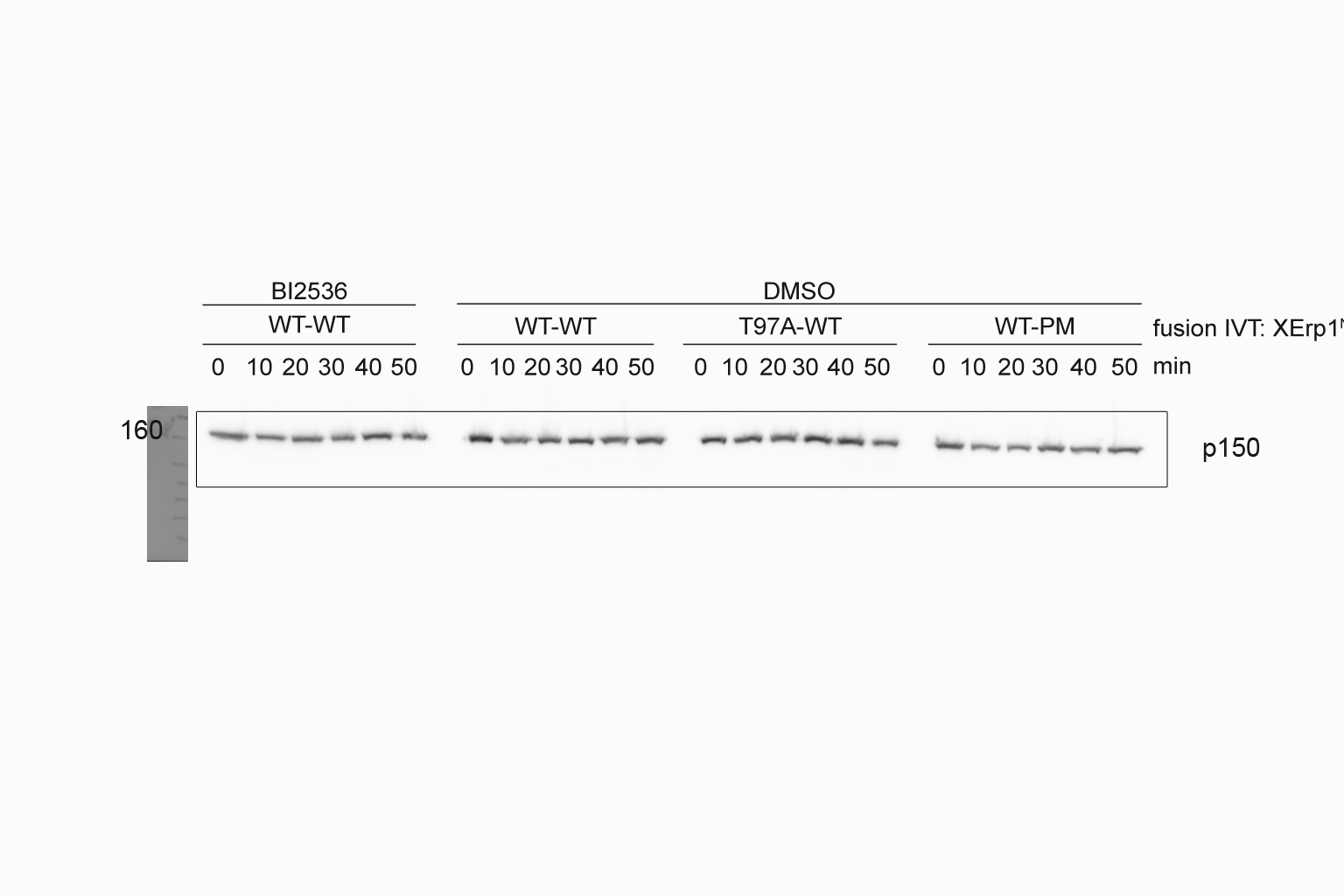

Supplement: Supplementary file 6 — Source data Fig. 5 [file 44319_2024_347_MOESM6_ESM.zip › Figure 5/5D/Western p150.tif]

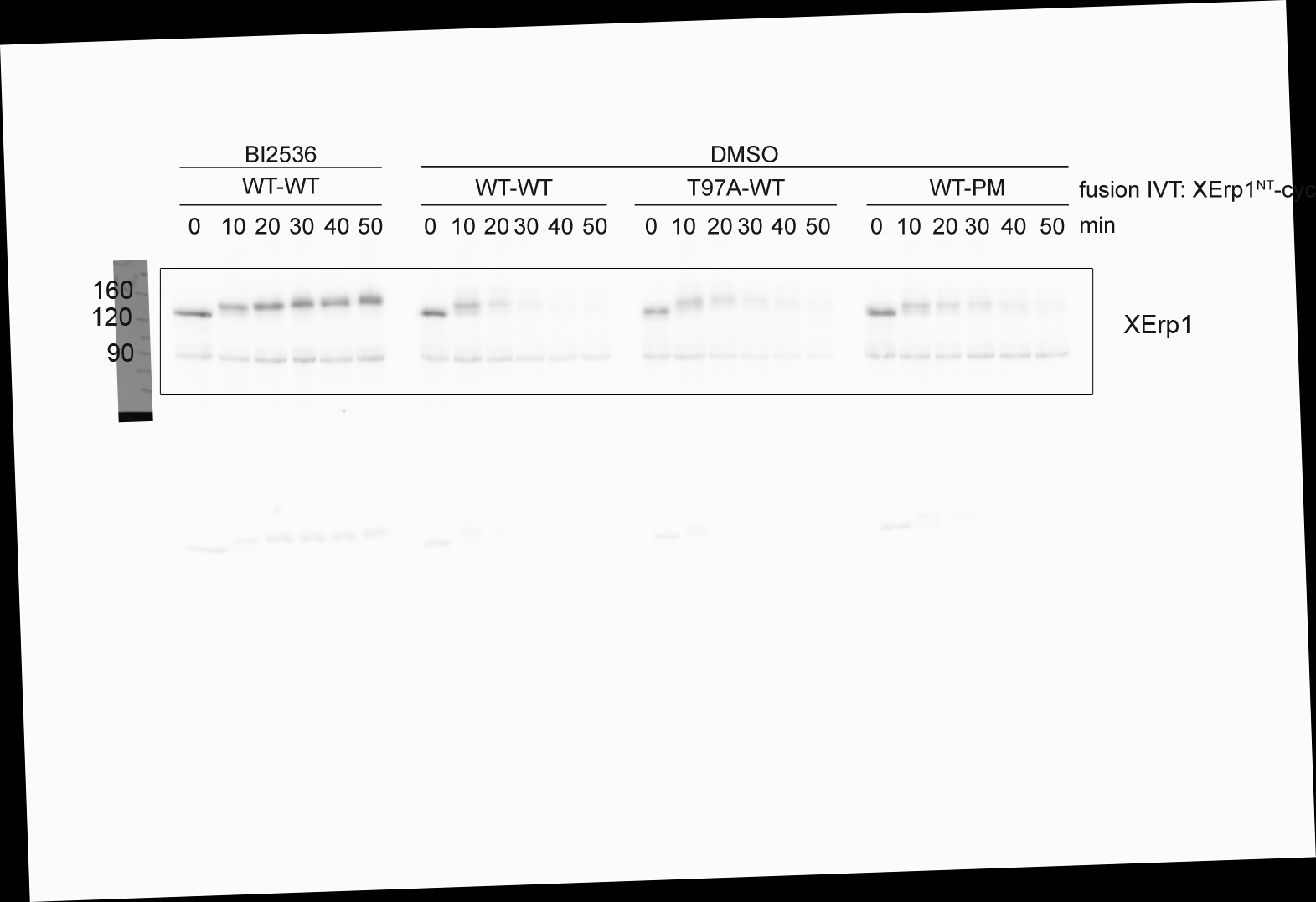

Supplement: Supplementary file 6 — Source data Fig. 5 [file 44319_2024_347_MOESM6_ESM.zip › Figure 5/5D/Western XErp1.tif]

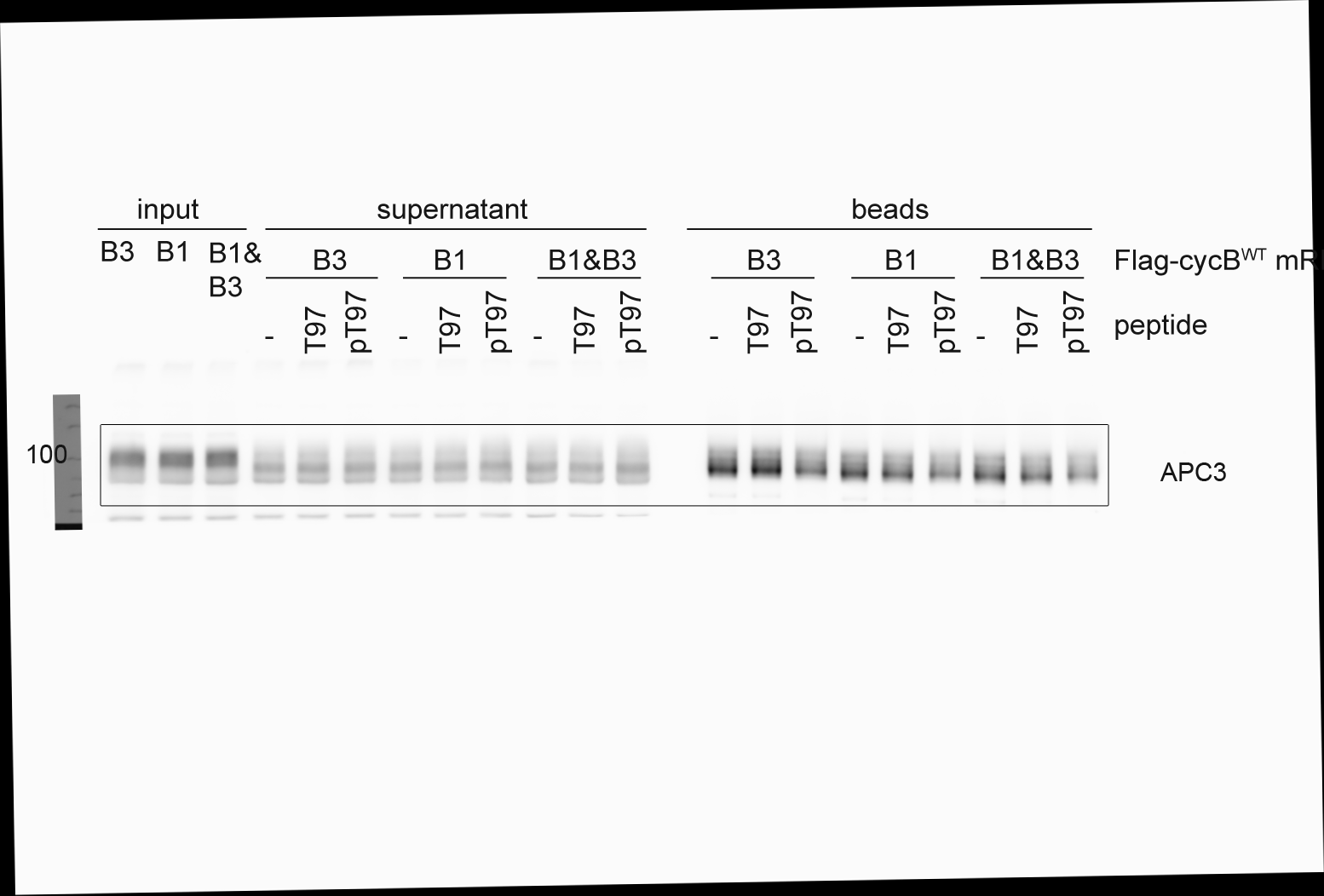

Supplement: Supplementary file 7 — Source data Fig. 6 [file 44319_2024_347_MOESM7_ESM.zip › Figure 6/6A/Western APC3.tif]

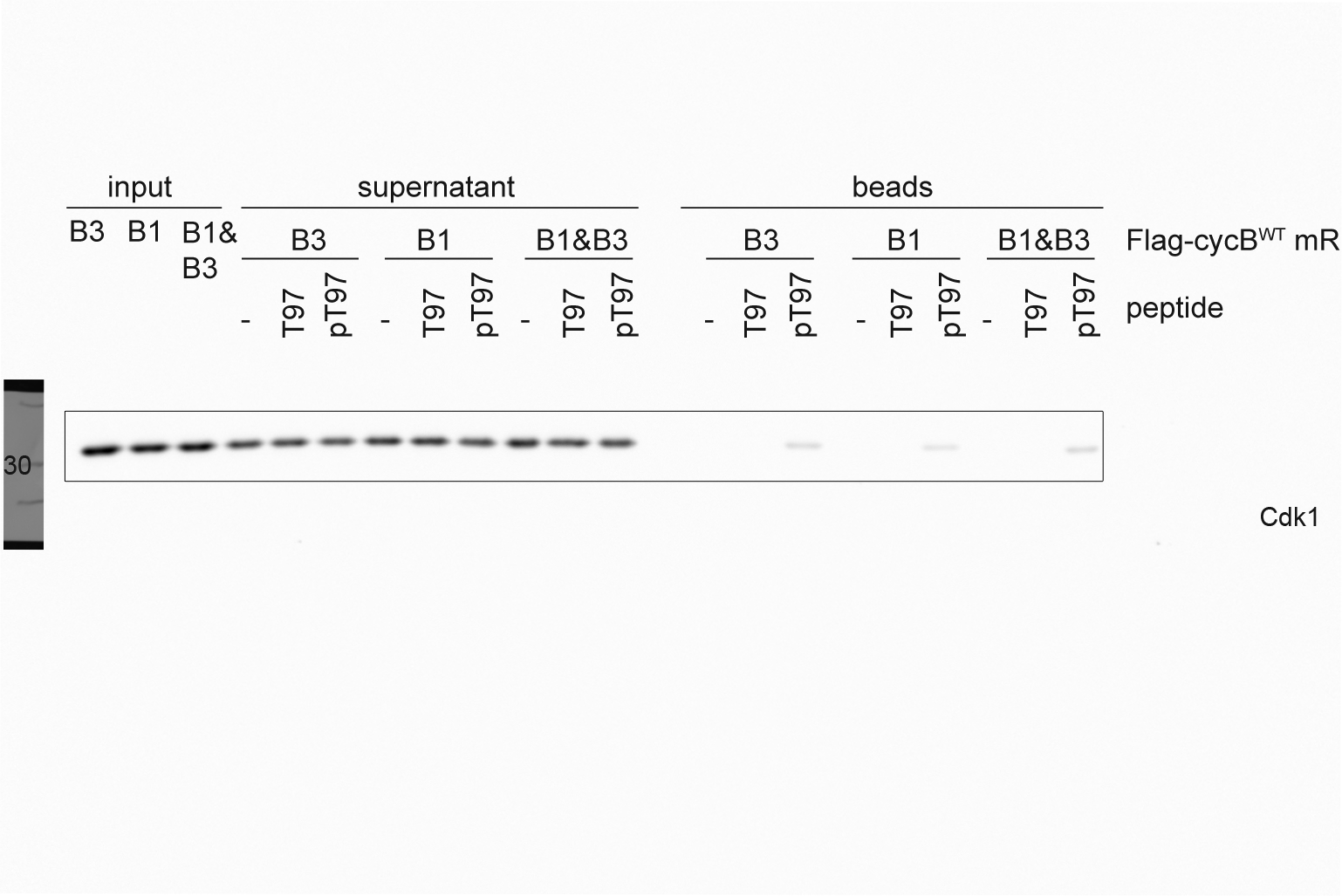

Supplement: Supplementary file 7 — Source data Fig. 6 [file 44319_2024_347_MOESM7_ESM.zip › Figure 6/6A/Western Cdk1.tif]

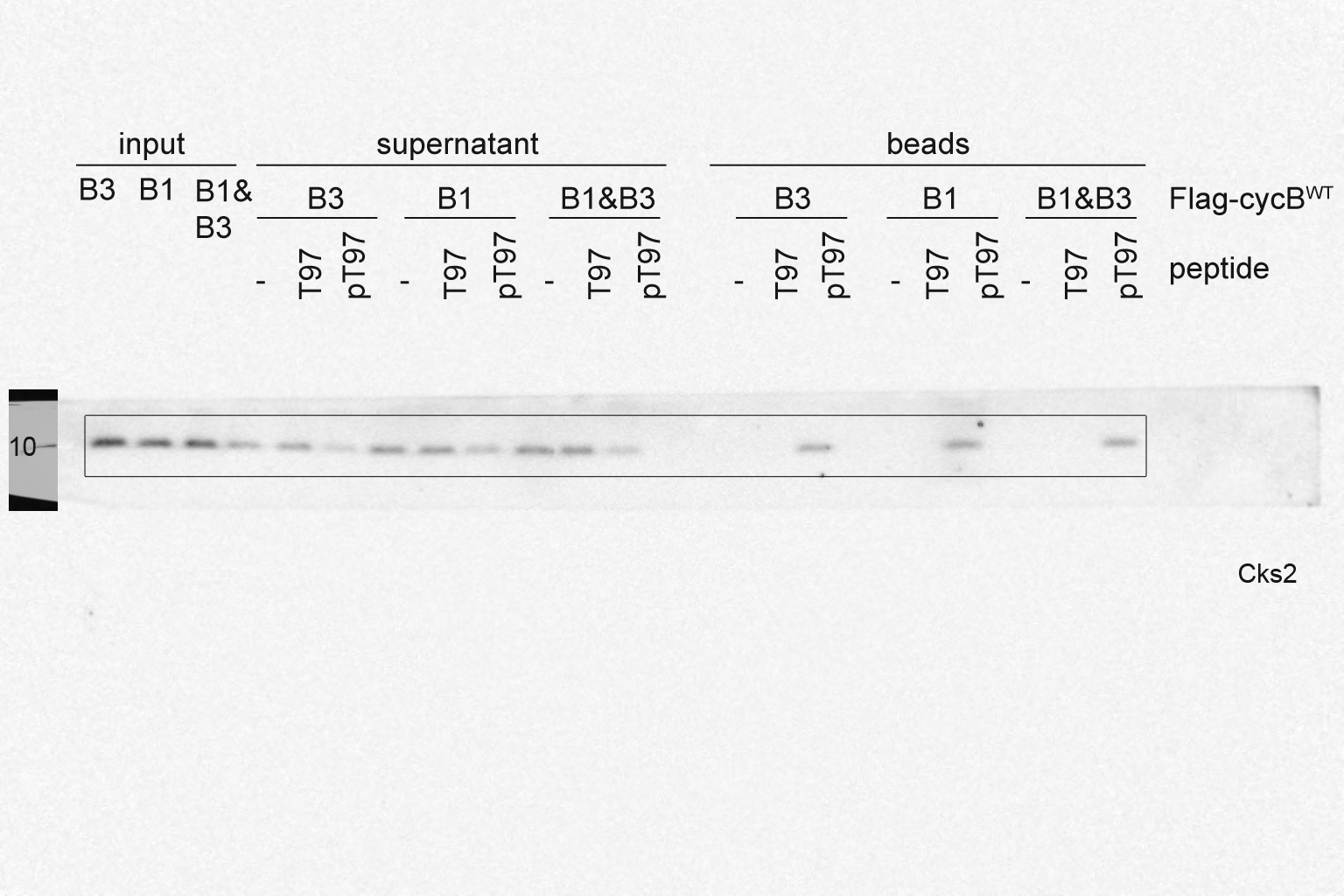

Supplement: Supplementary file 7 — Source data Fig. 6 [file 44319_2024_347_MOESM7_ESM.zip › Figure 6/6A/Western Cks2.tif]

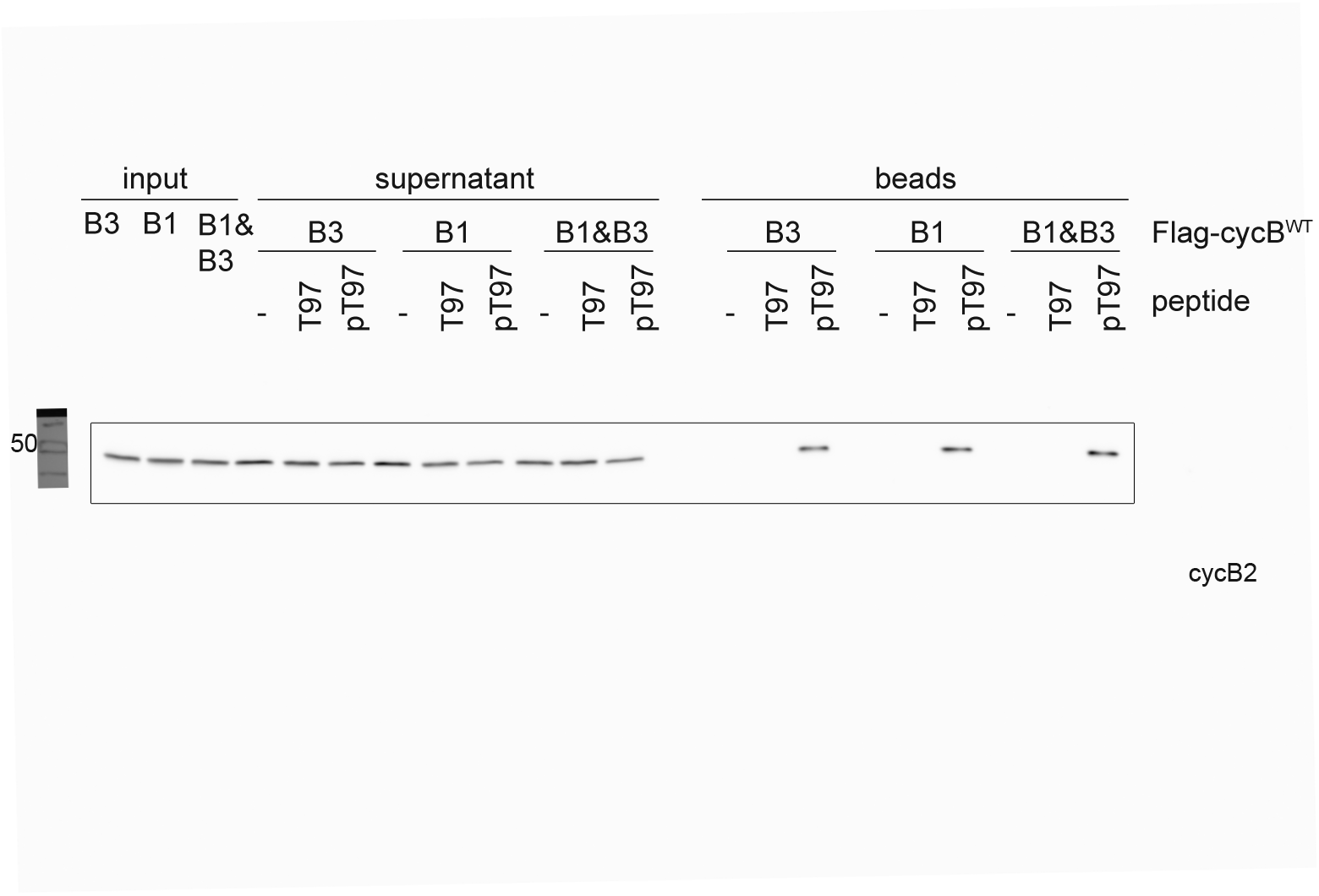

Supplement: Supplementary file 7 — Source data Fig. 6 [file 44319_2024_347_MOESM7_ESM.zip › Figure 6/6A/Western cycB2.tif]

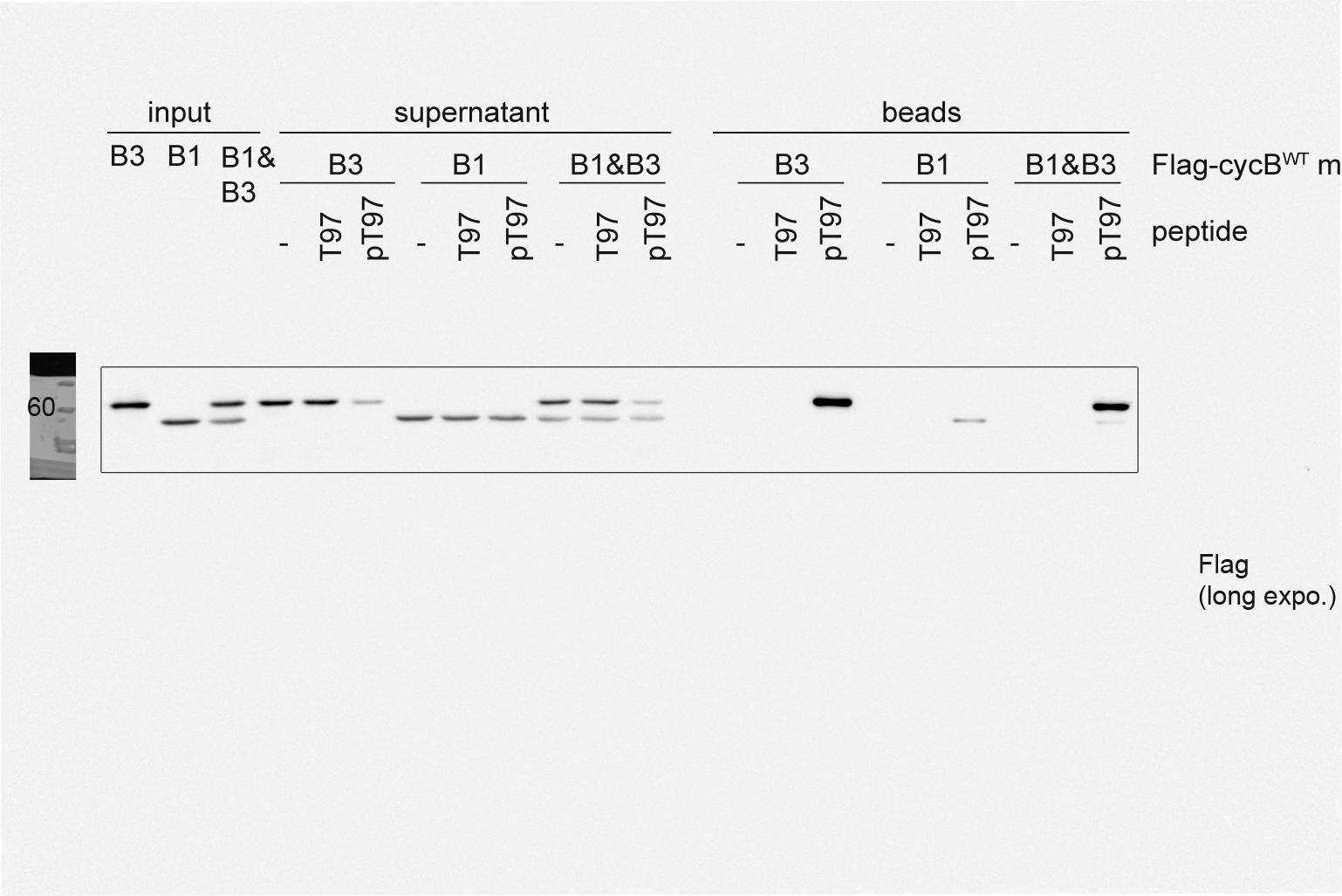

Supplement: Supplementary file 7 — Source data Fig. 6 [file 44319_2024_347_MOESM7_ESM.zip › Figure 6/6A/Western Flag (long exposure).tif]

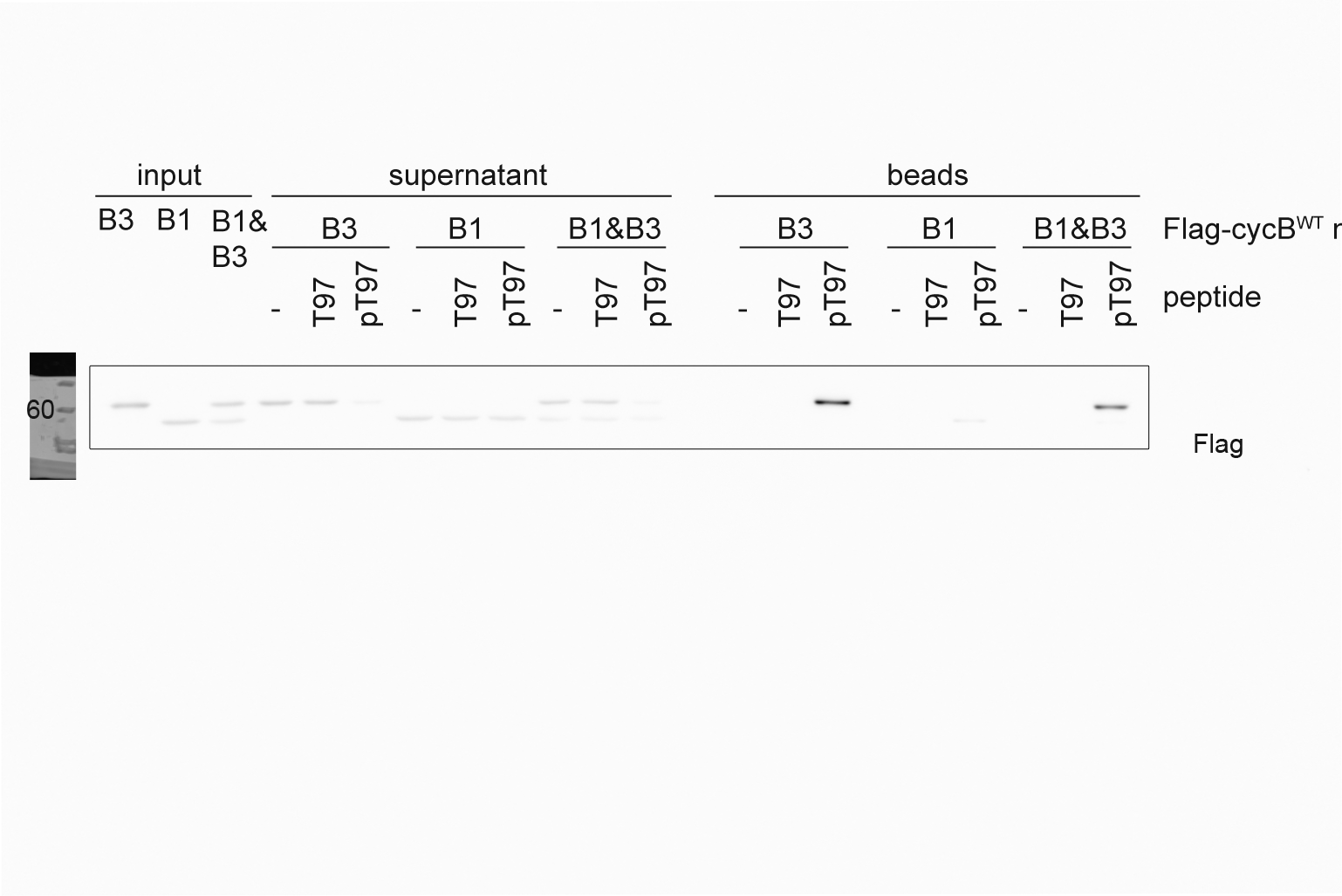

Supplement: Supplementary file 7 — Source data Fig. 6 [file 44319_2024_347_MOESM7_ESM.zip › Figure 6/6A/Western Flag.tif]

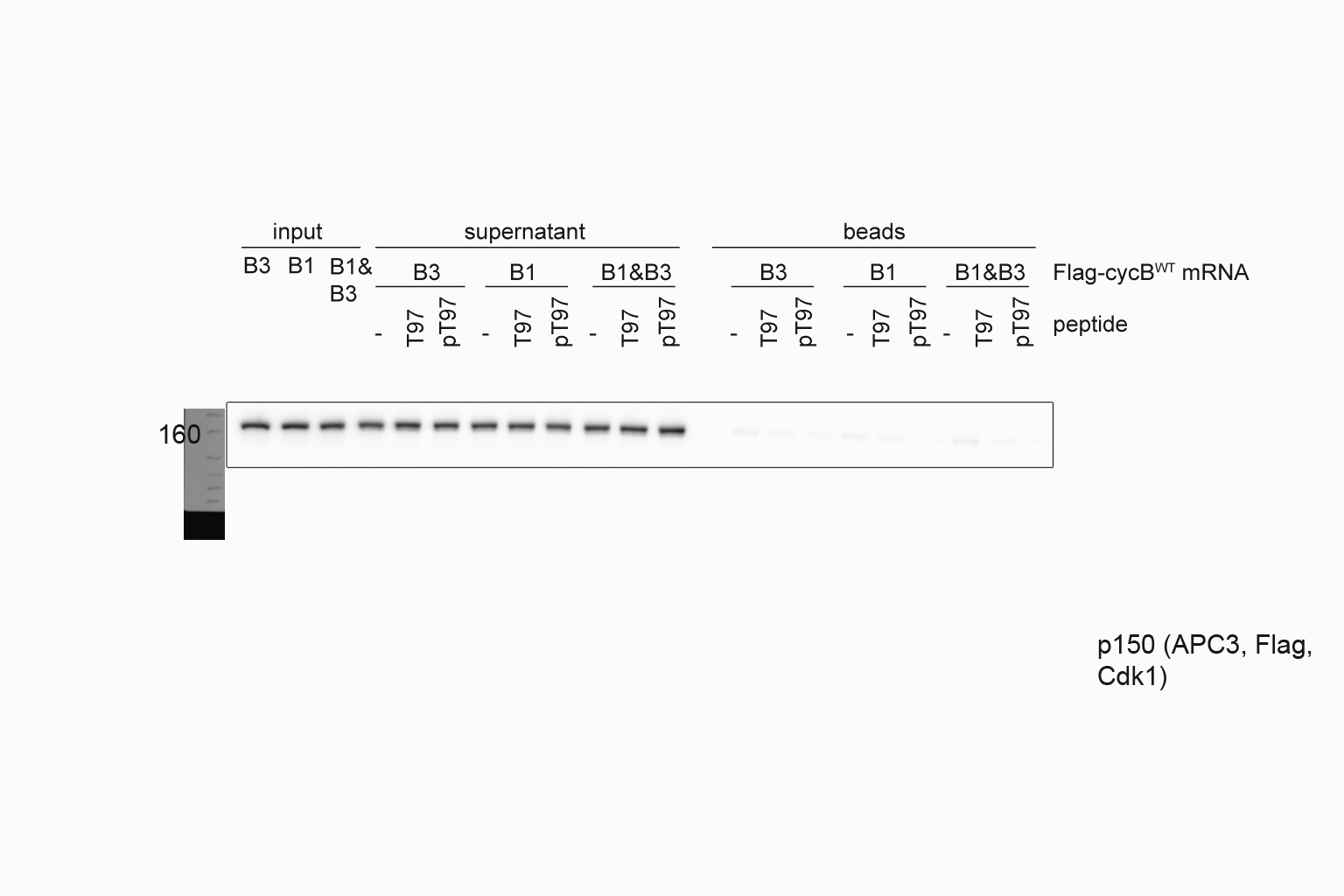

Supplement: Supplementary file 7 — Source data Fig. 6 [file 44319_2024_347_MOESM7_ESM.zip › Figure 6/6A/Western p150 (APC3, Flag, Cdk1).tif]

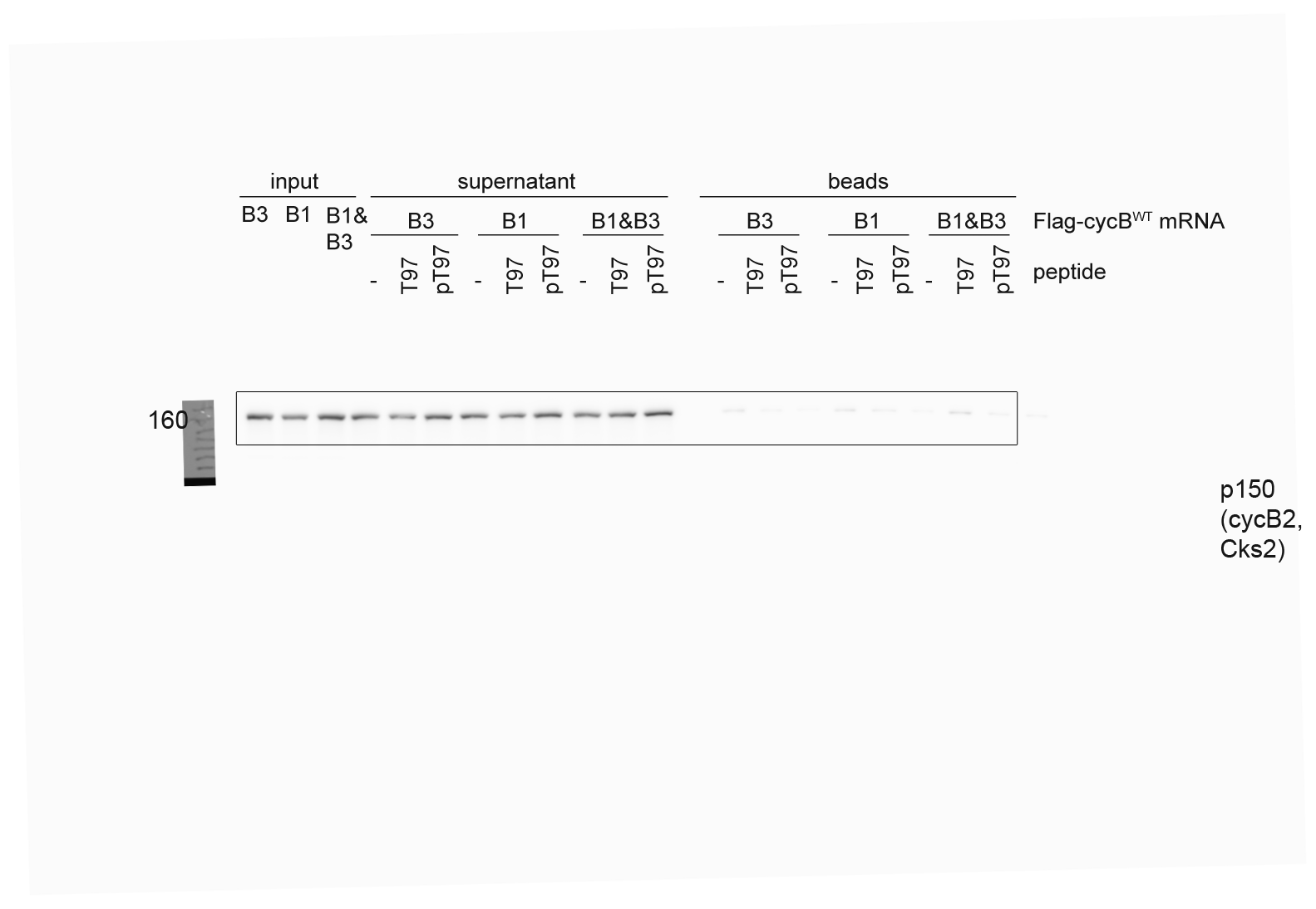

Supplement: Supplementary file 7 — Source data Fig. 6 [file 44319_2024_347_MOESM7_ESM.zip › Figure 6/6A/Western p150 (Cks2, cycB2).tif]

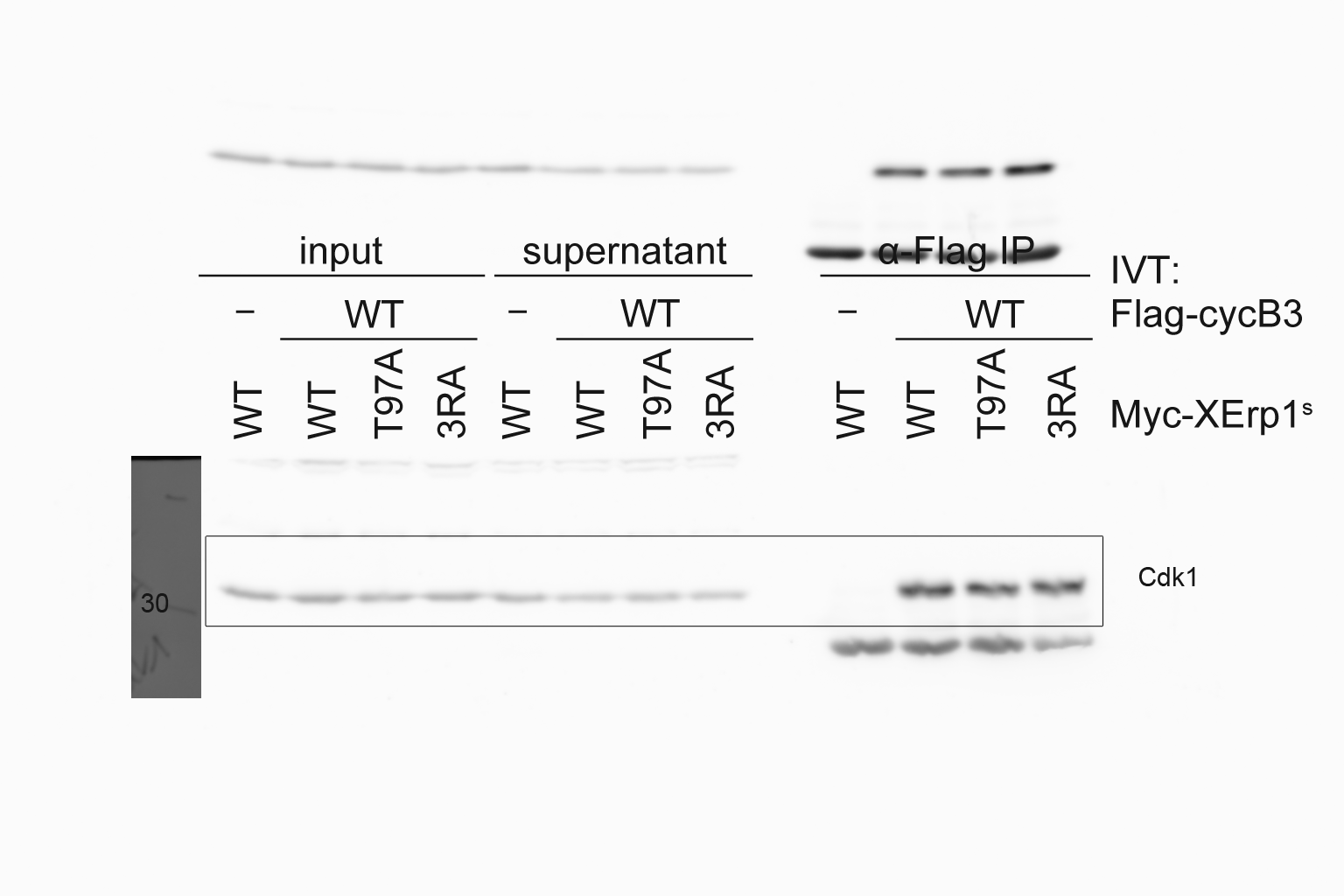

Supplement: Supplementary file 7 — Source data Fig. 6 [file 44319_2024_347_MOESM7_ESM.zip › Figure 6/6C/Western Cdk1.tif]

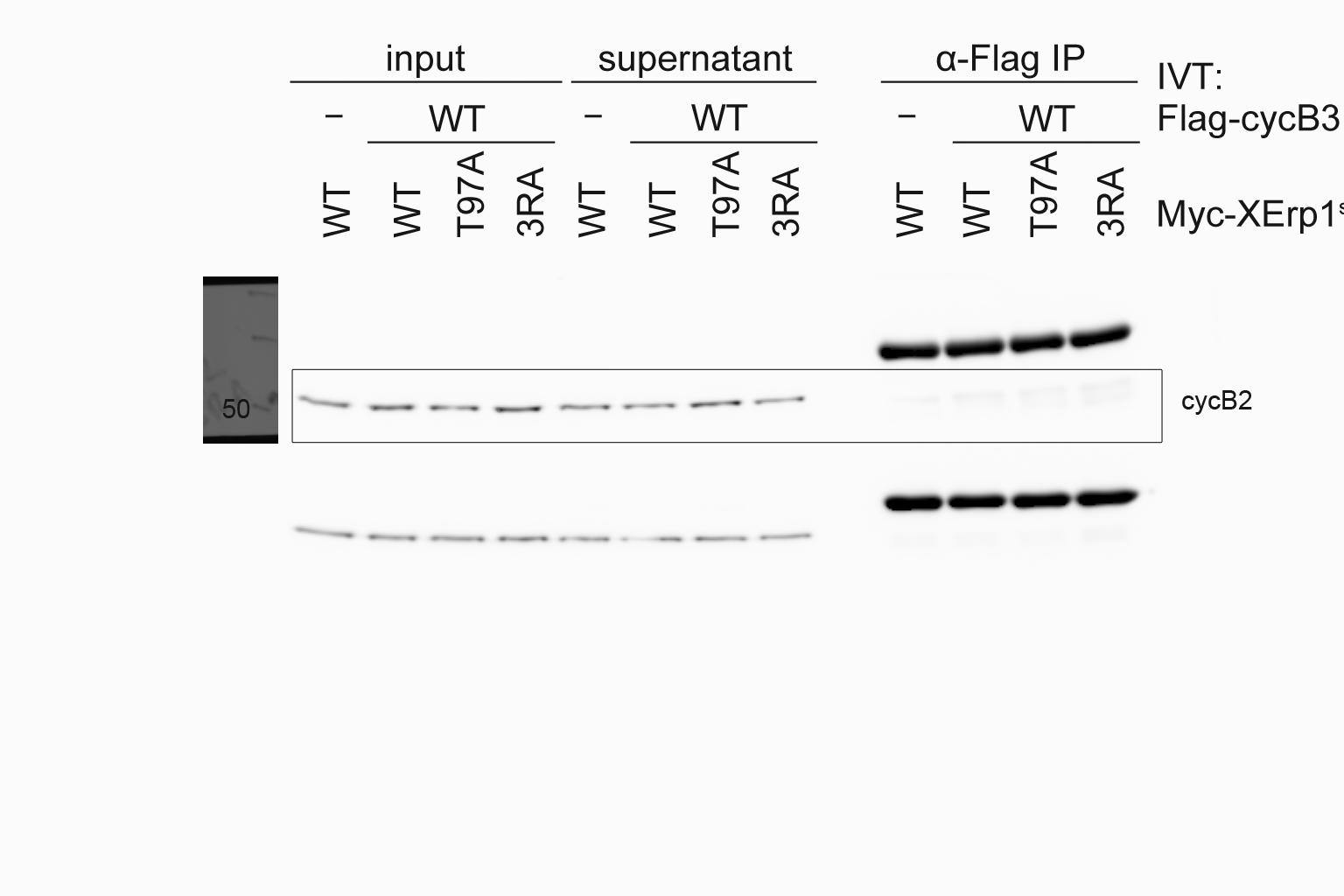

Supplement: Supplementary file 7 — Source data Fig. 6 [file 44319_2024_347_MOESM7_ESM.zip › Figure 6/6C/Western cycB2.tif]

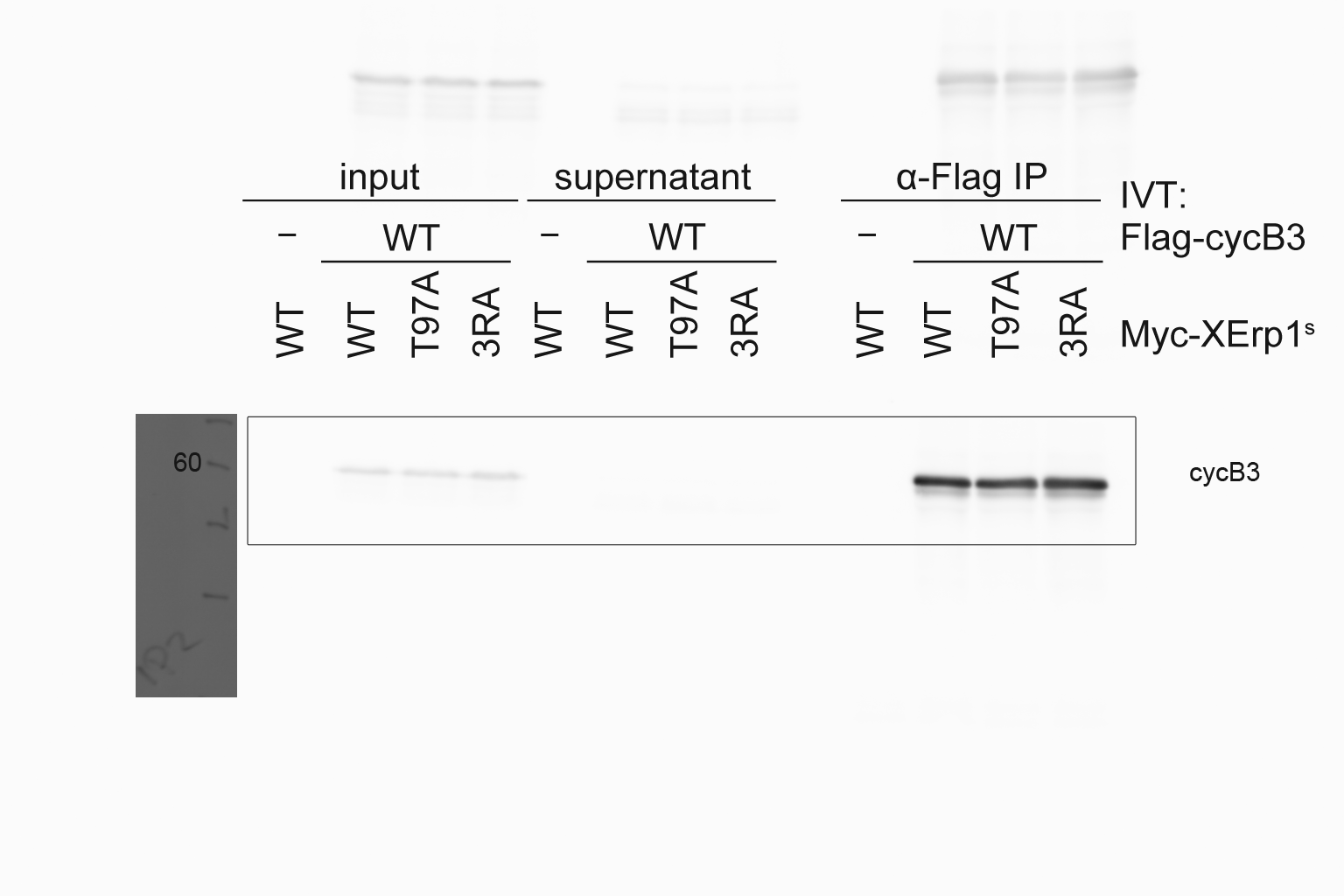

Supplement: Supplementary file 7 — Source data Fig. 6 [file 44319_2024_347_MOESM7_ESM.zip › Figure 6/6C/Western cycB3.tif]

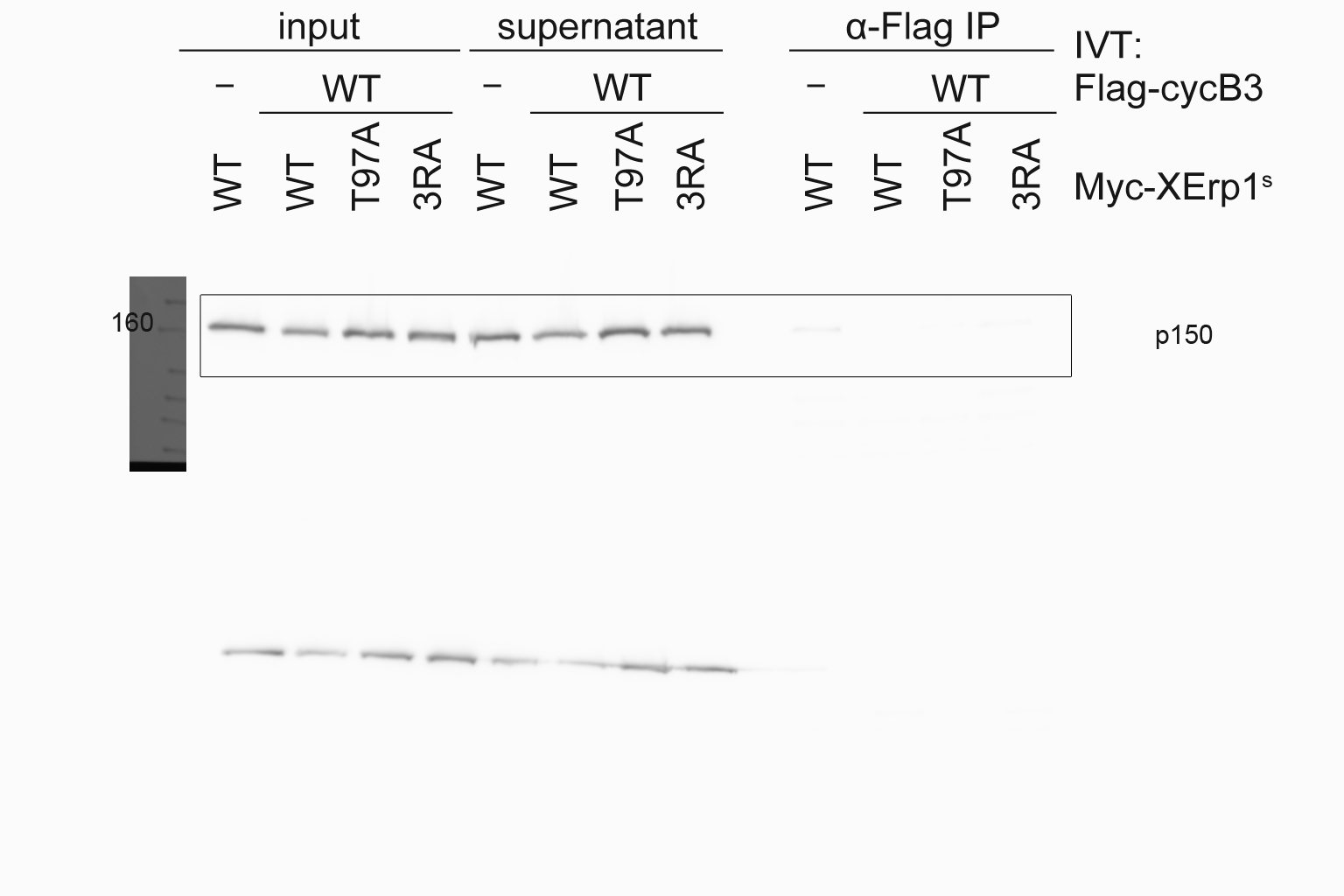

Supplement: Supplementary file 7 — Source data Fig. 6 [file 44319_2024_347_MOESM7_ESM.zip › Figure 6/6C/Western p150.tif]

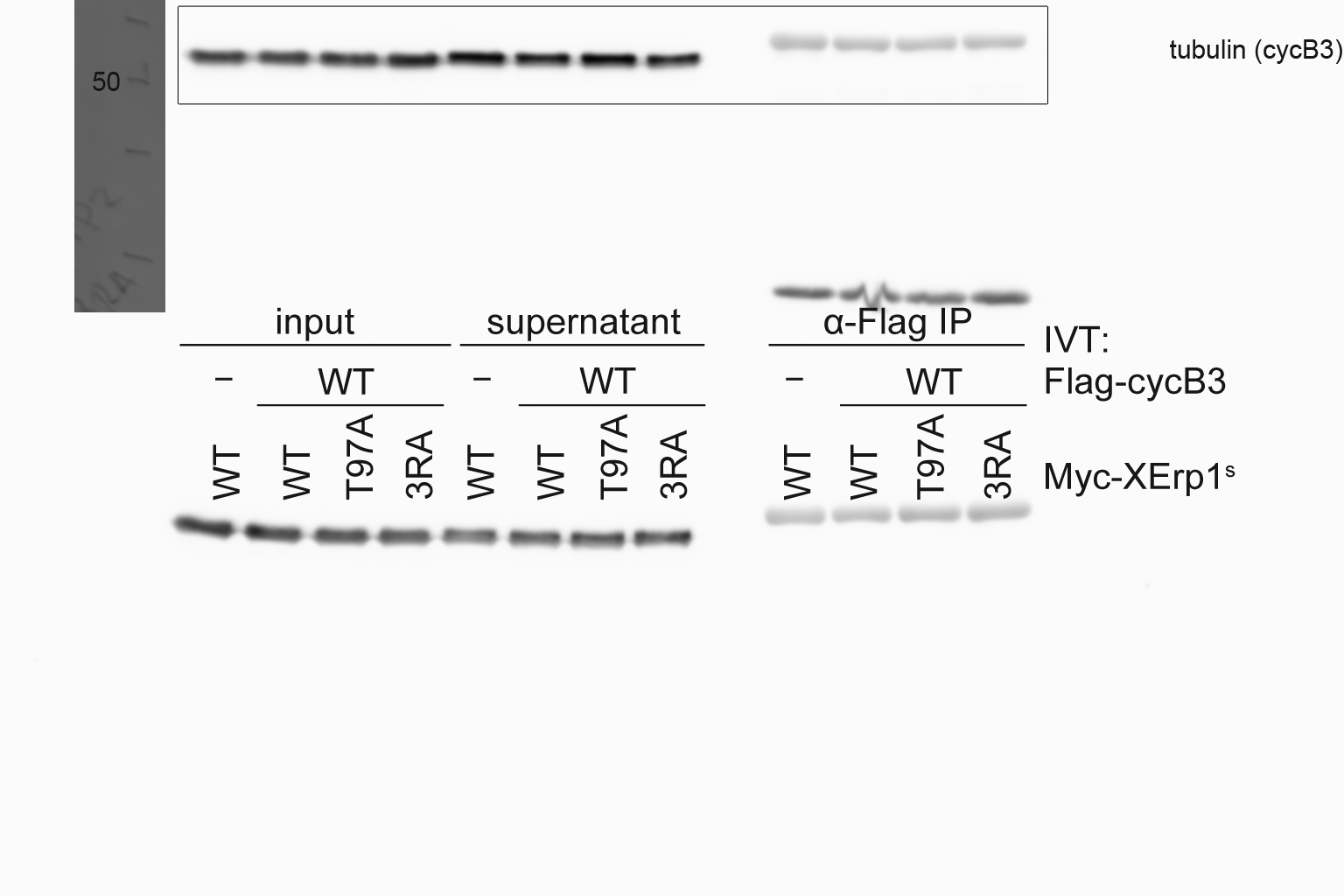

Supplement: Supplementary file 7 — Source data Fig. 6 [file 44319_2024_347_MOESM7_ESM.zip › Figure 6/6C/Western tubulin.tif]

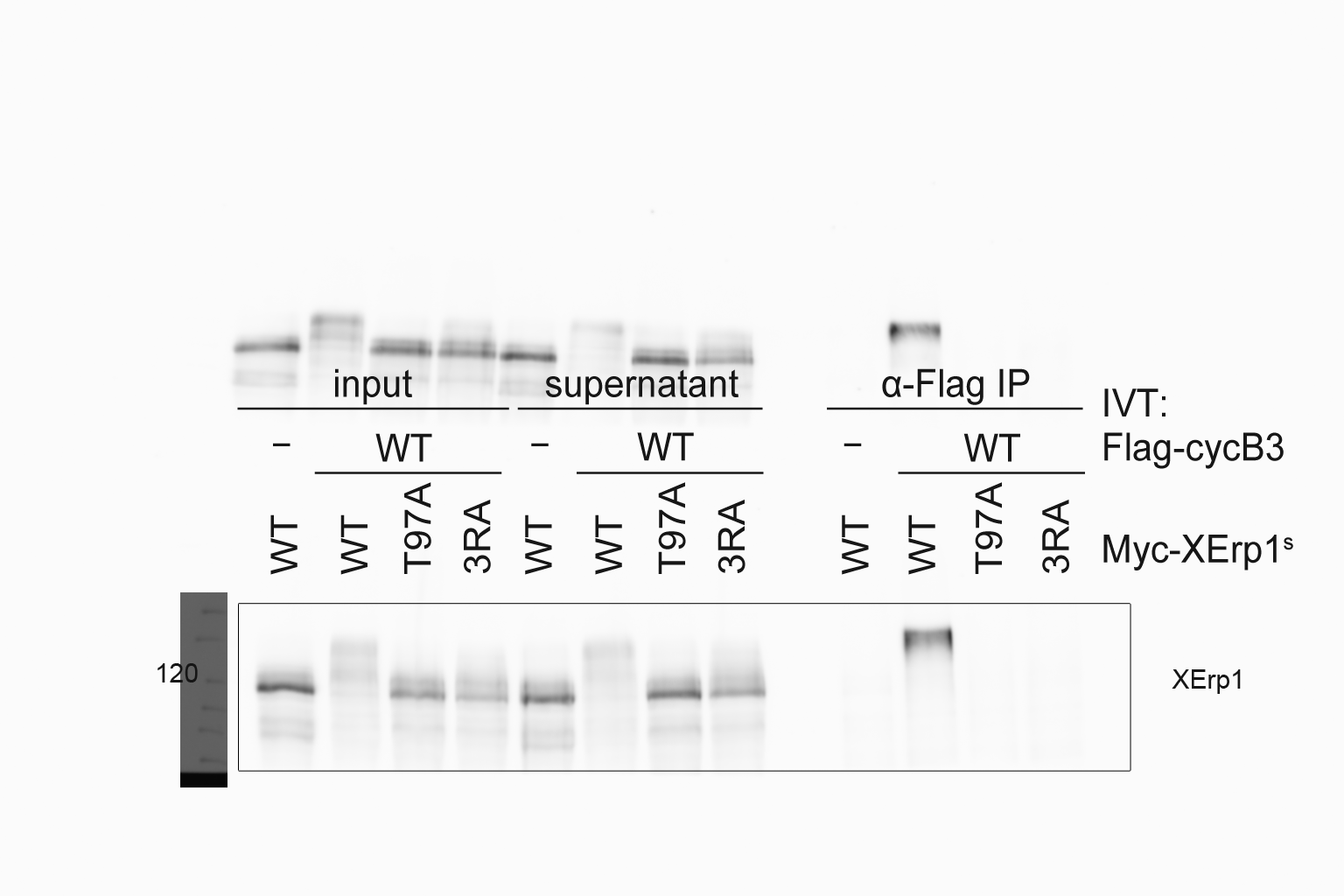

Supplement: Supplementary file 7 — Source data Fig. 6 [file 44319_2024_347_MOESM7_ESM.zip › Figure 6/6C/Western XErp1.tif]

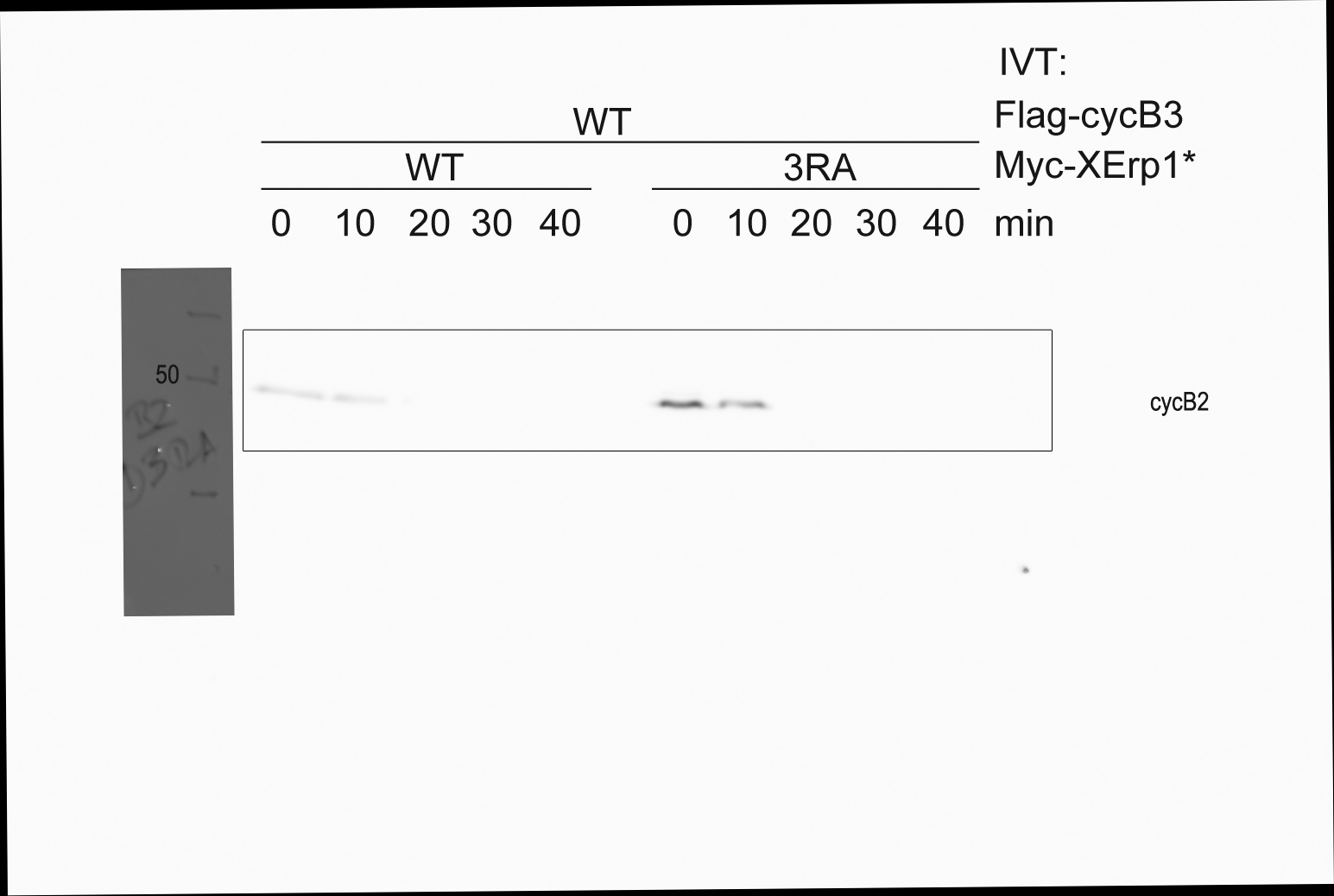

Supplement: Supplementary file 7 — Source data Fig. 6 [file 44319_2024_347_MOESM7_ESM.zip › Figure 6/6D/Western cycB2.tif]

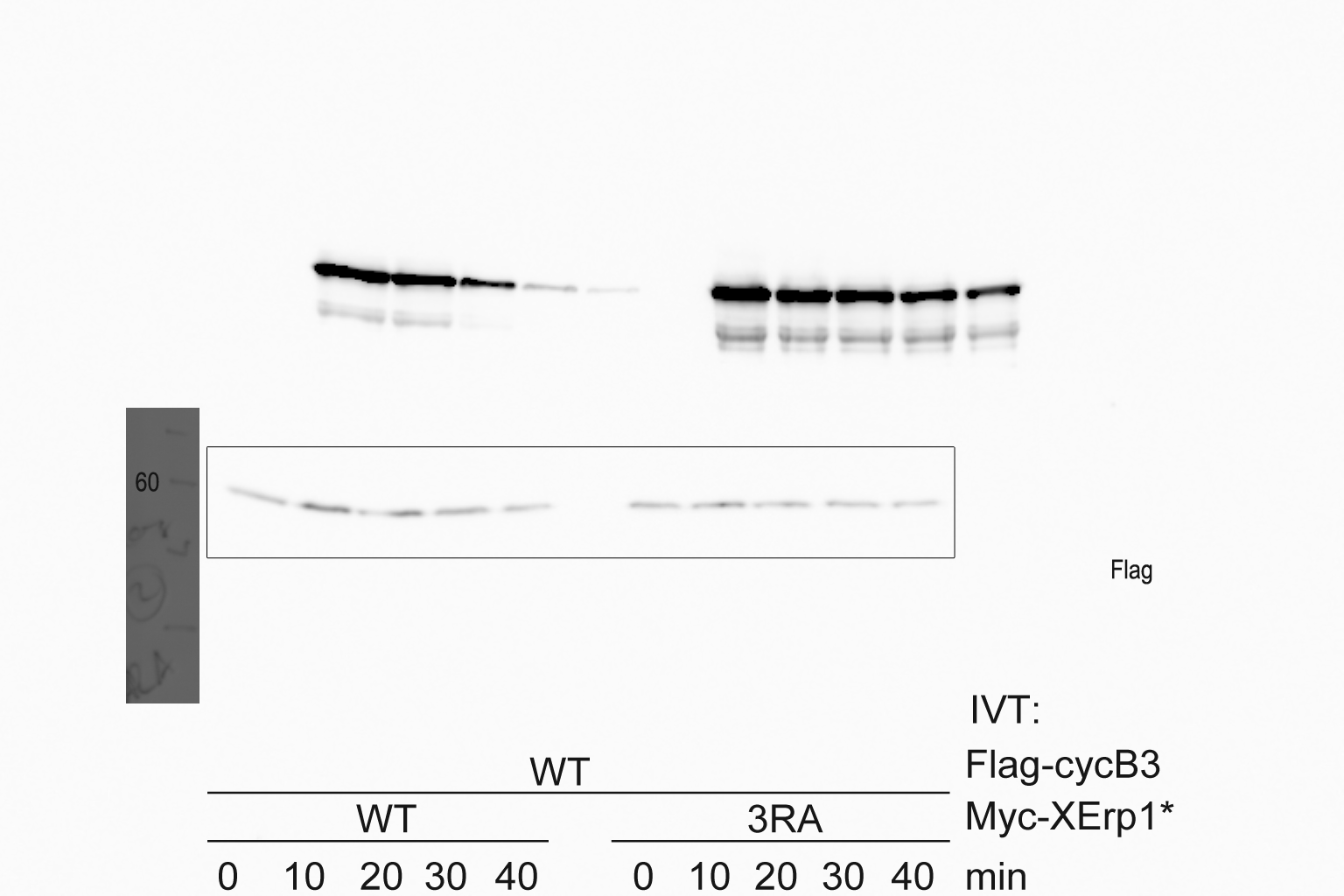

Supplement: Supplementary file 7 — Source data Fig. 6 [file 44319_2024_347_MOESM7_ESM.zip › Figure 6/6D/Western Flag.tif]

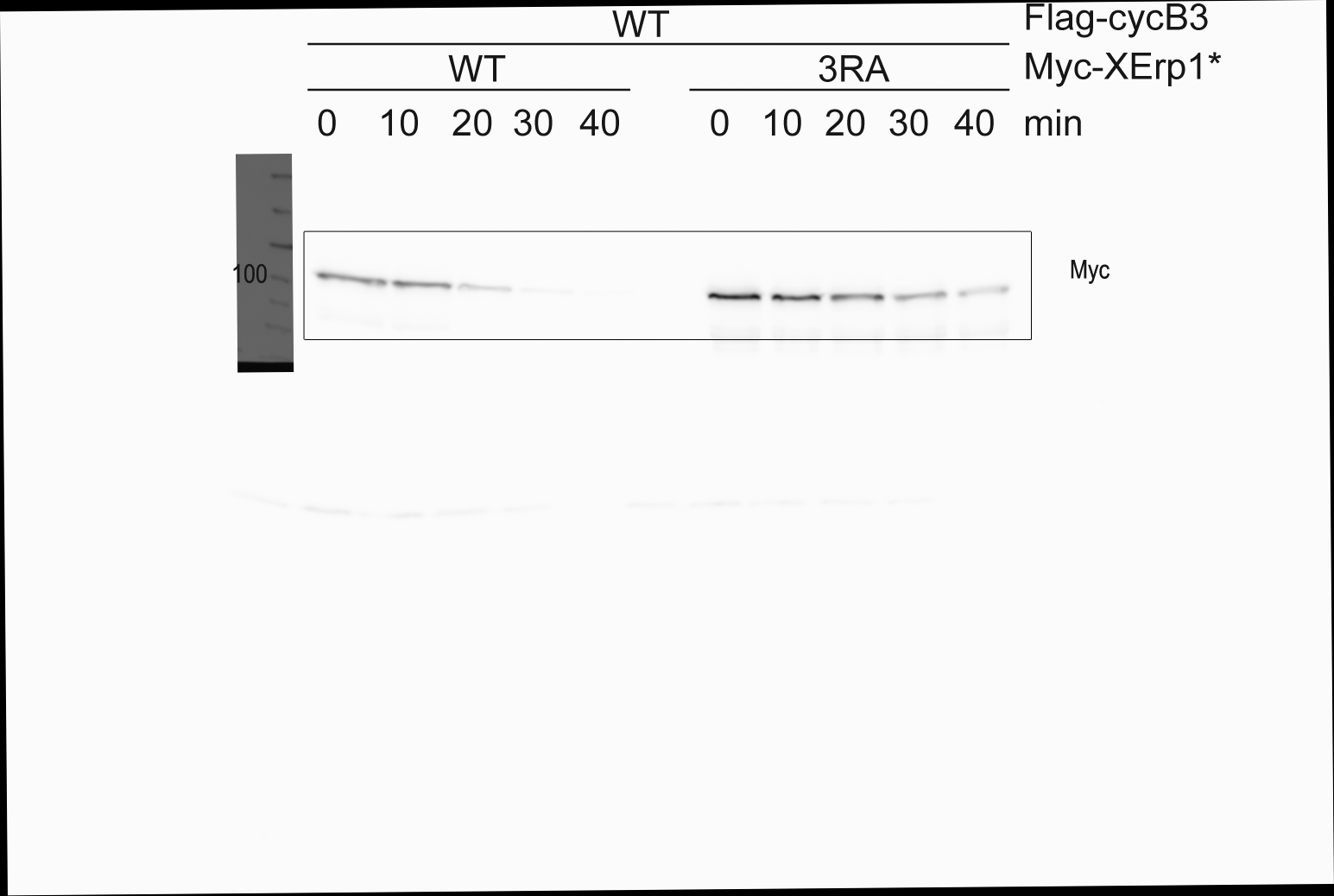

Supplement: Supplementary file 7 — Source data Fig. 6 [file 44319_2024_347_MOESM7_ESM.zip › Figure 6/6D/Western Myc.tif]
