# Supplementary figures and images for: A phosphate-binding pocket in cyclin B3 is essential for XErp1/Emi2 degradation in meiosis I (part 2 of 2)
Source: EMBO Rep. 2025 Jan 2;26(3):768–90. doi: 10.1038/s44319-024-00347-8 (PMC11811201; doi:10.1038/s44319-024-00347-8)

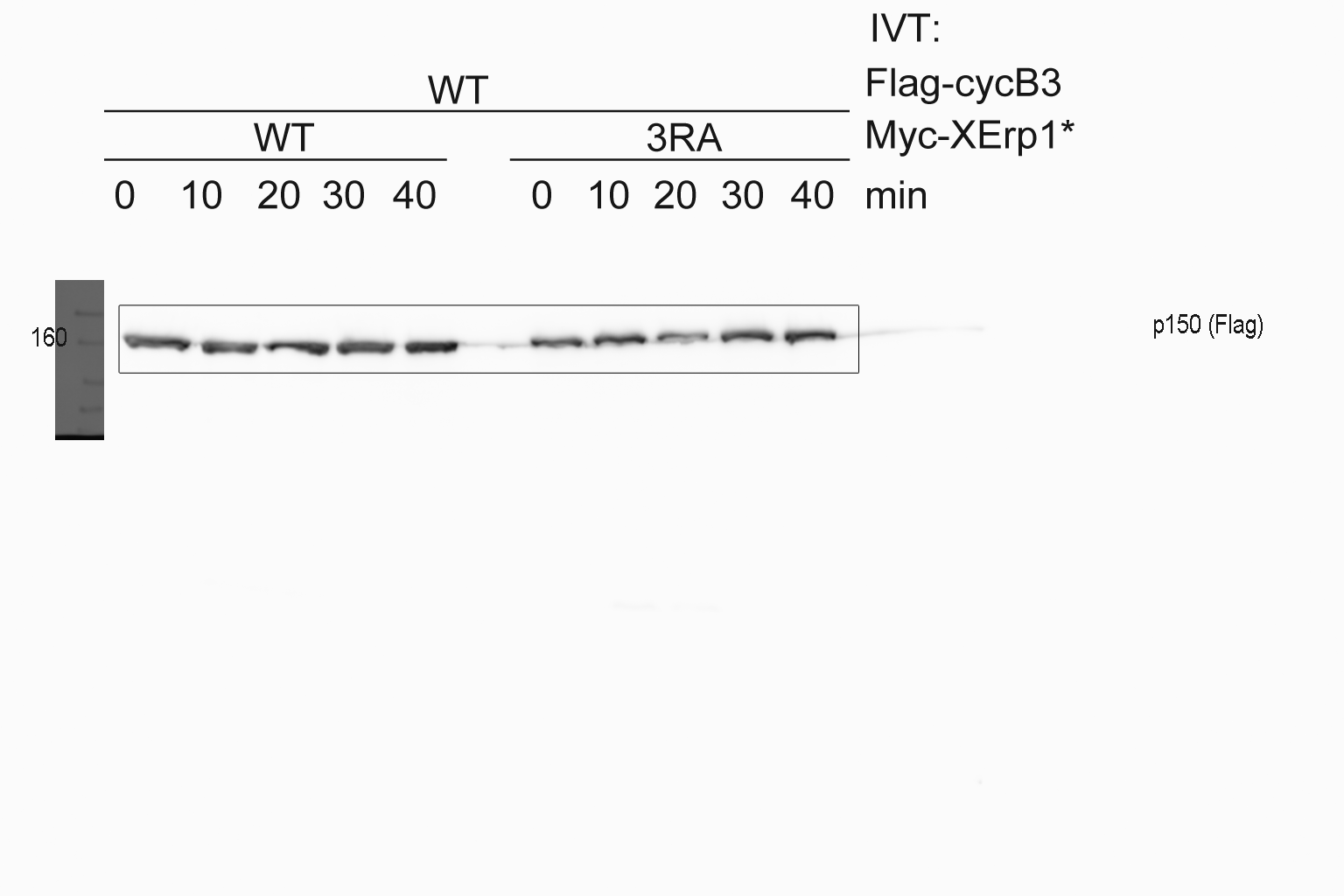

Supplement: Supplementary file 7 — Source data Fig. 6 [file 44319_2024_347_MOESM7_ESM.zip › Figure 6/6D/Western p150 (Flag).tif]

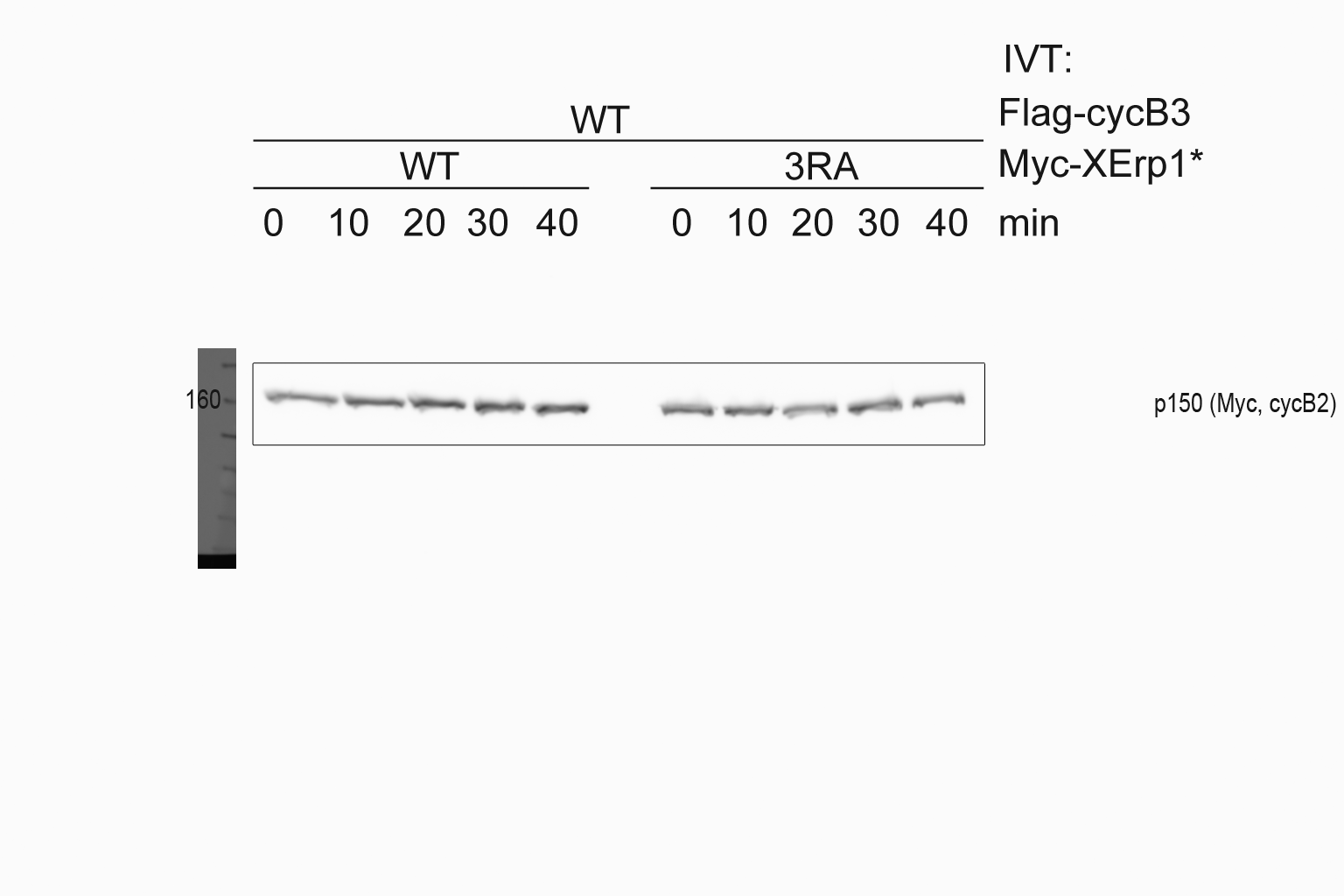

Supplement: Supplementary file 7 — Source data Fig. 6 [file 44319_2024_347_MOESM7_ESM.zip › Figure 6/6D/Western p150 (Myc, cycB2).tif]

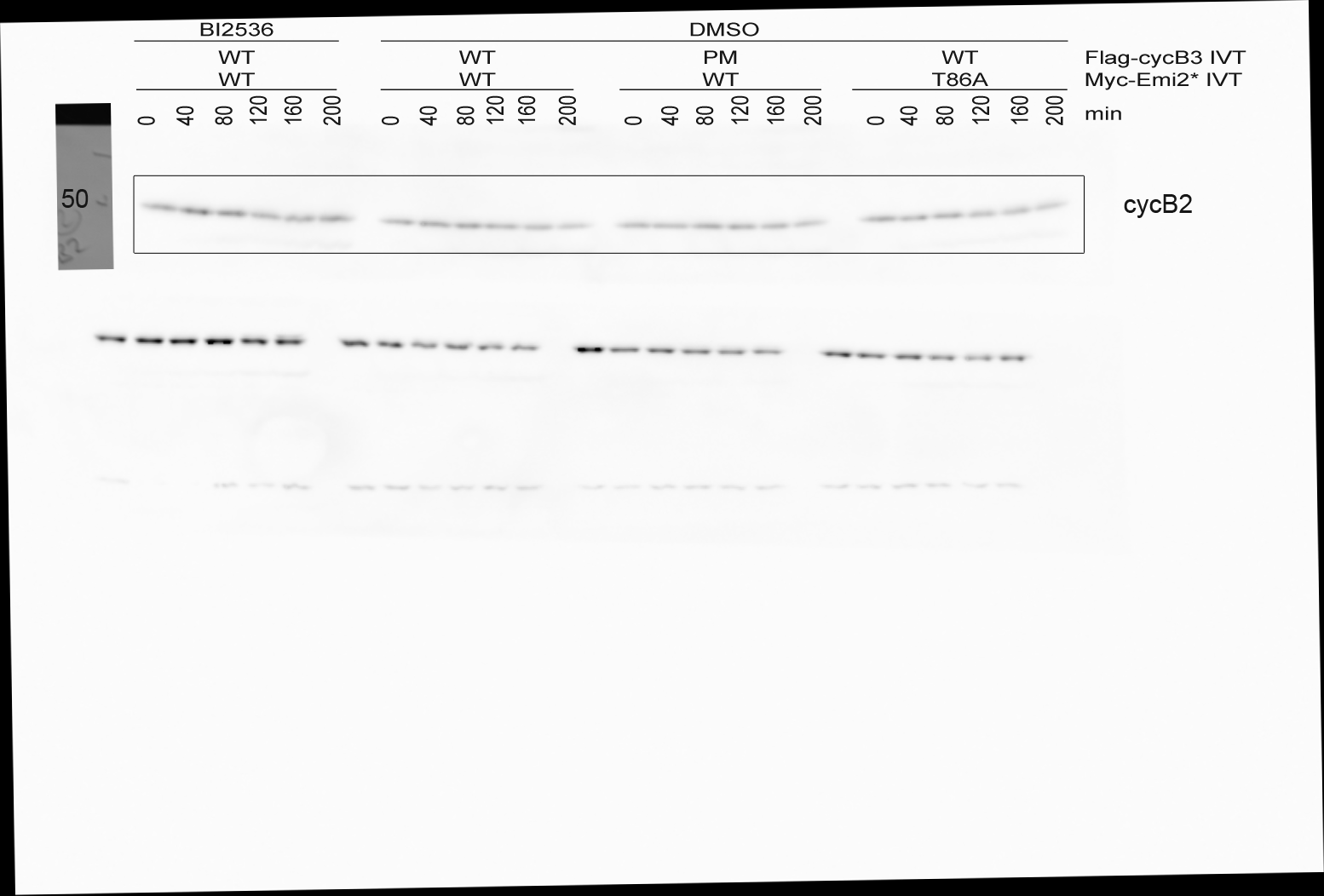

Supplement: Supplementary file 8 — Source data Fig. 7 [file 44319_2024_347_MOESM8_ESM.zip › Figure 7/7C/Western cycB2.tif]

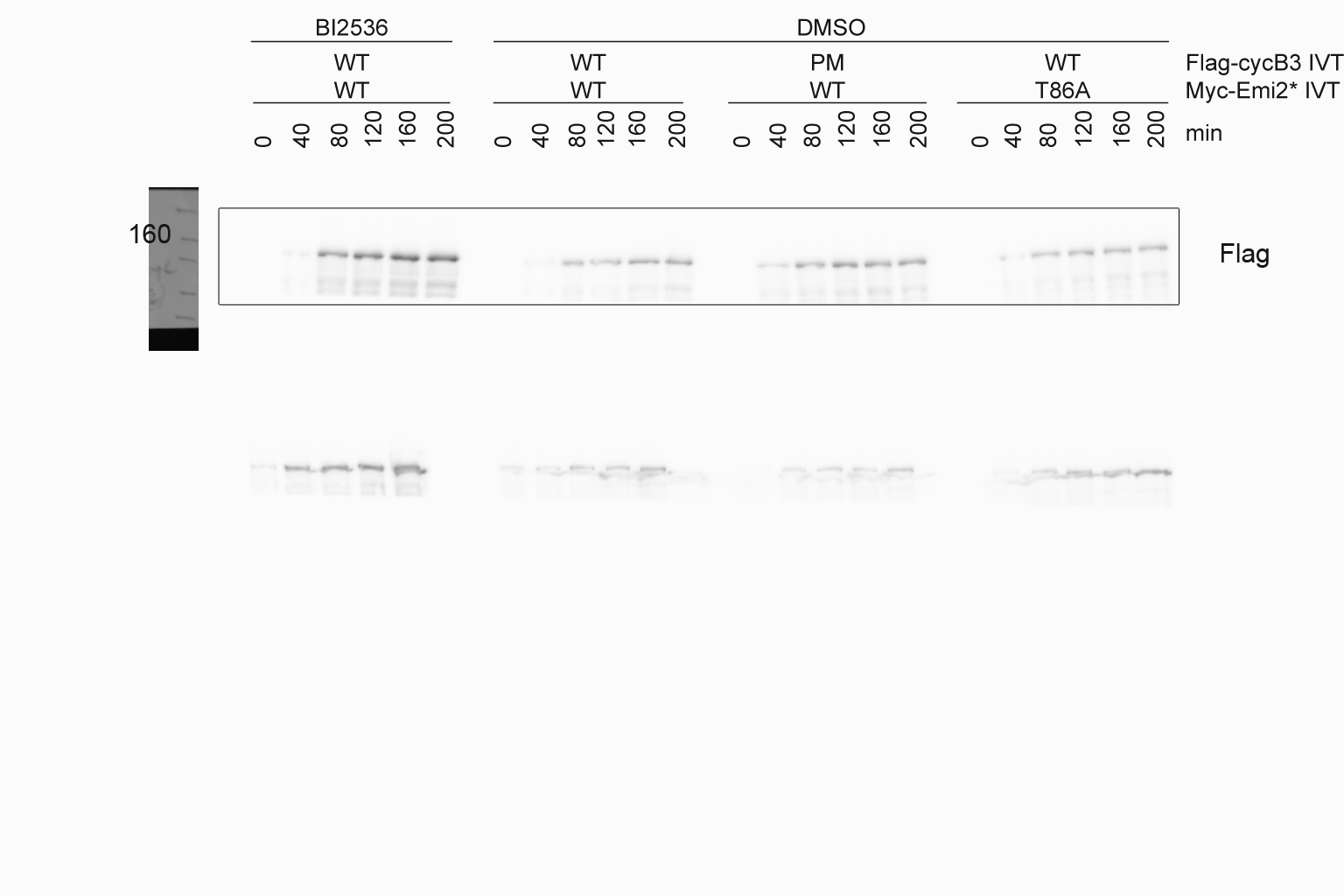

Supplement: Supplementary file 8 — Source data Fig. 7 [file 44319_2024_347_MOESM8_ESM.zip › Figure 7/7C/Western Flag.tif]

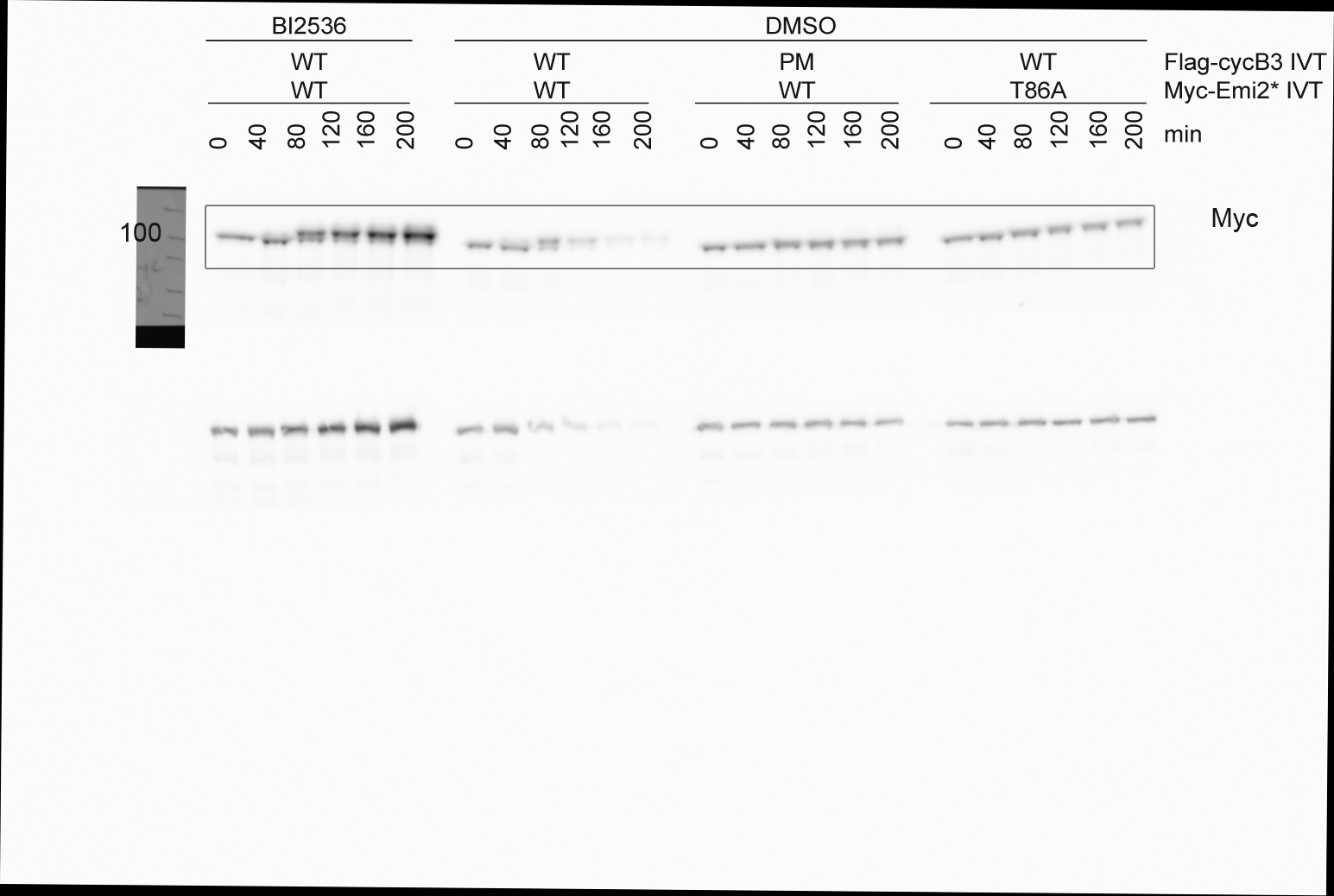

Supplement: Supplementary file 8 — Source data Fig. 7 [file 44319_2024_347_MOESM8_ESM.zip › Figure 7/7C/Western Myc.tif]

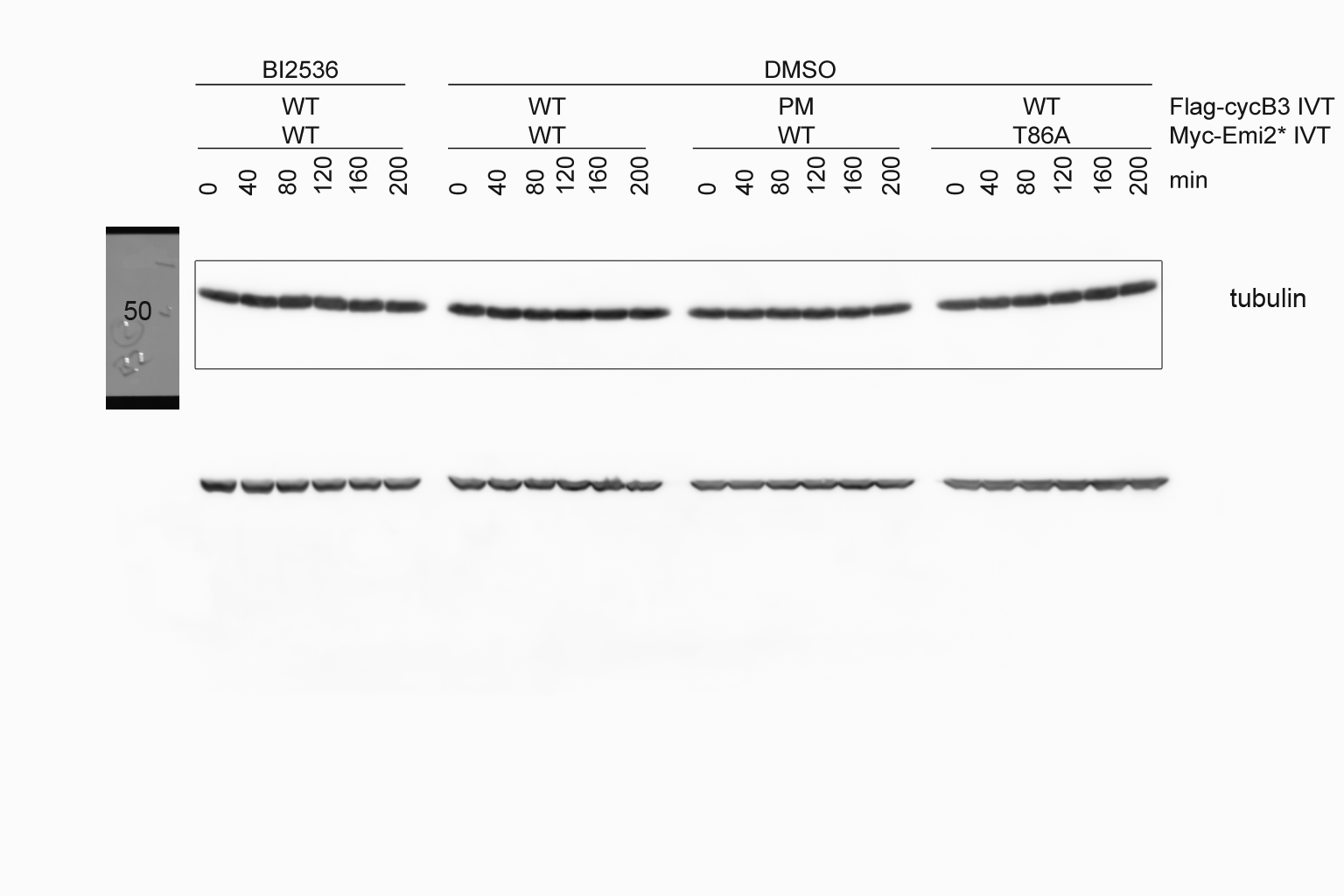

Supplement: Supplementary file 8 — Source data Fig. 7 [file 44319_2024_347_MOESM8_ESM.zip › Figure 7/7C/Western tubulin.tif]
