# Supplementary material for: A phosphate-binding pocket in cyclin B3 is essential for XErp1/Emi2 degradation in meiosis I
Source: EMBO Rep. 2025 Jan 2;26(3):768–90. doi: 10.1038/s44319-024-00347-8 (PMC11811201; doi:10.1038/s44319-024-00347-8)
Supplement: Supplementary file 9 — Expanded View Figures [file 44319_2024_347_MOESM9_ESM.pdf]

Expanded View Figures

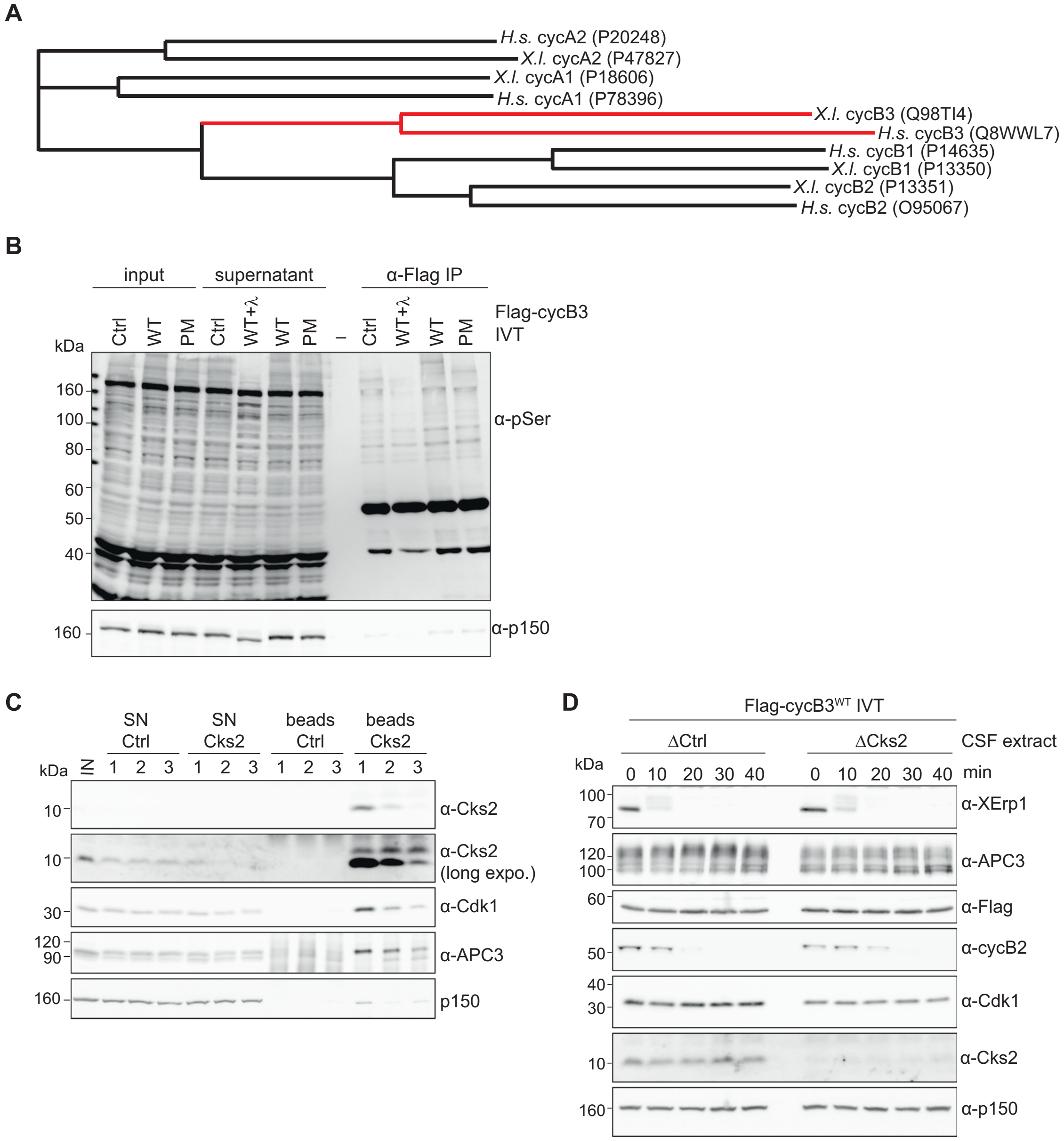

Figure EV1. Phylogenetic tree of A- and B-type cyclins, pSer signal of cycB3$^{WT/PM}$ IP and XErp1 degradation in Cks2 depleted CSF extract.

(**A**) Phylogenetic tree of human (*H.s.*) and Xenopus (*X.l.*) cyclin (cyc) A1, cyclin A2, and B-type cyclins (B1-3) created within UniProt. In brackets, UniProt number of respective proteins are indicated. (**B**) pSer WB analysis of α-Flag IP samples shown in Fig. 1D. CSF extract was supplemented with IVT Flag-cyclin B3$^{WT/PM}$ and the meiotic state of the extract was maintained by the addition of MG262 and okadaic acid. Indicated samples were treated with λ-phosphatase (+ λ). p150 served as a loading control. One representative experiment from three independent biological replicates is shown. (**C**) WB analysis of Cks2 depletion experiment. Cks2 was depleted from CSF extract by three rounds of α-Cks2 immunoprecipitations. Control (Ctrl) depletion was performed using unspecific IgG antibodies. Shown are input (IN), supernatant (SN) and bead samples. p150 served as loading control. (**D**) Control depleted or Cks2 depleted CSF extract was supplemented with IVT of Flag-cyclin B3^{WT}. Samples were taken at indicated time points and immunoblotted. One representative experiment from three biological replicates is shown. p150 served as loading control.

© The Author(s)

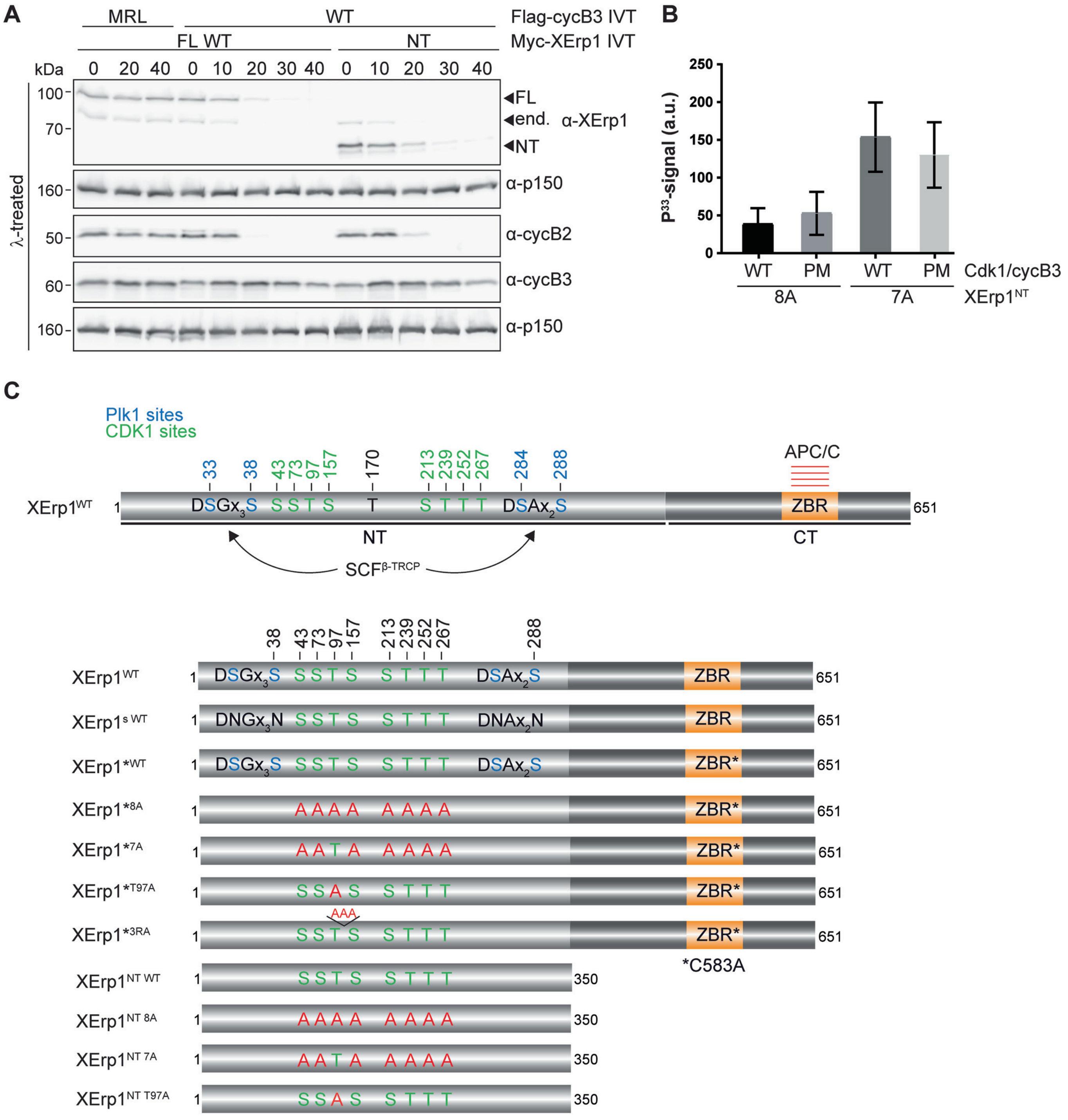

Figure EV2. Degradation behaviour of XErp1 N-terminal fragment, quantification of T97 phosphorylation by Cdk1/cycB3$^{WT/PM}$ and schemes of XErp1 variants.

(**A**) CSF extract was supplemented with Flag-cyclin B3$^{WT/MRL}$ and full-length (FL) or NT Myc-XErp1. Samples were taken at indicated time points and immunoblotted. p150 served as loading control. One representative experiment from two biological replicates is shown. (**B**) Quantification of experiment shown in Fig. 3D and two further replicates. The ^{33}P signal was quantified and normalized to Cdk1 levels detected by WB. Mean ± SD is depicted (*n* = 3). (**C**) Illustration of XErp1 variants used for in vitro kinase assays (Figs. 3D and 5A) and experiments shown in Figs. 3D, 4C, 5A–C and 6C, D. XErp1^{s} is a stable variant due to the mutation of both phosphodegrons ($DSGx_3S^{38}$→$DNGx_3N^{38}$ and $DSAx_2S^{288}$→$DNAx_2N^{288}$). XErp1* is deficient in APC/C inhibition due to a mutation in the zinc-binding region (ZBR, C583A). NT N-terminus, CT C-terminus.

 © The Author(s)

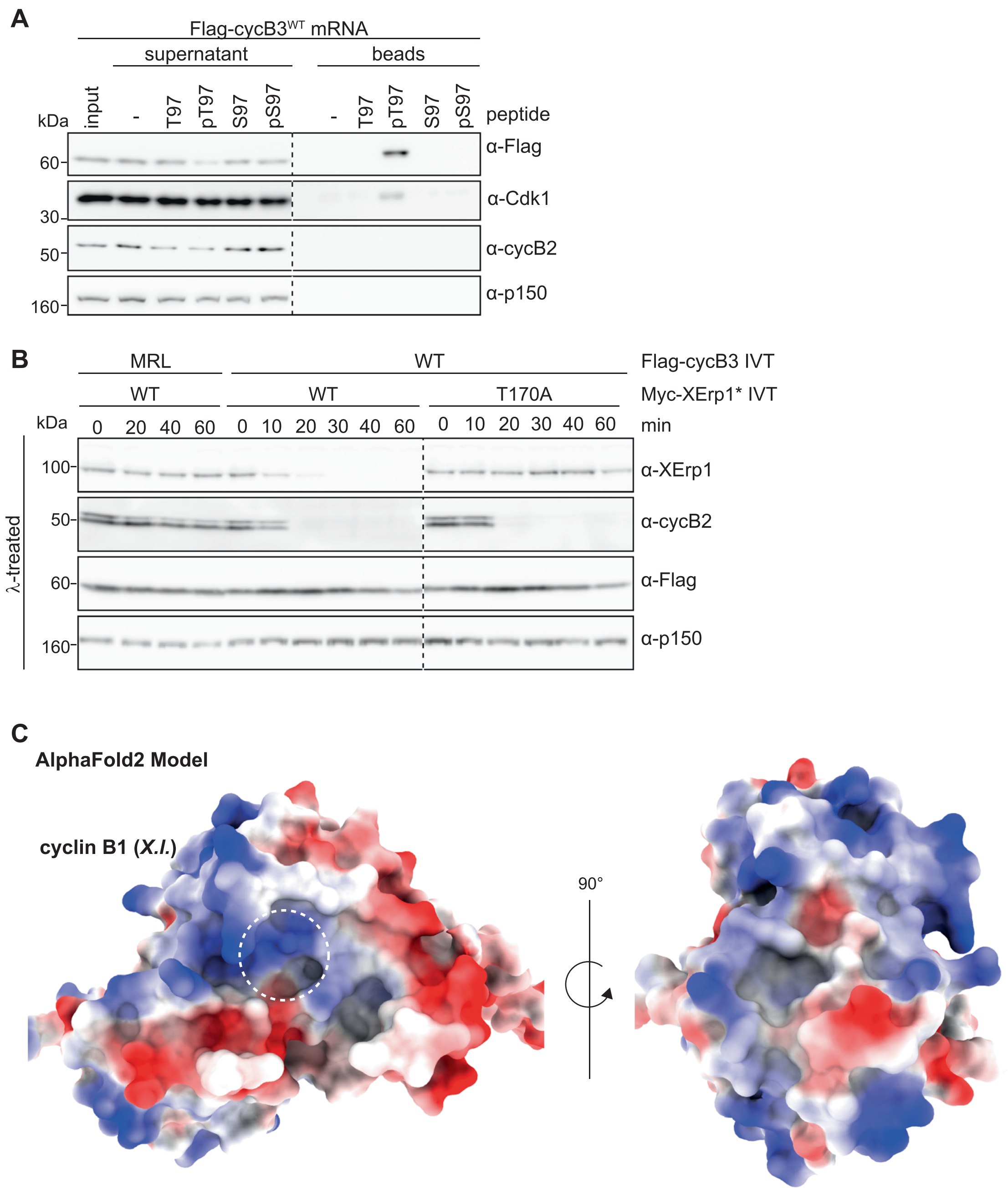

Figure EV3. CycB3 phosphate-binding pocket interacts only with XErp1 pT97 but not pS97, XErp1 T170A mutation influence cyclin B3-induced XErp1 degradation and surface charge map of cyclin B1.

(**A**) Bead-coupled XErp1 peptides (aa 90–104) containing unphosphorylated T97 (T97), phosphorylated T97 (pT97), unphosphorylated S97 (S97), or phosphorylated S97 (pS97) were incubated in CSF extract supplemented with mRNA encoding Flag-cyclin B3^{WT}. Exit from MII was prevented by the addition of MG262 and IVT XErp1^{CT}. Input, supernatant, and pull-downed beads were immunoblotted. p150 served as loading control. One representative experiment from three biological replicates is shown. (**B**) CSF extract was supplemented with IVT Myc-XErp1* WT or T170A variants and Flag-cyclin B3$^{WT/MRL}$. XErp1* is deficient in APC/C inhibition (see EV2C) to prevent that it interferes with meiotic exit. At indicated time points, samples were taken, treated with lambda phosphatase (λ) and immunoblotted. p150 served as loading control. (**C**) Surface charge map of AlphaFold2 model of *Xenopus* cyclin B1 in the corresponding position as cyclin B3 shown in Fig. 6B. White dashed circle highlights the phosphate binding pocket.
